# Bioinformatics Analysis Identifies Lipid Droplet‐Associated Gene Signatures as Promising Prognostic and Diagnostic Models for Endometrial Cancer

**DOI:** 10.1002/cnr2.70313

**Published:** 2025-08-13

**Authors:** Vijayalakshmi N. Ayyagari, Miao Li, Paula Diaz‐Sylvester, Kathleen Groesch, Teresa Wilson, Ejaz M. Shah, Laurent Brard

**Affiliations:** ^1^ Division of Gynecologic Oncology, Department of Obstetrics and Gynecology Southern Illinois University School of Medicine Springfield Illinois USA; ^2^ Simmons Cancer Institute Southern Illinois University School of Medicine Springfield Illinois USA; ^3^ Center for Clinical Research Southern Illinois University School of Medicine Springfield Illinois USA

**Keywords:** diagnostic models, endometrial cancer, lipid droplets, prognostic models

## Abstract

**Background:**

Effective diagnostic and prognostic tools are critical for early detection and improved outcomes in endometrial cancer (EC). Although metabolic dysregulation plays a key role in EC pathogenesis, the clinical relevance of lipid droplet–associated genes (LDAGs) remains largely unexplored. This study aims to establish LDAG‐based gene signatures with strong diagnostic and prognostic potential in EC.

**Aims:**

To identify LDAG signatures with prognostic and diagnostic utility in EC.

**Methods and Results:**

A curated set of LDAGs was systematically analyzed across publicly available EC datasets to identify differentially expressed LDAGs (DE‐LDAGs). Survival‐associated DE‐LDAGs were then identified using univariate Cox regression. A four‐gene prognostic model was developed through LASSO‐based feature selection followed by multivariate Cox regression and validated using Kaplan–Meier survival and time‐dependent receiver operating characteristic (ROC) analyses. From the same pool of survival‐associated DE‐LDAGs, a six‐gene diagnostic model was constructed using LASSO, ROC analysis, and logistic regression. Model performance was evaluated using ROC curves and support vector machine (SVM) classification. Functional enrichment and protein–protein interaction (PPI) network analyses were conducted to assess the biological relevance of the identified genes.

Our results demonstrate that the four‐gene prognostic model (LMLN, LMO3, PRKAA2, and RAB10) stratified EC patients into high‐ and low‐risk groups with significantly different survival outcomes (*p* < 0.05; time‐dependent AUC > 0.70). The six‐gene diagnostic model (AIFM2, ABCG1, LIPG, DGAT2, LPCAT1, and VCP) demonstrated near‐perfect classification of tumor versus normal tissues (AUC ≈0.99 in ROC analysis; 99.8% accuracy in SVM analysis). Functional enrichment linked DE‐LDAGs to lipid metabolism, ER stress response, cholesterol homeostasis, and autophagy, underscoring their biological relevance in EC pathobiology.

**Conclusion:**

This study provides the first comprehensive analysis of LDAGs in EC, establishing robust prognostic and diagnostic gene signatures with strong biological relevance. These signatures support a metabolism‐driven framework for EC classification and may offer potential clinical utility in early detection, risk stratification, and personalized treatment.

**Abbreviations**
ABC: ATP‐binding cassette
Akt: protein kinase B
AMPK: AMP‐activated protein kinase
AUC: area under the curve
BMI: body mass index
CA‐125: cancer antigen 125
ChEA3: ChIP enrichment analysis 3
CPTAC: clinical proteomic tumor analysis consortium
DAVID: database for annotation, visualization, and integrated discovery
DE‐LDAGs: differentially expressed lipid droplet associated genes
EC: endometrial cancer
FDR: false discovery rate
FPKM‐UQ: fragments per kilobase of transcript per million mapped reads—upper quartile normalized
GAPDH: glyceraldehyde‐3‐phosphate dehydrogenase
GEO: gene expression omnibus
GEPIA2: gene expression profiling interactive analysis 2
GO: gene ontology
GSCA: gene set cancer analysis
HR: hazard ratio
IL‐6: interleukin 6
KEGG: Kyoto encyclopedia of genes and genomes
KOBAS: KEGG orthology based annotation system
LASSO: least absolute shrinkage and selection operator
LDAGs: lipid droplet associated genes
LDs: lipid droplets
MCODE: molecular complex detection
OS: overall survival
PI3K: phosphoinositide 3‐kinase
POLE: polymerase epsilon
PPI: protein–protein interaction
ROC: receiver operating characteristic
STRING: search tool for the retrieval of interacting genes
SVM: support vector machine
TCGA: the cancer genome atlas
TNF: tumor necrosis factor
TPM: transcripts per million
UALCAN: University of Alabama at Birmingham Cancer data analysis Portal
UCEC: uterine corpus endometrial carcinoma

## 1 Introduction

Endometrial cancer (EC) is the most common gynecologic malignancy in developed countries, with an estimated 69 120 new cases and approximately 13 860 deaths projected in the United States in 2025 [1, 2, 3]. Although 67% of EC cases are diagnosed at an early stage with favorable 5‐year overall survival (OS), outcomes decline markedly in advanced disease, with 5‐year OS falling to 17% in stage IVA, 15% in stage IVB, respectively [4]. Prognostic stratification has advanced through the integration of traditional clinical factors such as age, stage, and tumor grade, with TCGA‐defined molecular subtypes including POLE (*polymerase epsilon)*, microsatellite instability‐high (MSI‐H), copy‐number low, and copy‐number high categories [5, 6, 7, 8]. Additional biomarkers, such as p53 mutations, *β*‐catenin alterations, HER2 amplification, and patient age, have further refined risk assessment [5, 6, 7, 8]. However, despite these advances, substantial variability in outcomes persists, reflecting the biological and clinical heterogeneity of EC. This underscores the need for novel multi‐marker models to improve risk stratification and enable personalized management.

Despite the strong association between early detection and improved survival in EC, accurate diagnosis remains challenging. Current approaches, including endometrial biopsy, transvaginal ultrasound, and serum CA‐125, are invasive, operator dependent, and often lack sensitivity for early stage or diagnostically ambiguous cases [7, 8, 9]. These limitations highlight the need for more precise and minimally invasive diagnostic strategies.

Metabolic dysfunction has emerged as a defining feature of EC pathogenesis, with epidemiologic studies linking obesity, diabetes, and dyslipidemia to a two‐ to sixfold increased risk of EC [7, 8, 10]. Consistent with these associations, transcriptomic and proteomic profiling of EC tumors has revealed widespread metabolic reprogramming, particularly involving lipid synthesis and storage [11, 12, 13, 14]. The PI3K/Akt/mTOR pathway (PAM), commonly dysregulated in EC, drives expression of lipogenic regulators such as SREBP1, ACC, and FASN, thereby promoting intracellular lipid accumulation [15, 16, 17, 18]. These alterations contribute to the formation of lipid droplets (LDs), which sequester neutral lipids and protect tumor cells from lipotoxic stress. LD accumulation has been associated with malignancy, tumor aggressiveness, immune evasion, chemoresistance, and metastasis across multiple malignancies [19, 20, 21, 22].

LDs are increasingly recognized as dynamic organelles that regulate lipid homeostasis, mitigate oxidative stress, support autophagy, and inhibit ferroptosis [22, 23, 24, 25, 26]. These functions are essential for tumor cell survival under metabolic stress. Consistent with these roles, elevated LD accumulation has been observed in various cancers, highlighting their potential as diagnostic and prognostic biomarkers [21, 22, 23, 24, 25, 26, 27]. Overexpression of lipid droplet–associated proteins, particularly perilipins (PAT family) and diacylglycerol acyltransferases (DGATs), has been associated with malignant progression, increased tumor aggressiveness, and poor clinical outcomes across multiple cancer types [28, 29, 30, 31, 32, 33, 34, 35, 36]. Similarly, in aggressive prostate and ovarian tumors, elevated levels of triglycerides, cholesterol esters, and phospholipids reflect enhanced LD accumulation, reinforcing their potential utility as prognostic markers of tumor aggressiveness and progression [37, 38].

In addition to their prognostic relevance, LDs have also demonstrated diagnostic potential across multiple malignancies [21, 39, 40]. In gastrointestinal cancers, LD enrichment has been associated with the white opaque substance (WOS) observed during endoscopy, linking LDs to clinically observable tumor phenotypes [41]. Despite these insights, LDs remain an underexplored, yet promising source of cancer biomarkers.

Numerous molecular signatures have been developed for both prognostic and diagnostic applications in EC. Prognostic models have incorporated features such as general metabolic reprogramming [42, 43], fatty acid metabolism [44], DNA methylation [45], glycolysis [46], autophagy [47], and ferroptosis‐related pathways associated with immune regulation [48]. Fewer studies have reported diagnostic signatures in EC, including an immune‐related gene panel for microsatellite‐stable tumors [49], a five‐protein signature derived from cervico‐vaginal fluid [50], and circulating microRNA‐based classifiers [51], each demonstrating promising discriminatory performance. While these efforts have expanded our understanding of EC biology and improved molecular classification, they do not address the contribution of LD dynamics to cancer progression or biomarker development. Across multiple cancer types, LD accumulation and LDAG‐based signatures have shown both diagnostic and prognostic value, underscoring their potential as clinically relevant biomarkers [21, 52, 53, 54, 55]. However, their significance in EC remains unexplored.

This study aims to develop and validate LDAG‐based signatures with diagnostic and prognostic relevance in EC. By employing LD‐associated transcriptional profiles, the study proposes a novel, metabolism‐informed approach to improve molecular classification and elucidate associations with clinicopathological features.

## 2 Materials and Methods

### 2.1 Study Design

**FIGURE 1:**
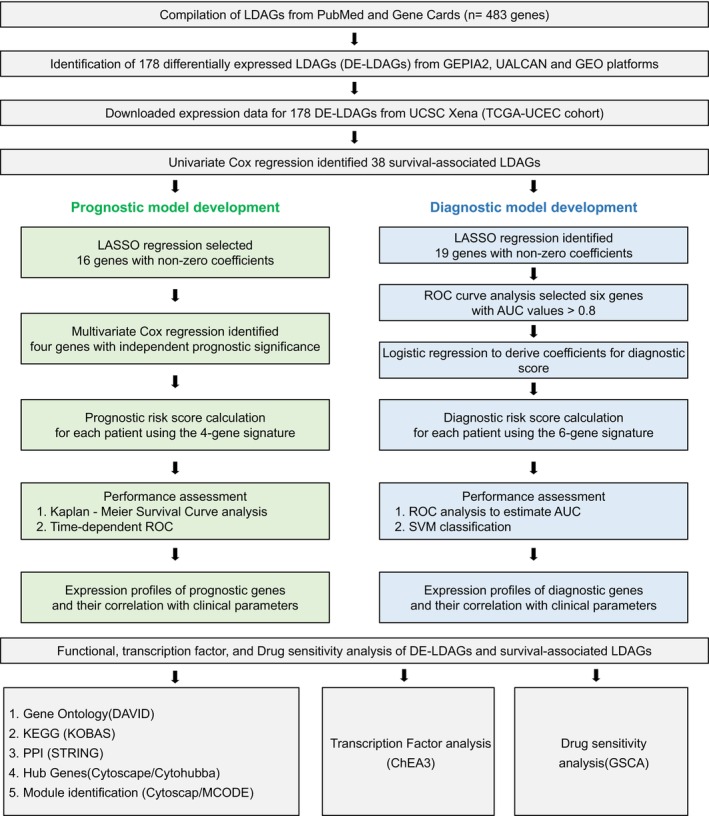
Study design workflow. The figure outlines the bioinformatics analyses used to construct prognostic and diagnostic models from survival‐associated differentially expressed lipid droplet–associated genes (DE‐LDAGs) in endometrial cancer (EC).

This study aimed to develop LDAG signatures with prognostic and diagnostic relevance in EC using an integrated bioinformatics strategy. The analysis workflow included: (1) compiling LDAGs through literature mining and database search; (2) identifying differentially expressed LDAGs (DE‐LDAGs) from publicly available EC datasets; and (3) constructing and validating prognostic and diagnostic models using Cox, LASSO, and logistic regression analyses. Additional validation included expression profiling, functional enrichment, protein–protein interaction (PPI) network analysis, and drug sensitivity correlation (Figure 1).

### 2.2 Identification of Lipid Droplet‐Associated Genes (LDAGs)

LDs are multifunctional organelles involved in energy metabolism, signaling, inflammation, and cancer biology. We selected genes involved in LD biogenesis, structural organization, and related cellular functions by searching PubMed (https://pubmed.ncbi.nlm.nih.gov/) and GeneCards [56] (https://www.genecards.org/) using the terms “lipid droplets,” “lipid droplet‐associated genes,” and “lipid droplets and cancer.” No date restrictions were applied in PubMed [17, 18, 19, 20, 21, 22, 23, 24, 25, 26, 27, 28, 29, 30, 31, 32, 33, 34, 35, 36, 37, 38, 39, 40, 41] and filtered GeneCards entries by a relevance score above 8 to identify genes with strong links to LD functions [53].

### 2.3 Identification of Differentially Expressed‐LDAGs

To identify differentially expressed LDAGs (DE‐LDAGs) in EC, we analyzed publicly available transcriptomic datasets using GEPIA2 (https://gepia2.cancer‐pku.cn) [57], UALCAN (https://ualcan.path.uab.edu) [58], and the Gene Expression Omnibus (GEO; https://www.ncbi.nlm.nih.gov/geo) [59, 60]. Datasets were selected based on relevance to EC, inclusion of matched normal tissue controls, and sufficient sample size to ensure statistical reliability. Differentially expressed genes (DEGs) were identified using a false discovery rate (FDR) threshold of < 0.05 and a fold change (FC) > 2 (log_2_FC > 1 or < −1). Details of the included datasets are provided in Table 1.

**TABLE 1:** Detailed information of the EC datasets.

Database	Dataset ID	EC samples	Normal samples	Upregulated DE‐LDAGs	Downregulated DE‐LDAGs
GEPIA2	UCEC	174	78	41	75
UALCAN	UCEC	19	34	6	4
GEO	GSE17025	91	12	66	30
GEO	GSE63678	7	5	9	2
GEO	GSE115810	24	3	Adjusted *p*‐value non‐insignificant	
GEO	GSE106191	28	29	Adjusted *p*‐value non‐insignificant	

*Note:* Significantly differential genes were defined as those with an adjusted *p*‐value < 0.05 and a fold change greater than 2, i.e., log_2_ (fold change) > 1 or < −1 for up‐ and down‐regulated genes, respectively.

Abbreviations: DE‐LDAGs, differentially expressed lipid droplet associated genes; EC, endometrial cancer; GEO, Gene Expression Omnibus; GEPIA2, gene expression profiling interactive analysis; UALCAN: University of Alabama at Birmingham Cancer data analysis Portal; UCEC, Uterine Corpus Endometrial Carcinoma.

GEPIA2 compares RNA‐seq data from TCGA tumor and GTEx normal tissues, normalized to transcripts per million (TPM). Log_2_ fold changes were computed using the limma package on log‐transformed TPM values. In UALCAN, DE‐LDAGs were selected from the top 250 upregulated and top 250 downregulated genes in the TCGA‐UCEC dataset, based on tumor‐to‐normal median TPM ratios with *p*‐values < 0.01. DEGs from GEO datasets (GSE17025 [61, 62], GSE63678 [63], GSE115810 [64], GSE106191 [65]) were identified using the GEO2R tool (https://www.ncbi.nlm.nih.gov/geo/geo2r/). For microarray datasets (GSE17025, GSE63678), differential expression analysis was performed using limma on normalized, log_2_‐transformed data. For RNA‐seq datasets (GSE115810, GSE106191), DESeq2 was used on normalized counts to compute log_2_ fold changes. In all analyses, FDR < 0.05 was used for significance, applying the Benjamini–Hochberg correction.

Overlapping DE‐LDAGs across datasets were visualized using the Multiple List Comparator tool (https://molbiotools.com/listcompare.php).

### 2.4 Data Sources

Transcriptomic and clinical data for the Uterine Corpus Endometrial Carcinoma (UCEC) cohort were obtained from The Cancer Genome Atlas (TCGA) via the UCSC Xena platform (https://xenabrowser.net/) [66]. The dataset included normalized gene expression values [log_2_(FPKM‐UQ + 1)] for 554 primary tumor samples and 52 normal tissue samples. Samples with missing survival data were excluded, along with those having follow‐up durations under 30 days, to reduce bias from incomplete records and early mortality. This resulted in a final set of 526 tumor samples. These were randomly divided into a training set (*n* = 419) and a testing set (*n* = 107) using an 80:20 split, a standard practice for predictive model development. Clinical characteristics, including age, tumor stage, grade, and body mass index (BMI), were similar between the two sets, minimizing their confounding effects on survival analysis and model performance (Table 2). The training set was used to identify prognostic LDAGs and to develop both prognostic and diagnostic models, while the testing and total sets (*n* = 526) were used for validation.

**TABLE 2:** Clinicopathological characteristics of EC patients in the training and testing sets.

	Training set	Testing set	Total set
Number	419	107	526
Age (years)	63	65	63
BMI (kg/m^2^)	32	33	32
Tumor invasion (%)	47	31	44
Stage І, *N* (%)	259 (62)	68 (64)	327 (62)
Stage II, *N* (%)	41 (10)	10 (9)	51 (10)
Stage III, *N* (%)	97 (23)	24 (22)	121 (23)
Stage IV, *N* (%)	22 (5)	5 (5)	27 (5)
Grade 1, *N* (%)	72 (17)	23 (21)	95 (18)
Grade 2, *N* (%)	91 (22)	25 (23)	116 (22)
Grade 3, *N* (%)	256 (61)	59 (55)	304 (60)

### 2.5 Construction of the LDAG‐Based Prognostic Model for EC

A prognostic model based on LDAGs was constructed using a multistep statistical approach in the TCGA‐UCEC training dataset (*n* = 419). Univariate Cox regression analysis was first conducted in SPSS (IBM Corp., USA) to identify DE‐LDAGs significantly associated with overall survival (*p* < 0.05). These genes were referred to as survival‐associated DE‐LDAGs and selected for model development. To address high dimensionality and multicollinearity in the gene data and enhance model generalizability, Least Absolute Shrinkage and Selection Operator (LASSO) Cox regression was applied using the glmnet package in R (https://cran.r‐project.org/web/packages/glmnet/index.html) with 10‐fold cross‐validation [67, 68, 69]. This method performs variable selection and regularization, identifying the most predictive genes by shrinking less important coefficients to zero while mitigating overfitting. Genes with nonzero coefficients were retained and further subjected to multivariate Cox regression analysis in SPSS to identify independent prognostic genes (*p* < 0.05) and to obtain adjusted coefficient estimates for risk score calculation. A risk score was calculated for each patient using the formula:

$$\text{Risk score}=\sum \left(\beta ᵢ\times \text{expressionᵢ}\right)$$

where *β*ᵢ represents the regression coefficient and expressionᵢ is the normalized gene expression value [log₂(FPKM‐UQ + 1)]. Based on the median risk score calculated in the training set, patients were stratified into high‐ and low‐risk groups. This cutoff value was applied consistently across all datasets.

### 2.6 Prognostic Model Evaluation and Clinical Independence

The prognostic model was evaluated by comparing overall survival between high‐ and low‐risk groups in the training, testing, and total datasets using Kaplan–Meier analysis with log‐rank tests (SPSS, IBM Corp., USA). The prognostic accuracy was further evaluated using time‐dependent ROC curve analysis with the “survivalROC” package in R (https://cran.r‐project.org/web/packages/survivalROC/index.html) [70]. Area under the curve (AUC)values were calculated at 1, 2, 3, and 5 years to assess the model's time‐specific discriminatory performance.

To assess the model's independence from clinical factors, univariate and multivariate Cox regression analyses were performed in SPSS. Hazard ratios and *p*‐values were computed for the risk score and clinical covariates, including age, body mass index (BMI), stage, grade, and invasion status. A heatmap was generated in XLSTAT (Addinsoft) to visualize the distribution of model genes and clinical features between the two risk groups. Additionally, multivariate ROC analysis (SPSS, IBM Corp.) was performed to compare the predictive performance of the risk score with individual clinical variables.

### 2.7 Risk Score Associations and Multidimensional Validation

The biological and clinical relevance of the prognostic model was assessed by analyzing the association between risk group and the expression levels of model genes across tumor stage and grade categories using Kruskal–Wallis tests with Dunn's post hoc correction in GraphPad Prism (GraphPad Software, San Diego, CA, USA). Expression differences between high‐ and low‐risk groups were compared using Student's *t*‐tests. Spearman correlation analysis was conducted in SPSS (IBM Corp., Armonk, NY, USA) to assess associations between risk scores, gene expression, and clinical variables including BMI, tumor invasion, and overall survival time.

To validate the molecular and clinical relevance of the prognostic genes, mRNA and protein expression profiles were examined using UALCAN [58] (TCGA and CPTAC [71] modules), and survival associations were evaluated using the Human Protein Atlas (HPA). Heatmaps were generated to visualize gene expression patterns across risk groups and clinical features.

To investigate the therapeutic relevance of the LDAG‐based prognostic signature, drug sensitivity analysis was performed using the GSCA database (http://bioinfo.life.hust.edu.cn/GSCA/#/) [72, 73]. Drug response data were retrieved from the GDSC and CTRP datasets, where Pearson correlation analysis was performed to assess associations between the expression of the entire 4‐gene signature (PRKAA2, RAB10, LMLN, and LMO3) and drug IC50 values. Only compounds with FDR < 0.05 in at least two genes within the signature were considered significant. Drugs with consistent correlation trends across multiple genes and overlapping representation in both datasets were prioritized for interpretation.

Transcription factors potentially regulating the prognostic genes were identified using the ChEA3 tool (https://amp.pharm.mssm.edu/chea3/) [74].

### 2.8 Functional Enrichment and Protein–Protein Interaction (PPI) Network Analysis

To investigate the biological functions and interactions of the survival‐associated DE‐LDAGs, Gene Ontology (GO) and Kyoto Encyclopedia of Genes and Genomes (KEGG) enrichment analyses were performed. GO terms, including biological processes, cellular components, and molecular functions, were analyzed using DAVID (https://david.ncifcrf.gov/) [75, 76]. KEGG pathway enrichment analysis was conducted using KOBAS v3.0 (http://kobas.cbi.pku.edu.cn/) [77, 78]. Enrichment significance was determined using a FDR threshold of < 0.05.

PPI networks for survival‐associated DE‐LDAGs were generated using STRING (https://string‐db.org/) [79] with a confidence score > 0.4. Networks were visualized in Cytoscape v3.4 (https://cytoscape.org/) [80]. Functional modules were identified using the MCODE plugin with a degree cutoff ≥ 2 and *k*‐core ≥ 2 [81]. Key hub genes were ranked using the cytoHubba plugin based on the degree centrality method, which prioritizes nodes with the highest number of direct connections in the network.

### 2.9 Construction and Evaluation of the LDAG‐Based Diagnostic Model

To identify a gene signature capable of distinguishing endometrial cancer (EC) from normal tissue, a diagnostic model was developed using the previously defined survival‐associated DE‐LDAGs as candidate features. LASSO regression (glmnet package in R) was first applied to reduce multicollinearity and select informative genes with nonzero coefficients. To further refine the model and prioritize genes with strong discriminatory power, univariate ROC analysis (SPSS, IBM Corp.) was performed on the LASSO‐selected LDAGs. Genes with individual AUC values greater than 0.80 were retained for model construction. Binary logistic regression analysis (SPSS, IBM Corp.) was then conducted on the selected genes to estimate coefficients for computing the diagnostic score. The diagnostic score for each sample was computed using the formula:

$$\text{Diagnostic Score}=\beta ₀+\sum \left(\mathrm{\beta ᵢ}\times \text{expressionᵢ}\right)$$

where *β*₀ is the model intercept, *β*ᵢ is the logistic regression coefficient for gene *i*, and expressionᵢ is the normalized gene expression value of gene *i* [log₂ (FPKM‐UQ + 1)]. The score was used to evaluate the model's ability to distinguish EC from normal samples.

Diagnostic model performance was assessed using ROC curve analysis in the training, testing, and total datasets. Additionally, support vector machine (SVM) classification (XLSTAT) was used to assess additional performance metrics, including accuracy, precision, recall, F1‐score, specificity, false positive rate (FPR), normalized error rate (NER), Cohen's kappa, and AUC, to confirm the robustness and generalizability of the diagnostic model.

### 2.10 Expression Profiles and Clinical Significance of Diagnostic LDAGs

To assess the clinical relevance of the diagnostic model, diagnostic scores and expression levels of the six LDAGs were compared between EC and normal tissues across FIGO stages, using Kruskal–Wallis tests with Dunn's post hoc correction (GraphPad Prism). Gene expression patterns were further validated using UALCAN, based on TCGA (transcriptomic) and CPTAC (proteomic) modules. Heatmaps were generated to visualize differences in diagnostic scores and gene expression across clinical stages.

## 3 Results

### 3.1 Selection of LDAGs for the Bioinformatics Analysis

Based on literature and database searches, a total of 483 lipid droplet–associated genes (LDAGs) were compiled for downstream analysis as shown in Table S1.

### 3.2 Identification of DE‐LDAGs in EC

**FIGURE 2:**
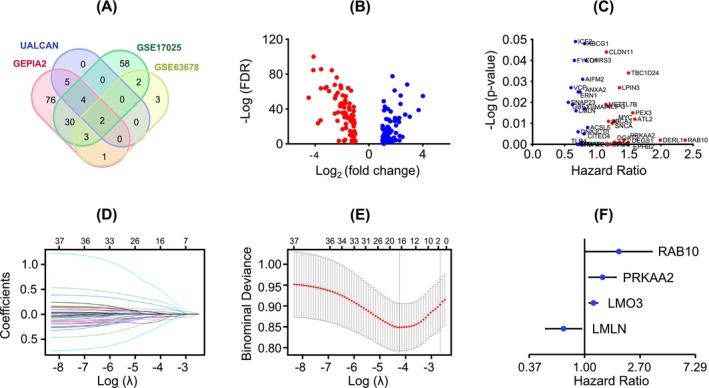
Development and evaluation of a LD–associated prognostic model in EC. (A) Venn diagram of DE‐LDAGs identified from GEPIA2, UALCAN, and GEO (GSE17025, GSE63678) databases. (B) Volcano plots of DE‐LDAGs identified between EC and normal samples in the TCGA‐UCEC training cohort. Blue dots represent up‐regulated genes; red dots represent down‐regulated genes (*p* < 0.05 and log_2_ (fold change) > 1 or < −1 for up‐ and down‐regulated genes, respectively). (C) Volcano plot of univariate Cox regression results, highlighting survival‐associated DE‐LDAGs in the TCGA‐UCEC training cohort. (D) LASSO coefficient profiles of the survival‐associated DE‐LDAGs EC in the TCGA‐UCEC training cohort. (E) A coefficient profile plot was generated against the log(λ) sequence in the LASSO model using the TCGA‐UCEC training cohort. The optimal *λ* value was selected based on 10‐fold cross‐validation and is indicated by the first black dotted line. (F) Forest plot summarizing multivariate Cox regression analysis, identifying four independent prognostic LDAGs in the training cohort.

According to the criteria defined in the Methods, we identified 116 DE‐LDAGs from GEPIA2, 10 from UALCAN, 96 from GSE17025, and 11 from GSE63678, as summarized in Table 1 and shown in Figure S1. After removing duplicates, 178 unique DE‐LDAGs were identified, including 85 upregulated and 93 downregulated genes, as detailed in Table S2. A Venn diagram showing dataset overlaps and a volcano plot displaying the distribution of upregulated and downregulated genes are presented in Figure 2A,B.

### 3.3 Establishment of a LDAG‐Associated Prognostic Model for EC

We developed a LDAG‐associated prognostic model by sequentially applying univariate Cox, LASSO, and multivariate Cox regression analyses to the TCGA‐UCEC training set. Univariate Cox regression first identified 38 survival‐associated DE‐LDAGs significantly associated with overall survival (Figure 2C). To refine this set and reduce overfitting, LASSO Cox regression was applied, selecting 16 candidate genes with nonzero coefficients (Figure 2D,E). Subsequent multivariate Cox regression analysis identified four independent prognostic genes: LMLN, LMO3, PRKAA2, and RAB10. All four genes demonstrated significant associations with overall survival, as shown in the forest plot (Figure 2F). Notably, elevated expression of LMO3, PRKAA2, and RAB10 was associated with worse prognosis, while high LMLN expression predicted better outcomes (Figure 2F). These four genes were used to construct the final prognostic model. Risk scores were calculated for each patient using the risk formula described in the Methods section. The median risk score from the training set was used to classify patients into high‐ and low‐risk groups across all datasets.

### 3.4 Prognostic Model Validation and Comparison With Clinical Variables

**FIGURE 3:**
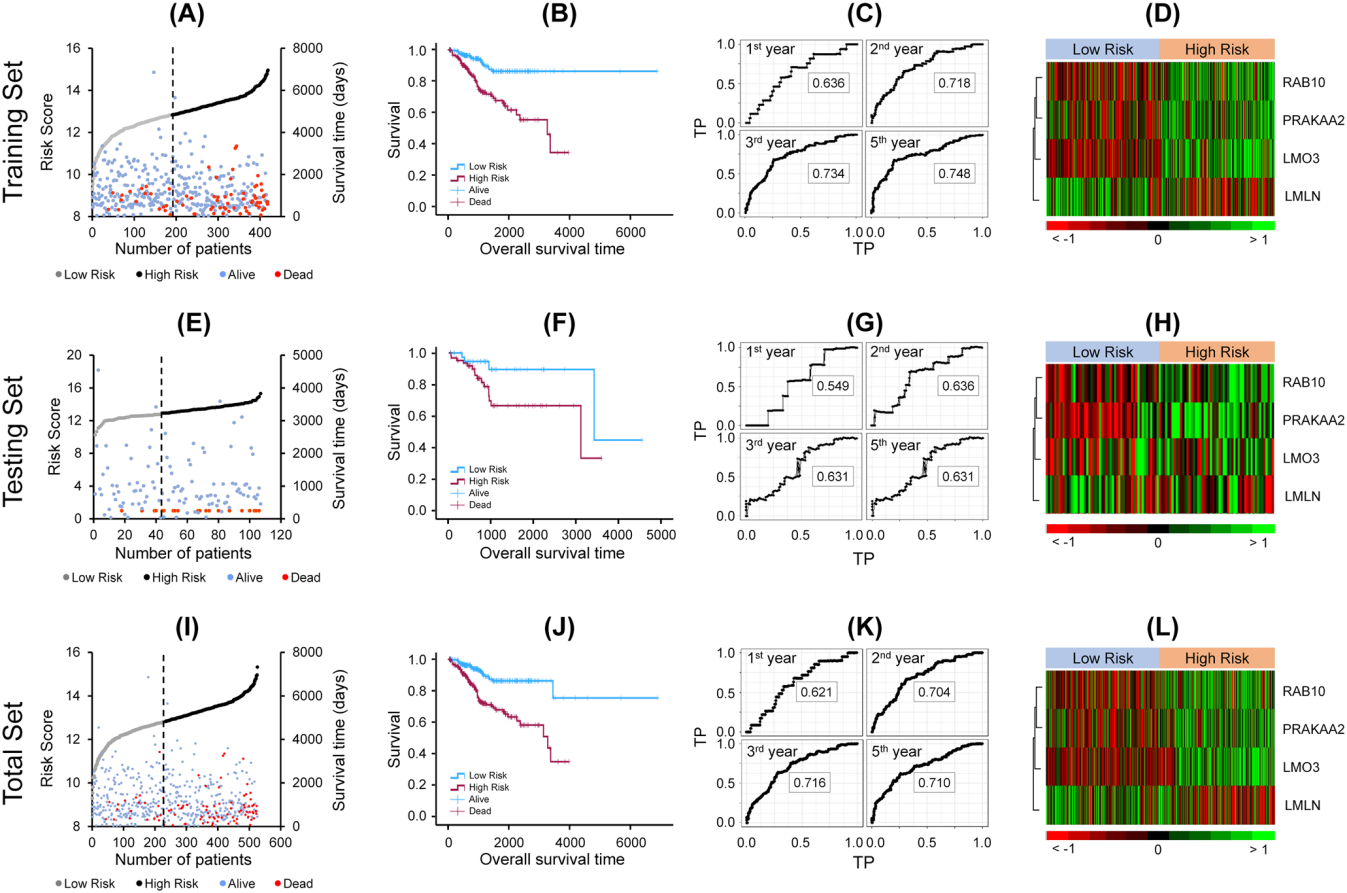
Assessment of prognostic model performance and gene expression profiles. (A, E, I) Distributions of risk scores, overall survival time, and survival status in the training (A), testing (E), and total (I) cohorts, with dotted lines indicating the optimal cut‐off value separating high‐ and low‐risk groups. (B, F, J) Kaplan–Meier survival curves comparing overall survival between high‐ and low‐risk groups in the training (B), testing (F), and total (J) datasets. (C, G, K) Time‐independent ROC curves evaluating the predictive accuracy of the risk score for overall survival in the training (C), testing (G), and total (K) cohorts. (D, H, L) Heatmaps displaying the expression levels of the four prognostic LDAGs in the training (D), testing (H), and total (L) datasets.

We next evaluated the prognostic performance of the LDAG‐based prognostic model and compared it with conventional clinicopathological predictors. In the training cohort (*n* = 419), patients were stratified into high‐risk (*n* = 229) and low‐risk (*n* = 190) groups using the median risk score. Baseline clinical characteristics of the cohorts are summarized in Table 2. The high‐risk group exhibited significantly higher mortality and shorter OS, as shown in the dot plot (Figure 3A) and Kaplan–Meier survival analysis (*p* < 0.0001; Figure 3B). Time‐dependent ROC analysis demonstrated strong model performance, with AUCs of 0.636, 0.718, 0.734, and 0.748 for 1‐, 2‐, 3‐, and 5‐year periods, respectively (Figure 3C). Expression heatmaps revealed upregulation of LMO3, PRKAA2, and RAB10, and downregulation of LMLN in the high‐risk group (Figure 3D).

Model performance was subsequently validated in the testing set (*n* = 107; 64 high‐risk, 43 low‐risk) and the total cohort (*n* = 526; 293 high‐risk, 233 low‐risk). In both sets (Figure 3E–H for testing set, Figure 3I–L for total set), high‐risk patients showed increased mortality and significantly shorter OS (*p* < 0.05), with AUC values ranging from 0.549–0.636 in the testing and 0.621–0.716 in the total set. Gene expression patterns remained consistent across validation sets, supporting the robustness of the model.

**FIGURE 4:**
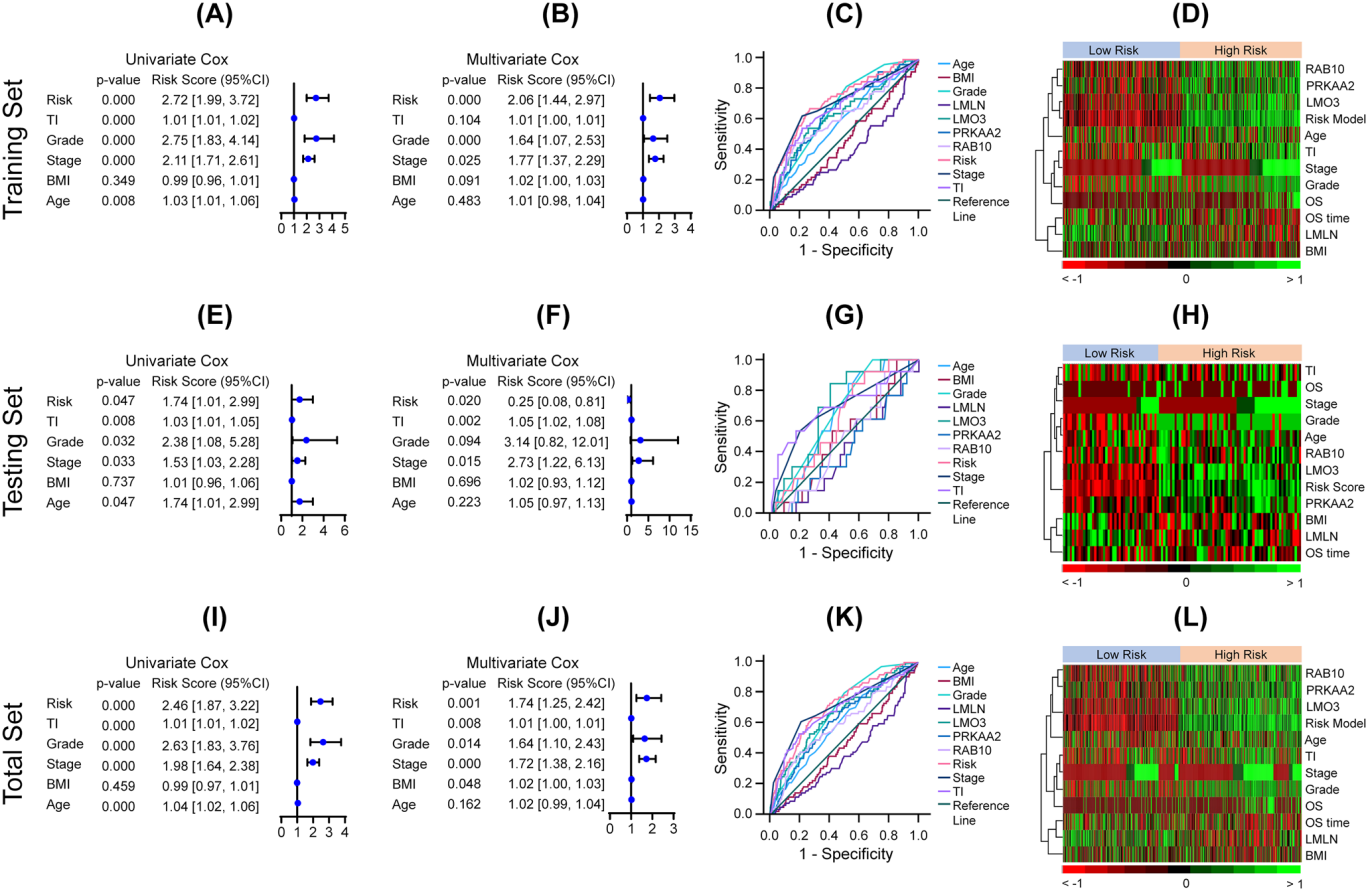
Multivariate assessment of the LDAG risk model and clinical factors. (A, E, I) Forest plots of univariate Cox regression analyses evaluating the prognostic significance of the LDAG‐based risk score and clinical parameters, including tumor invasion percentage (TI) and body mass index (BMI), in the training (A), testing (E), and total (I) cohorts. (B, F, J) Forest plots of multivariate Cox regression analyses identifying the risk score as an independent prognostic factor across the training (B), testing (F), and total (J) cohorts. (C, G, K) time‐dependent ROC curves comparing the predictive performance of the risk score and clinical factors for overall survival in the training (C), testing (G), and total (K) datasets. (D, H, L) Heatmaps showing the distribution of the risk score, expression levels of the four prognostic LDAGs, and associated clinicopathological features, including overall survival status (OS) and survival time (OS time), in the training (D), testing (H), and total (L) cohorts.

To evaluate the prognostic utility of the model, we compared its performance with conventional clinicopathologic indicators. The model demonstrated stronger prognostic performance compared to standard clinicopathologic variables. In the training set, univariate Cox regression identified the LDAG‐based risk model (HR = 2.72, *p* < 0.001), as well as clinical variables including stage, grade, and invasion status, as significant predictors of OS (Figure 4A). Multivariate analysis further confirmed the risk model as an independent prognostic factor (HR = 2.06, *p* < 0.001; Figure 4B). Notably, the four‐gene prognostic model outperformed individual clinical variables, achieving an AUC of 0.736 compared to 0.718 for stage, 0.686 for grade, 0.681 for invasion, and 0.585 for age (Figure 4C). Heatmaps demonstrated clear separation between high‐ and low‐risk groups based on gene expression, risk scores, and clinical features (Figure 4D). Consistent findings were observed in both the testing (Figure 4E–H) and total sets (Figure 4I–L), which showed similarly strong risk stratification, independent prognostic value, and discriminative performance.

### 3.5 Expression Patterns and Clinical Validation of the Prognostic Model

**FIGURE 5:**
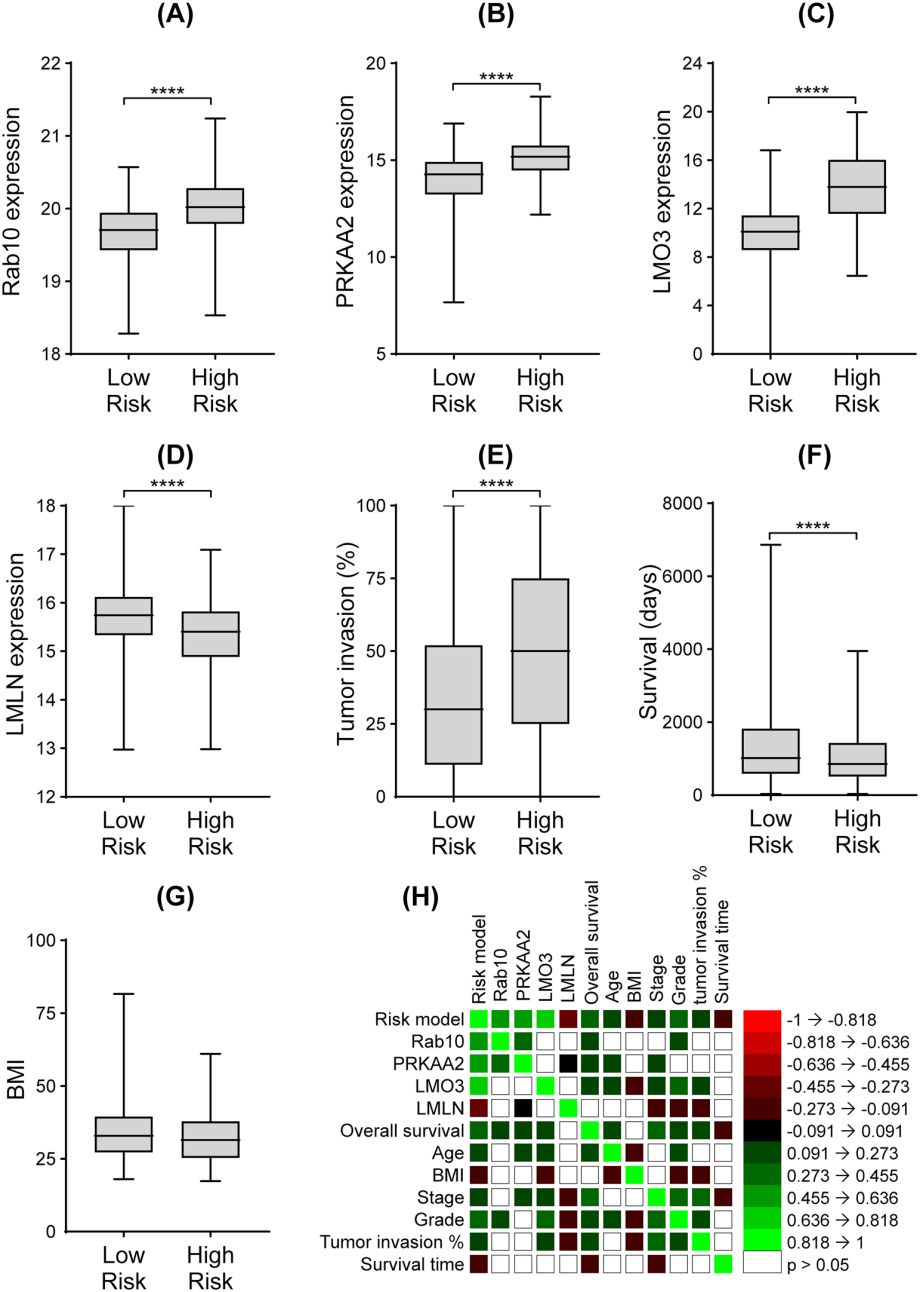
Gene expression profiles and clinical correlation of the risk model. Differences in the expression levels of (A) Rab10, (B) PRKAA2, (C) LMO3, and (D) LMLN, as well as clinical characteristics including tumor invasion percent (E), overall survival time (F), and BMI (G) between the high‐risk and low‐risk categories. Student “*t*” test; *p < 0.05, **p < 0.01, ***p < 0.001, and ****p < 0.0001. (H) Heatmap showing the relationships between the risk score, risk model–associated genes, and clinical characteristics, based on Spearman correlation analysis (*p* < 0.05). Green boxes indicate significant positive correlations, red boxes indicate significant negative correlations, and clear boxes represent non‐significant associations.

The prognostic relevance of individual LDAGs was supported by their expression profiles. *RAB10*, *PRKAA2*, and *LMO3* were significantly upregulated in the high‐risk group, whereas *LMLN* was more highly expressed in the low‐risk group (*p* < 0.0001, Student's *t*‐test; Figure 5A–D). Clinically, high‐risk patients exhibited a greater percentage of tumor invasion (*p* < 0.0001; Figure 5E) and shorter survival time (*p* < 0.01; Figure 5F), with no significant difference in body mass index (BMI) between the two groups (Figure 5G). Spearman correlation analysis supported these associations, showing positive correlations between risk scores and tumor stage, grade, invasion percentage, and expression of RAB10, PRKAA2, and LMO3, and negative correlations with BMI, survival time, and LMLN expression (Figure 5H).

To evaluate the clinical relevance of the prognostic model, we conducted subgroup analyses based on tumor stage and grade, followed by external validation using UALCAN and the Human Protein Atlas. Patients with advanced‐stage or high‐grade tumors exhibited significantly higher risk scores, elevated expression of RAB10, PRKAA2, and LMO3, and reduced LMLN expression compared to those with early‐stage or low‐grade disease (*p* < 0.05, Student's *t*‐test), as demonstrated in Figure S2. UALCAN demonstrated mRNA expression profiles (Figure S3A,D,G,J) and protein expression profiles (Figure S3B,E,H) of RAB10, PRKAA2, LMO3, and LMLN in EC versus normal endometrial tissues. In addition, survival analysis from the Human Protein Atlas supported the prognostic value of these four genes (Figure S3C,F,I,K). Together, these findings demonstrate a strong association between the LDAG‐based prognostic model and conventional clinicopathological risk factors.

### 3.6 Functional Enrichment and PPI Network Analysis

To investigate the biological functions of 38 survival‐associated DE‐LDAGs, we performed GO and KEGG pathway enrichment analyses separately for upregulated and downregulated genes, as shown in Figure S4. GO biological process analysis revealed that upregulated genes were significantly enriched in pathways related to endoplasmic reticulum (ER) function and lipid homeostasis, including the ERAD pathway, cholesterol homeostasis, and cellular response to misfolded protein (Figure S4A). In contrast, downregulated genes were associated with post‐Golgi vesicle‐mediated transport, xenobiotic stimulus response, and ERK1/2 signaling cascade regulation (Figure S4B). GO cellular component analysis indicated that upregulated DE‐LDAGs localized primarily to LDs, endoplasmic reticulum membrane, and the Derlin‐1 retro‐translocation complex (Figure S4C), supporting a role in ER stress resolution and LD formation. In contrast, downregulated genes were associated with mitochondrial, Golgi, and cytoskeletal compartments (Figure S4D), suggesting impairment in energy metabolism and intracellular trafficking. GO molecular function analysis of upregulated genes highlighted enhanced ubiquitin‐specific protease binding and MHC class I protein binding (Figure S4E) whereas downregulated genes showed Hsp70 binding and identical protein binding (Figure S4F).

Although only a subset of enriched terms met FDR significance, the top‐ranked categories (up to 10 per panel) were reported based on nominal *p*‐value thresholds (*p* < 0.05); to capture potentially meaningful biological signals, especially those with known LD‐associated or survival‐related mechanisms.

KEGG pathway analysis of upregulated genes revealed significant enrichment in lipid‐related pathways such as glycerolipid metabolism, sphingolipid metabolism, SNARE interactions in vesicular transport, ABC transporters, cholesterol metabolism, and fat digestion and absorption (Figure S4G). Conversely, downregulated genes were enriched in fatty acid biosynthesis, PI3K‐Akt signaling, and autophagy (Figure S4H), suggesting coordinated regulation of lipid metabolism and stress survival mechanisms.

The PPI network of the 38 survival‐associated DE‐LDAGs, as shown in Figure S5, comprised 38 nodes and 39 edges, with a STRING enrichment *p*‐value of 4.5 × 10^−10^ (Figure S5A). MCODE identified one key cluster containing DGAT2, LIPG, LIPIN3, and PPAP2C (Figure S5B). CytoHubba analysis identified DGAT2, LIPG, MYC, and LIPIN3 as top hub genes with the highest connectivity (Figure S5C). These results highlight a core lipid‐associated regulatory network among survival‐associated DE‐LDAGs.

### 3.7 Drug Sensitivity and Transcriptional Regulation

To explore the therapeutic relevance and regulatory mechanisms underlying the LDAG signature, we next examined drug sensitivity associations and potential upstream transcriptional regulators. Drug sensitivity profiling of the 4‐gene signature (PRKAA2, RAB10, LMLN, LMO3) was performed using the GDSC and CTRP databases, as shown in Figure S6. In the GDSC analysis (Figure S6A), the signature demonstrated selective drug resistance patterns, with elevated expression associated with resistance to kinase inhibitors (CEP‐701, AZD7762) and antimetabolites (methotrexate), among other targeted agents. Negative correlations, indicating increased sensitivity, were observed for the kinase inhibitors lapatinib, AZD8055, and TPCA‐1, the HSP90 inhibitor 17‐AAG, and the microtubule‐targeting agent docetaxel (a taxane). The HDAC inhibitor vorinostat displayed mixed correlations, with positive associations predominating. Overall, the signature was associated with a drug‐resistant phenotype.

In the CTRP analysis (Figure S6B), the signature was likewise associated with positive correlations across a range of compounds, including DNA‐damaging agents (chlorambucil, cytarabine, etoposide), conventional chemotherapeutics (doxorubicin, topotecan, vincristine), and other mechanistically diverse agents. Both HDAC inhibitors (vorinostat and Panobinostat) showed strong positive correlations with the signature. Vorinostat was the only compound to show consistent positive correlations across both datasets.

Furthermore, TF analysis via ChEA3 identified several candidates, including SPI1, YBX3, ZHX1, GTF3A, and CSRNP2, as potential upstream regulators of the prognostic gene signature (LMLN, PRKAA2, LMO3, RAB10), based on co‐expression and ChIP‐seq data as detailed in Table 3. While not all signature genes showed direct TF associations, the results suggest partial but potentially coordinated transcriptional regulation of the gene set.

**TABLE 3:** Drug sensitivity and transcription factor analysis of the prognostic risk model.

Rank	TF	Score	Library	Overlapping genes
1	CSRNP2	63.5	ARCHS4 Coexpression,66; GTEx Coexpression,61	LMLN
2	YBX3	64	ARCHS4 Coexpression,108; GTEx Coexpression,20	PRKAA2
3	ZGPAT	115.3	ARCHS4 Coexpression,110; Enrichr Queries,121; GTEx Coexpression,115	
4	ZHX1	124.3	ARCHS4 Coexpression,159; Enrichr Queries,208; ReMap ChIP‐seq,95; GTEx Coexpression,35	RAB10
5	ANHX	129.5	ARCHS4 Coexpression,120; GTEx Coexpression,139	
6	GTF3A	156	ARCHS4 Coexpression,89; Enrichr Queries,294; GTEx Coexpression,85	PRKAA2
7	SPI1	159.3	Literature ChIP‐seq,33; ARCHS4 Coexpression,453; ENCODE ChIP‐seq,68; Enrichr Queries,73; ReMap ChIP‐seq,109; GTEx Coexpression,220	LMLN
8	POU2F2	159.4	ARCHS4 Coexpression,374; ENCODE ChIP‐seq,89; Enrichr Queries,62; ReMap ChIP‐seq,150; GTEx Coexpression,122	
9	IKZF2	160.3	ARCHS4 Coexpression,134; Enrichr Queries,56; GTEx Coexpression,291	
10	LYL1	160.4	Literature ChIP‐seq,128; ARCHS4 Coexpression,87; Enrichr Queries,36; ReMap ChIP‐seq,167; GTEx Coexpression,384	

### 3.8 Establishment of a LDAG‐Based Diagnostic Model for EC

**FIGURE 6:**
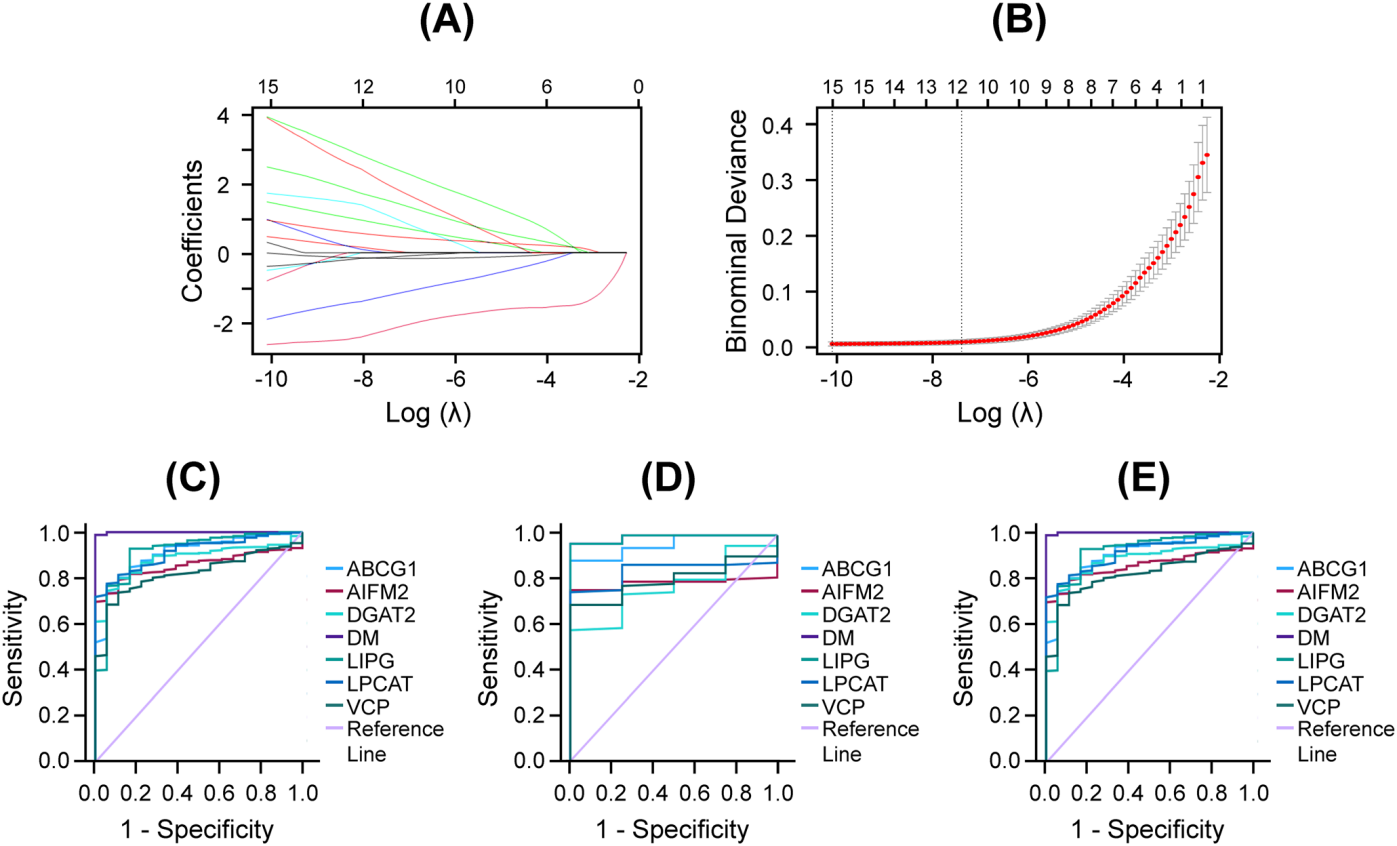
Construction of LDAG‐based diagnostic model. (A) LASSO coefficient profiles of the 38 survival‐associated DE‐LDAGs in the training cohort. (B) Selection of the optimal parameter (lambda) in the LASSO model. A coefficient profile plot was generated against the log (lambda) sequence. (C–E) ROC curve analysis of diagnostic model (DM) and associated genes in the (C) training, (D) testing, and (E) total datasets.

To distinguish endometrial cancer (EC) from normal endometrial tissue, a diagnostic model was developed using the 38 survival‐associated DE‐LDAGs as input features. LASSO regression identified 19 candidate genes with nonzero coefficients (Figure 6A,B). Among these, six genes (ABCG1, AIFM2, DGAT2, LIPG, LPCAT1, and VCP) demonstrated strong discriminatory power (AUC > 0.80) in univariate receiver operating characteristic (ROC) analysis and were selected for diagnostic score construction. Coefficients derived from binary logistic regression, along with gene expression values of the six selected genes, were incorporated into the diagnostic formula to compute a score for each sample, as described in the Methods section. This score was subsequently used to evaluate classification performance across the training, testing, and total datasets.

Across all datasets, the model displayed superior diagnostic performance compared to the individual six LDAGs or other clinical characteristics (Figure 6C–E; Table 4A). This six‐gene diagnostic model demonstrated excellent discriminatory performance with AUC values of 0.999 in the training set (Figure 6C), 1.000 in the testing set (Figure 6D) and 0.999 in the total dataset (Figure 6E). Furthermore, SVM analysis confirmed the model's robust classification performance in the training set (Table 4B), achieving 99.8% accuracy, perfect precision (1.000), and high recall (0.944), resulting in an F‐score of 0.971. The model also exhibited perfect specificity (1.000) and no false positives (FPR = 0.000), with a Cohen's kappa of 0.970 and an AUC of 1.000, indicating strong agreement and discriminatory power. The model demonstrated consistent performance across the testing and total datasets (Table 4B).

**TABLE 4:** Assessment of performance of diagnostic model.

A) ROC analysis—AUC values			
	Training set	Testing set	Total set
Diag. model (DM)	0.999	1.000	0.999
AIFM2	0.857	0.802	0.851
DGAT2	0.872	0.759	0.861
LIPG	0.900	1.000	0.911
LPCAT1	0.920	0.852	0.912
VCP	0.839	0.808	0.833
ABCG1	0.911	0.948	0.917
AGE	0.659	0.834	0.683
BMI	0.419	0.605	0.447
STAGE	1.000	1.000	1.000
GRADE	1.000	1.000	1.000
Tumor invasion (%)	0.571	0.195	0.518

B) SVM classification analysis (AUC values)			
Statistic	Training set	Testing set	Total set
Accuracy	0.998	1.000	0.998
Precision	1.000	1.000	1.000
Recall	0.944	1.000	0.955
F‐score	0.971	1.000	0.977
Specificity	1.000	1.000	1.000
FPR	0.000	0.000	0.000
Prevalence	0.041	0.036	0.040
Cohen's kappa	0.970	1.000	0.976
NER	0.959	0.964	0.960
AUC	1.000	1.000	1.000

### 3.9 Expression Patterns and Clinical Relevance of Diagnostic LDAGs

**FIGURE 7:**
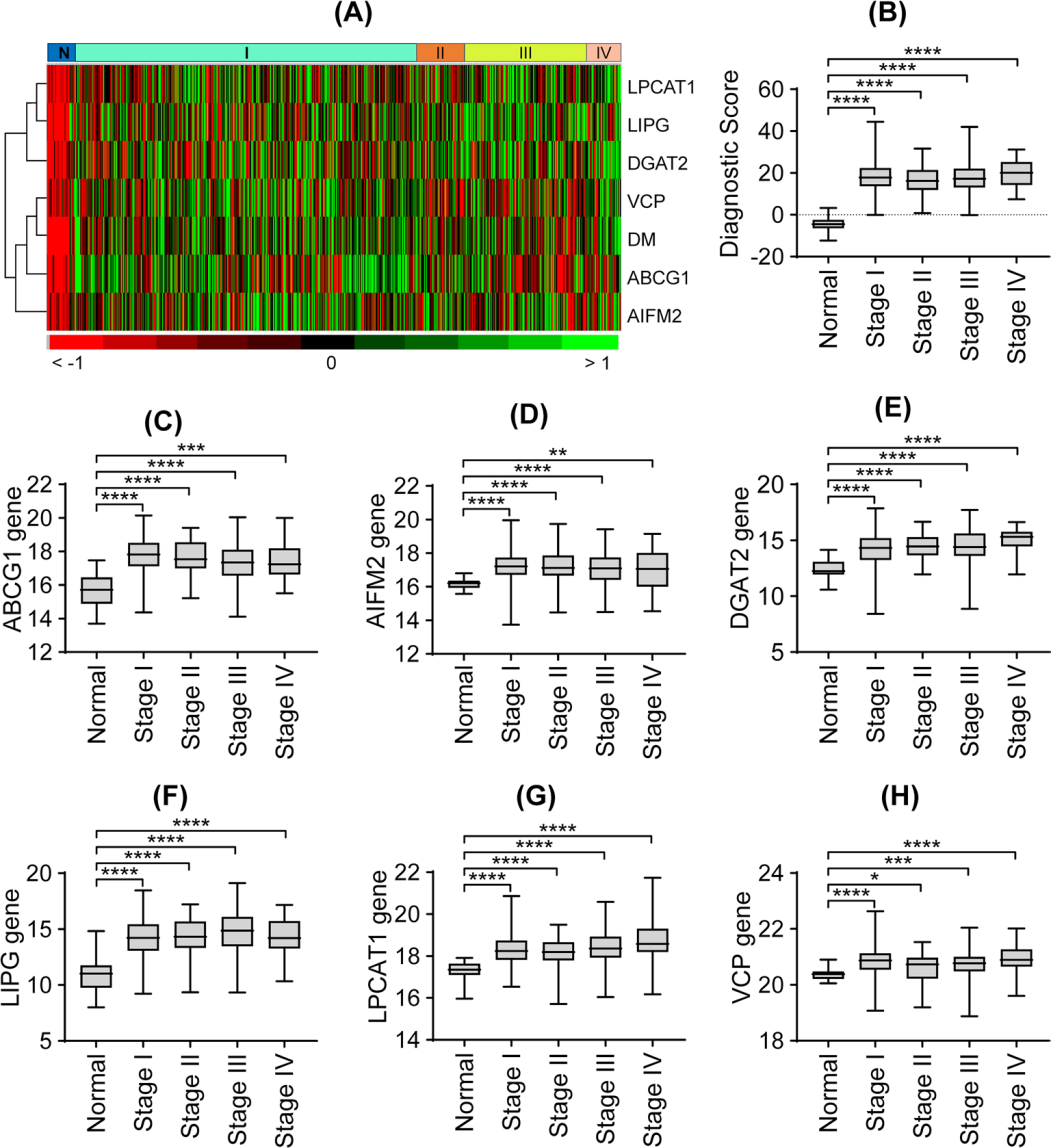
Diagnostic gene expression and risk score across normal and EC samples by clinical stage. (A) Heatmap showing the expression profiles of six diagnostic genes across all groups. (B) Distribution of the diagnostic score (DM). (C–H) Expression levels of individual diagnostic genes: ABCG1 (C), AIFM2 (D), DGAT2 (E), LIPG (F), LPCAT1 (G), and VCP (H). All comparisons were made between normal samples and EC cases stratified by clinical stage. Statistical significance was determined using Student's *t*‐test; *p < 0.05, **p < 0.01, ***p < 0.001, ****p < 0.0001.

We next evaluated the expression patterns and clinical applicability of the diagnostic LDAGs in EC. The heatmap (Figure 7A) showed distinct expression patterns of the six diagnostic LDAGs, with consistently higher expression in EC tumor tissues compared to normal tissues. This upregulation was observed across all FIGO stages (Figure 7B–H), indicating stage‐independent overexpression. Validation using the UALCAN platform confirmed elevated mRNA and protein levels of the six genes in EC samples relative to normal controls as shown in Figure S7, supporting the clinical relevance and robustness of the diagnostic model.

## 4 Discussion

Despite advancements in current prognostic and diagnostic tools for endometrial cancer (EC), key challenges remain, particularly in addressing tumor heterogeneity and achieving diagnostic precision and prognostic stratification. Recent models based on cellular processes such as autophagy, ferroptosis, and metabolic reprogramming have shown predictive value in several malignancies [42, 43, 44, 45, 46, 47, 48, 49, 50, 51, 52, 53, 54]. Emerging evidence linking LD biology to metabolic adaptation in cancer [17, 18, 19, 20, 21, 22, 23, 24, 25, 26, 42, 43, 44, 45, 46, 47, 48, 49] provided the rationale for this study. We conducted the first systematic analysis of lipid droplet–associated gene (LDAG) signatures in EC, designed for both prognostic and diagnostic applications. By integrating transcriptomic data from multiple platforms, we identified differentially expressed LDAGs (DE‐LDAGs) and developed a four‐gene prognostic model and a six‐gene diagnostic model. Both signatures demonstrated clinical relevance for improving disease detection and patient risk stratification.

A four‐gene prognostic model comprising LMLN, LMO3, PRKAA2, and RAB10 effectively stratified EC patients into high‐ and low‐risk groups and demonstrated strong time‐dependent prognostic accuracy, with AUC values exceeding 0.70 across multiple time points. Importantly, the model outperformed conventional clinicopathological factors such as age, stage, and grade, and showed consistent performance across training, testing, and total datasets, supporting its robustness. This LDAG‐based model achieved predictive power comparable to previously reported EC signatures based on glycolysis, fatty acid metabolism, and ferroptosis, which typically report AUC values in the 0.70–0.78 range [42, 43, 44, 45, 46, 47, 48, 49].

To complement the prognostic model, we developed a six‐gene diagnostic model that distinguished EC from normal tissue with near‐perfect accuracy (AUC ≈0.99). The model demonstrated strong classification performance across internal validation sets, with high sensitivity and specificity in SVM‐based classification. Notably, its diagnostic accuracy compares favorably with other published classifiers, including a five‐protein cervico‐vaginal fluid signature (AUC 0.95) [50] and miRNA‐based models (AUCs 0.87–0.93) [51].

Functional annotation of the prognostic model genes supports their mechanistic relevance to tumor biology and clinical outcome. In our study, high expression levels of LMO3, PRKAA2, and RAB10 were significantly associated with unfavorable prognosis in EC patients, underscoring their potential as adverse prognostic indicators. Consistent with our findings in EC, elevated LMO3 expression has been associated with poor prognosis in gastric cancer [82] and neuroblastoma [83], PRKAA2 in endometrial cancer [84, 85] and RAB10 in breast cancer [86] and hepatocellular carcinoma [87]. LMO3 has been identified as an oncogenic driver in gastric cancer [82] and glioma [88], where it promotes invasion and metastasis by suppressing p53‐mediated transcription of apoptotic genes [89]. It also enhances peroxisome proliferator‐activated receptor γ (PPARγ) activity, a master regulator of adipogenesis, and facilitates preadipocyte differentiation [90]. PRKAA2 (AMPKα2), a central energy sensor, inhibits lipid synthesis, promotes lipid breakdown, and regulates lipid droplet turnover by phosphorylating PLIN2, thereby facilitating chaperone‐mediated autophagy [91, 92]. RAB10, a small GTPase, modulates lipid droplet dynamics and mediates autophagic targeting of lipid stores [93, 94]. LMLN, though less extensively characterized, is implicated in cell migration, invasion, and lipid remodeling and is known to localize to lipid droplets in both human and murine cell lines [95, 96]. Collectively, these genes are involved in lipid metabolism, autophagy, and cellular plasticity, which are key processes underlying tumor progression and clinical outcome in EC.

The diagnostic signature also demonstrated strong mechanistic coherence. The six diagnostic genes—AIFM2, ABCG1, LIPG, DGAT2, LPCAT1, and VCP were significantly upregulated in EC and are functionally linked to lipid metabolism, membrane remodeling, redox homeostasis, and cellular stress response. These pathways are activated early in tumorigenesis and may contribute to diagnostic distinction. Notably, ABCG1 has been reported as a diagnostic marker in clear cell renal cell carcinoma [97], and LIPG in gastric cancer [98]. ABCG1 regulates cholesterol transport and membrane composition and has been implicated in the progression of ovarian and prostate cancers [99, 100]. LIPG, a cell surface lipase, has been implicated in tumor proliferation, progression, and metastasis. It is upregulated in response to oxidative stress via AMPK signaling, promoting lipid droplet accumulation and supporting cancer cell survival [101]. While LPCAT1, AIFM2, and VCP have not been directly validated as diagnostic biomarkers, they are functionally implicated in tumor pathogenesis. LPCAT1 contributes to phospholipid remodeling and has been linked to enhanced cellular plasticity in tumors [102, 103]. VCP regulates ER‐associated degradation, autophagy, apoptosis, and lipid droplet biogenesis and is implicated in various malignancies [104]. AIFM2, also known as ferroptosis suppressor protein 1 (FSP1), protects cells from lipid peroxidation and has been associated with therapy resistance in multiple malignancies [105]. While diagnostic roles for DGAT2 have not been widely reported, it plays a key metabolic function in triglyceride synthesis and lipid droplet formation. Our laboratory analyses further confirmed the upregulation of both DGAT1 and DGAT2 in endometrial tumor tissues (unpublished data), supporting their role in lipid accumulation and reinforcing their inclusion in the diagnostic model. The coordinated overexpression of these genes may enhance detection sensitivity by capturing tumor‐associated metabolic reprogramming and support the diagnostic utility of this multi‐gene signature.

The translational potential of our prognostic LDAG signature extends beyond classification, offering insight into therapeutic stratification in EC. Drug sensitivity profiling revealed that high LDAG expression is associated with reduced sensitivity to kinase inhibitors, HDAC inhibitors, and chemotherapeutics, suggesting a protective metabolic phenotype involving enhanced lipid storage and redox buffering, leading to altered metabolism. Conversely, increased sensitivity to select kinase and HSP90 inhibitors in low‐LDAG contexts highlights the potential utility of LDAG expression as a biomarker for treatment selection. Notably, HDACs, frequently overexpressed in EC, are known to regulate lipid metabolism and cellular stress responses, aligning with the lipid and ER stress–related functions enriched in our LDAG model [106, 107]. These findings raise the possibility that HDAC inhibition may restore drug sensitivity in LDAG‐high tumors. KEGG enrichment analysis further revealed that upregulated LDAGs were linked to lipid metabolism, ER stress resolution, and cholesterol homeostasis, while downregulated genes reflected impaired autophagy, mitochondrial dysfunction, and PI3K‐Akt signaling. Together, these features may enable tumor cells to adapt to therapeutic stress, evade apoptosis, and promote resistance. Although such lipid‐driven mechanisms are increasingly recognized in other cancers, they remain underexplored in EC. These findings underscore the clinical relevance of LDAGs as both biomarkers of therapy response and potential targets for metabolic intervention.

In summary, this study demonstrates the dual clinical relevance of LDAGs in EC through the development of both prognostic and diagnostic models derived from a common set of survival‐associated DE‐LDAGs. These signatures reflect critical aspects of metabolic dysregulation, tumor aggressiveness, and progression, and offer a mechanistically informed basis for improved risk stratification and clinical decision‐making.

Despite the strengths of this study, several limitations should be acknowledged. Both prognostic and diagnostic models were developed using RNA sequencing data from the TCGA cohort and require validation in independent patient datasets to ensure broader applicability and clinical relevance. Additionally, experimental confirmation of LDAG expression and functional relevance in EC progression is essential. This includes validation in cell lines and in vivo models, such as xenografts.

To ensure reproducibility and translational relevance, future studies should evaluate these models in clinically diverse cohorts that capture a broader range of histological subtypes and disease stages. Evaluating LDAG expression in blood, serum, or peritoneal fluid may further support their translation into noninvasive diagnostic tools. Recent efforts to identify EC biomarkers from cervico‐vaginal fluid [50] and circulating miRNAs [51] highlight the feasibility of biofluid‐based assays. The six‐gene diagnostic signature, with strong discriminatory power and a compact gene panel, is a promising candidate for development into minimally invasive assays using cervico‐vaginal or blood‐derived RNA. Such assays may be implemented using RT‐qPCR [108], targeted RNA panels, or multiplex platforms [9], (xMAP bead‐based immunoassays), all of which are compatible with routine clinical workflows. Moreover, a multiomics approach that integrates transcriptomic, proteomic, and lipidomic data could provide deeper insights into the tumor microenvironment and enhance the accuracy and clinical applicability of the models. Ultimately, incorporating LDAG signatures into biomarker‐driven clinical trials could help personalize treatment strategies and identify metabolic vulnerabilities amenable to therapeutic targeting.

## 5 Conclusions

This study identifies novel LD‐associated prognostic and diagnostic gene signatures in endometrial cancer that reflect transcriptional changes associated with LD‐mediated metabolic adaptation. These findings highlight a previously underexplored metabolic dimension of EC progression. Further validation in diverse patient cohorts and noninvasive biospecimens is needed to evaluate their utility in early detection, risk stratification, and metabolism‐informed therapeutic strategies.

## Author Contributions

L.B. provided resources and acquired funding for the investigation. V.N.A. and L.B. were the Principal Investigators for the study and participated in its conceptualization and design. V.N.A., M.L., P.D.S., K.G., E.M.S., and T.W. conducted literature review and data collection. V.N.A., M.L., and P.D.S. participated in the formal analysis of data. V.N.A. wrote the original draft. V.N.A. and P.D.S. designed the figures and tables. All authors reviewed, edited, read, and approved the final manuscript.

## Ethics Statement

This study utilized publicly available data and did not involve human participants.

## Consent

Institutional review board approval and informed consent were not required.

## Conflicts of Interest

The authors declare no conflicts of interest.

## Supporting information


**Figure S1:** DE‐LDAGs in EC compared to normal samples. Each heatmap illustrates DE‐LDAGs identified from GEPIA2 (A), GSE17025 (B), GSE63678 (C), and UALCAN (D). Red boxes indicate up‐regulated genes whereas green boxes indicate down‐regulated genes. Adjusted *p*‐value < 0.05 and a fold change greater than 2, i.e., log_2_ (fold change) > 1 or < −1 for up‐ and down‐regulated genes, respectively.

**Figure S2:** Risk score and prognostic gene expression across tumor stages and grades. Box plots show the distribution of the risk score and expression levels of associated prognostic genes across tumor stage and grade categories. Statistical significance was assessed using Student's *t*‐test; *p < 0.05, **p < 0.01, ***p < 0.001. (A, C, E, G, I) Comparisons between early‐stage and advanced‐stage tumors. (B, D, F, H, J) Comparisons between low‐grade and high‐grade tumors.

**Figure S3:** Stagewise expression and survival profiles of risk model genes. (A, D, G, J) mRNA and (B, E, H) protein levels from UALCAN platform. (C, F, I, K) Survival curves from HPA (human protein atlas). Assessed genes were LMO3 (A, B, C), PRKAA2 (D, E, F), Rab10 (G, H, I), and LMLN (J, K). Significance levels indicated as follows: *p < 0.05, **p < 0.01, ***p < 0.001 and ****p < 0.0001.

**Figure S4:** Functional enrichment analysis of survival‐associated DE‐LDAGs in EC. GO term and KEGG pathway enrichment analyses were performed separately for upregulated and downregulated survival‐associated DE‐LDAGs using DAVID and KOBAS. Panels show enriched biological processes (A: upregulated, B: downregulated), cellular components (C: upregulated, D: downregulated), molecular functions (E: upregulated, F: downregulated), and KEGG pathways (G: upregulated, H: downregulated). Bar colors indicate statistical significance: dark blue bars represent FDR < 0.05, lighter blue bars represent *p* < 0.05 but FDR ≥ 0.05, and gray bars indicate *p* > 0.05.

**Figure S5:** PPI network and module analysis of survival‐associated DE‐LDAGs in EC. (A) PPI network constructed using STRING. (B) Key module identified using the MCODE plug‐in in Cytoscape. (C) Top 10 hub genes identified using the cytoHubba plug‐in.

**Figure S6:** Drug sensitivity analysis of 4‐gene prognostic signature. Correlation analysis between the 4‐gene LDAG signature (PRKAA2, RAB10, LMLN, LMO3) and drug response data from the GSCA platform. Panels show the top 30 drugs most significantly associated with the signature in the CTRP (A) and GDSC (B) datasets. Positive correlations indicate that higher signature expression is associated with reduced drug efficacy, whereas negative correlations suggest increased drug sensitivity. In the GSCA bubble plot, color intensity reflects correlation strength (red: positive; blue: negative), bubble size denotes FDR significance, and black outlines indicate FDR ≤ 0.05.

**Figure S7:** Stagewise expression of diagnostic model genes in endometrial cancer. (A, C, E, G, I, K) mRNA expression and (B, D, F, H, J) protein levels of six diagnostic genes in endometrial cancer, based on UALCAN data. Student “*t*” test; *p < 0.05, **p < 0.01, ***p < 0.001 and ****p < 0.0001.

**Table S1:** Compiled list of LDAGs identified from the literature and gene cards
**Table S2:** (B) Final list of the 178 DE‐LDAGs selected for the bioinformatics analyses.

## Data Availability

The data that support the findings of this study are available from the corresponding author upon reasonable request.
