# Supplementary figures and images for: Bioinformatics Analysis Identifies Lipid Droplet‐Associated Gene Signatures as Promising Prognostic and Diagnostic Models for Endometrial Cancer
Source: Cancer Rep (Hoboken). 2025 Aug 13;8(8):e70313. doi: 10.1002/cnr2.70313 (PMC12350079; doi:10.1002/cnr2.70313)

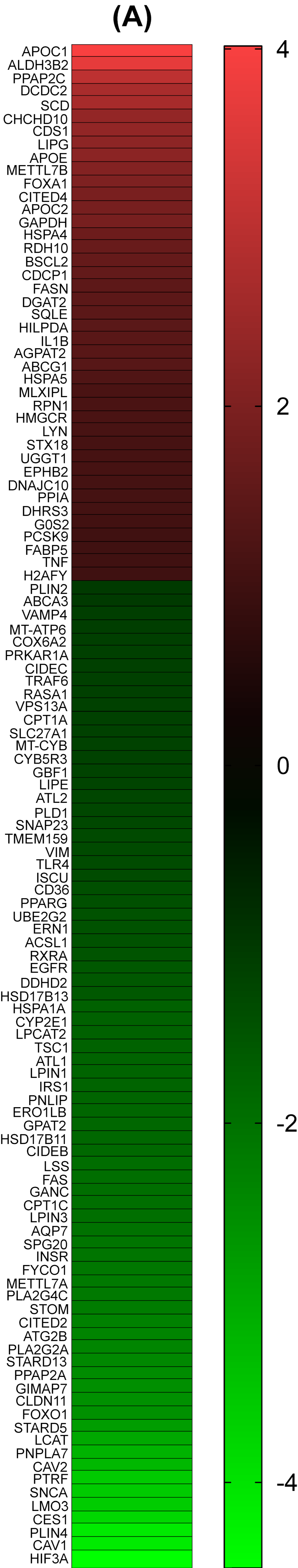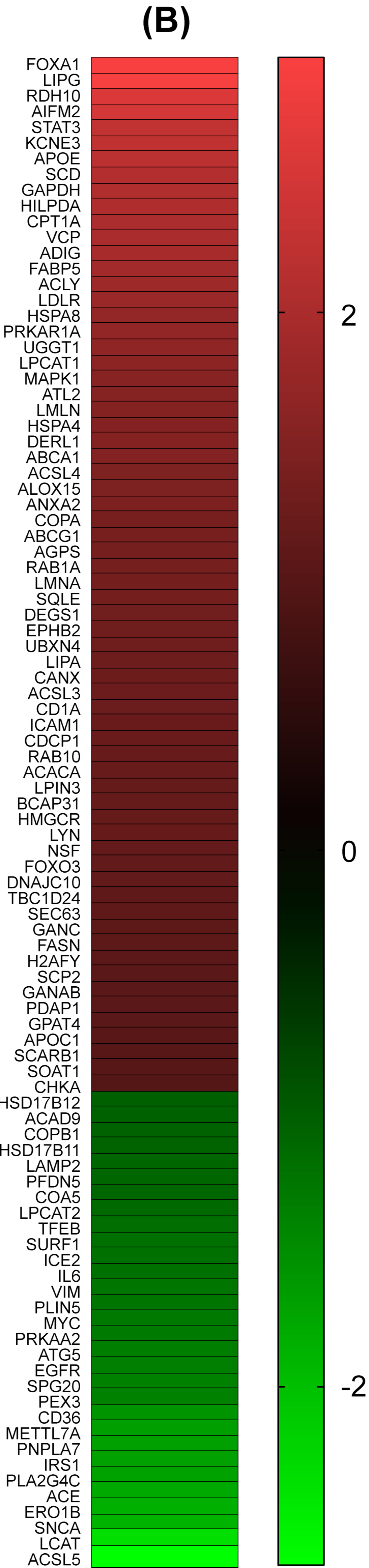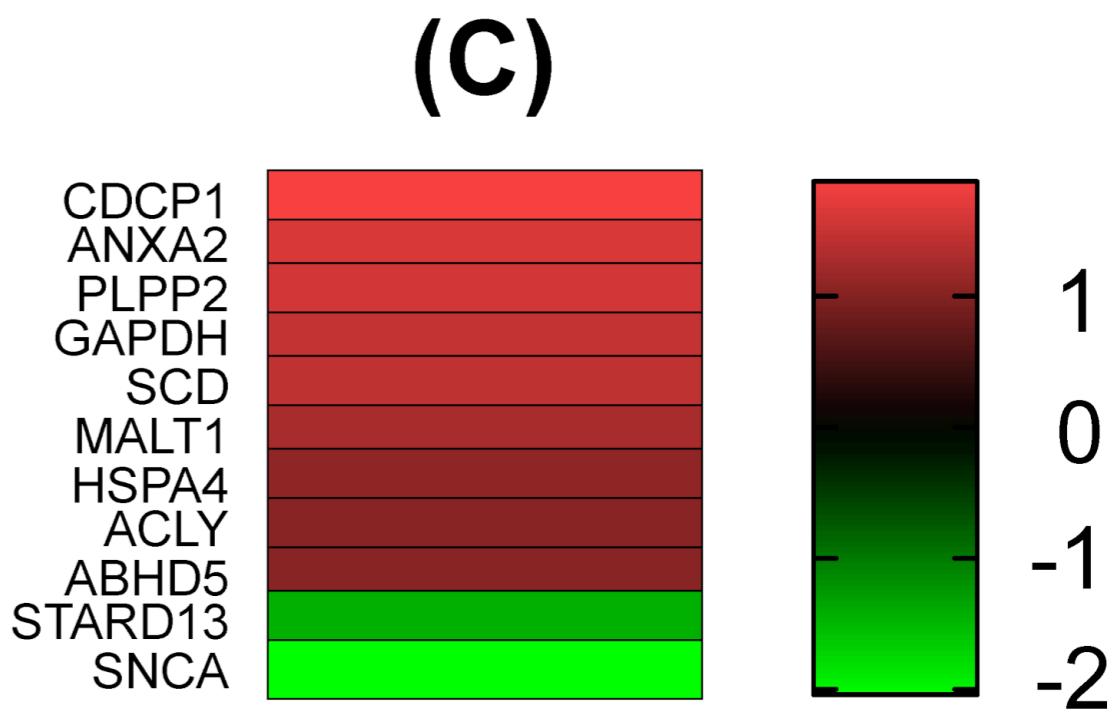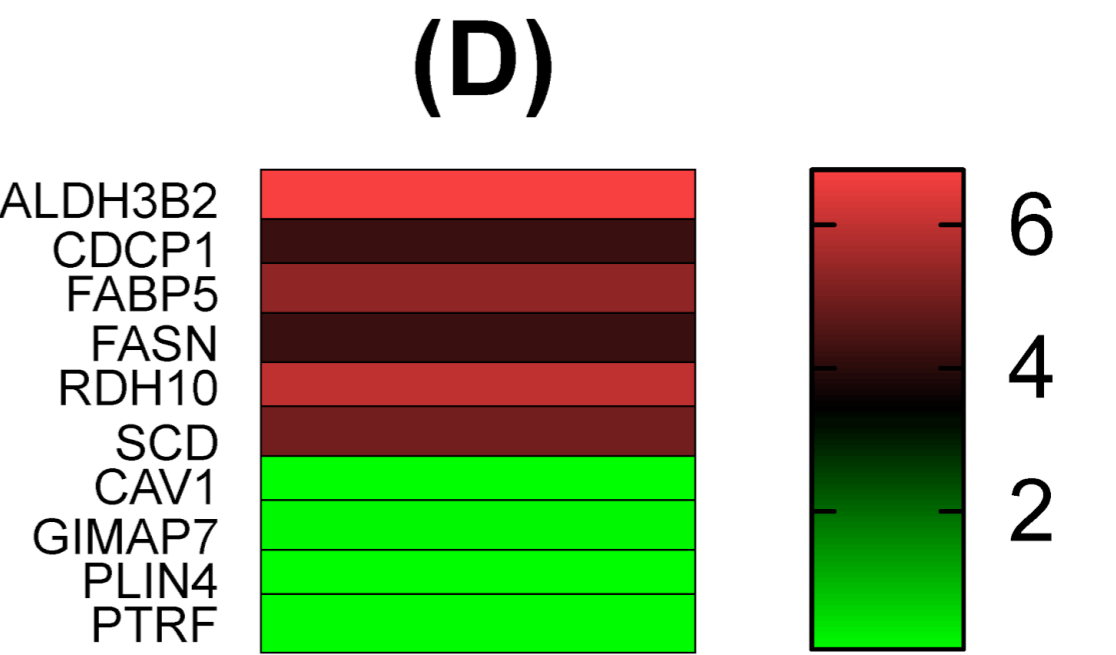

Supplement: Supplementary file 1 — Figure S1: DE‐LDAGs in EC compared to normal samples. Each heatmap illustrates DE‐LDAGs identified from GEPIA2 (A), GSE17025 (B), GSE63678 (C), and UALCAN (D). Red boxes indicate up‐regulated genes whereas green boxes indicate down‐regulated genes. Adjusted p‐value < 0.05 and a fold change greater than 2, i.e., log2 (fold change) > 1 or < −1 for up‐ and down‐regulated genes, respectively. [file CNR2-8-e70313-s003.pdf]

**(A)**

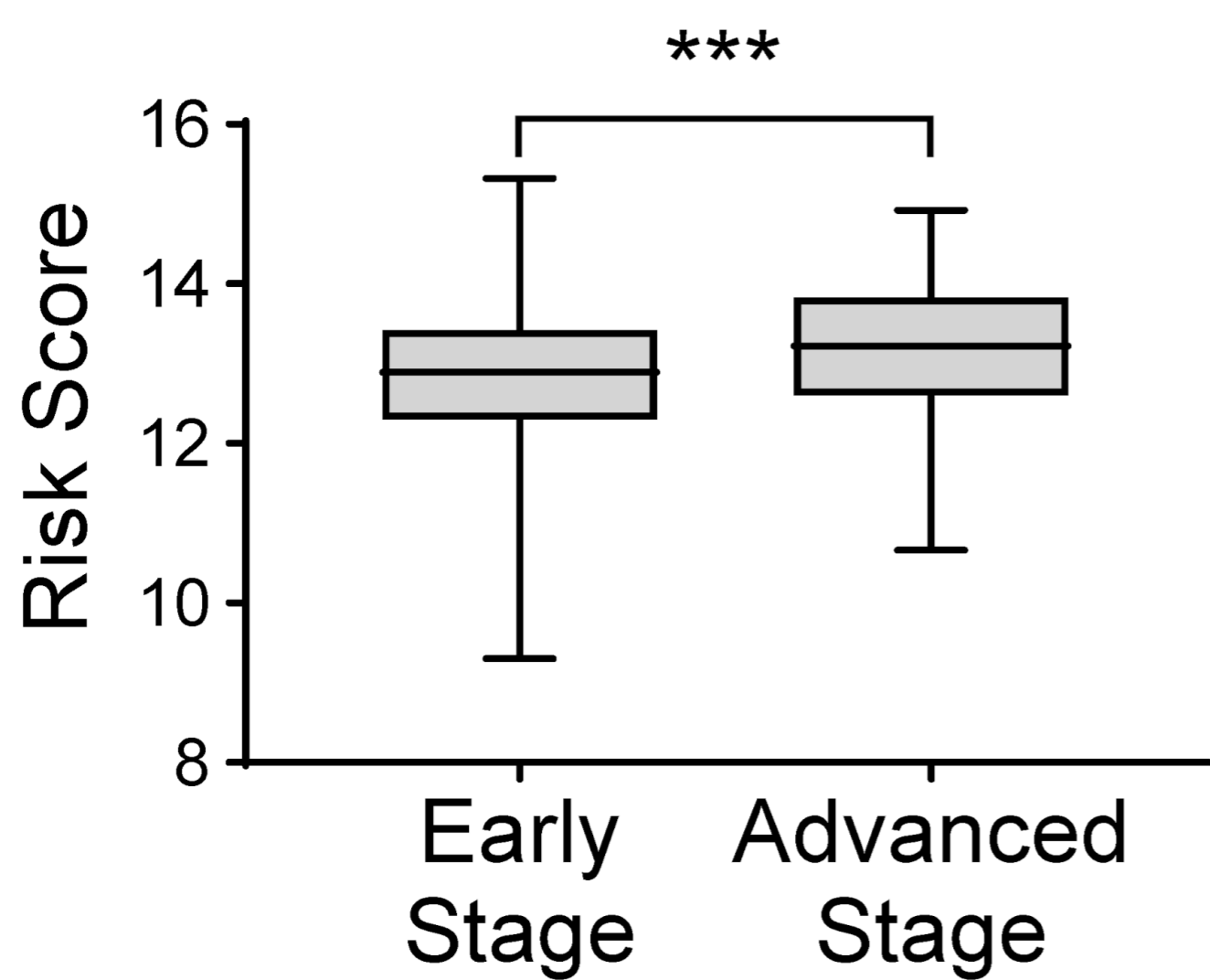

**(B)**

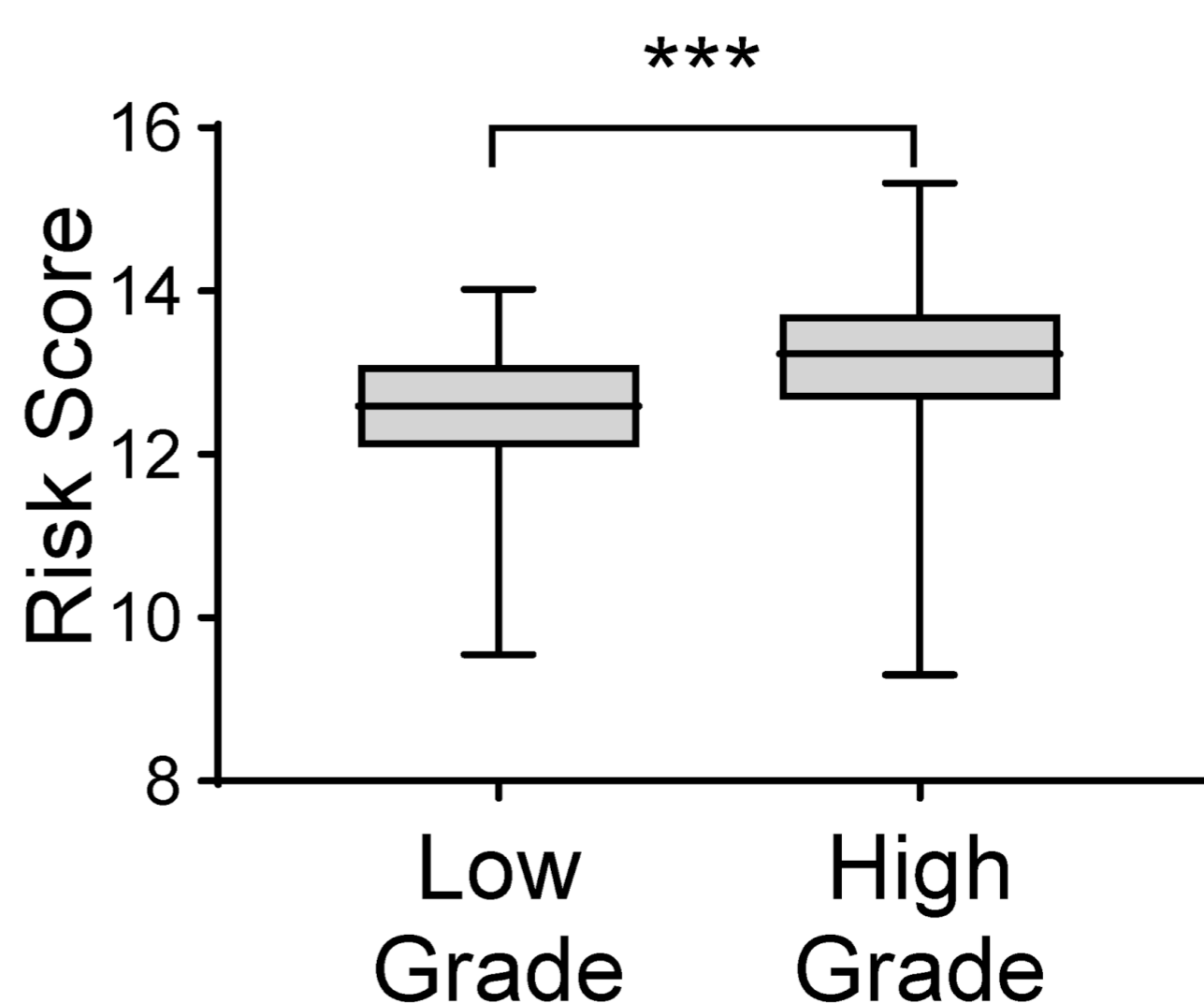

**(C)**

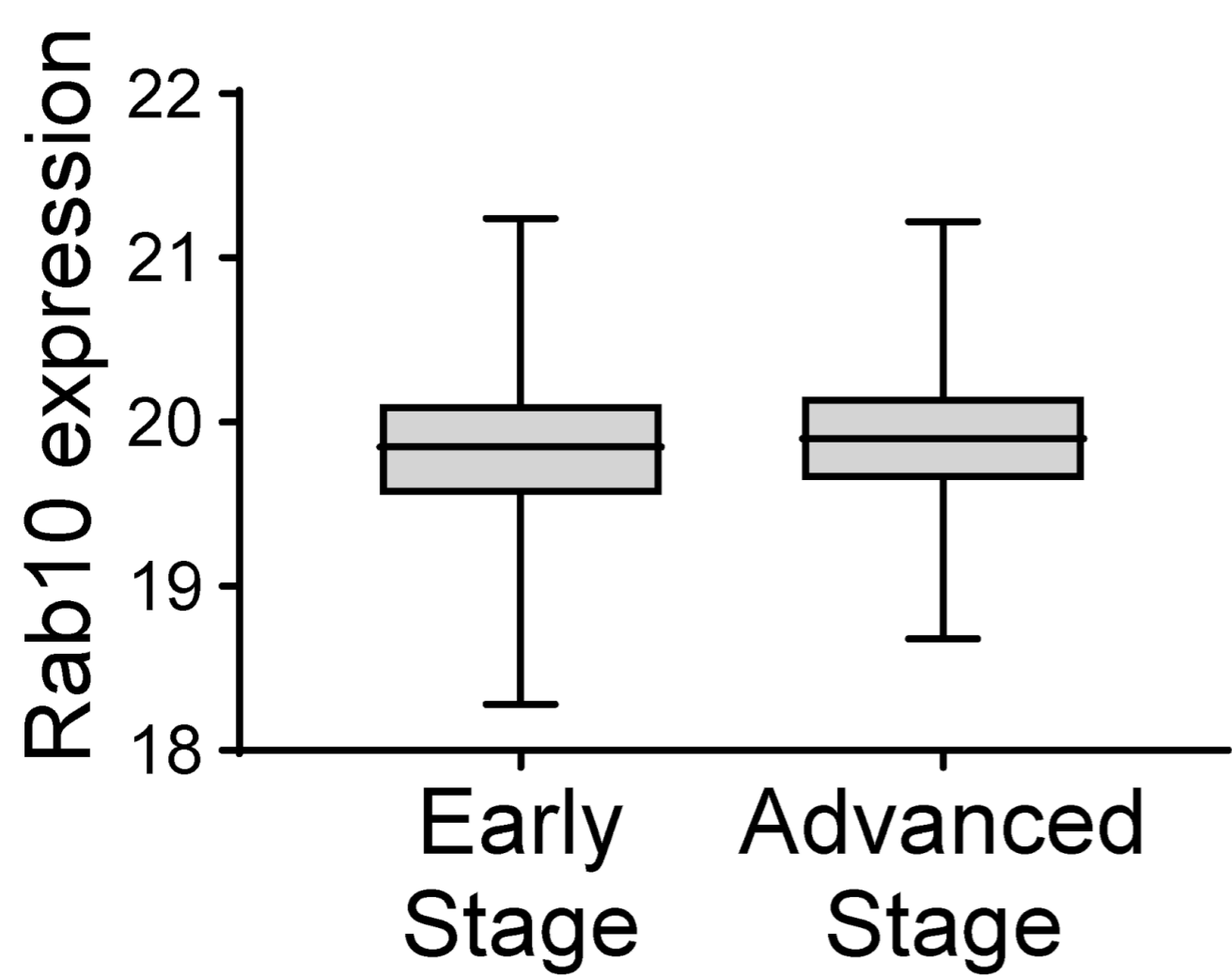

**(D)**

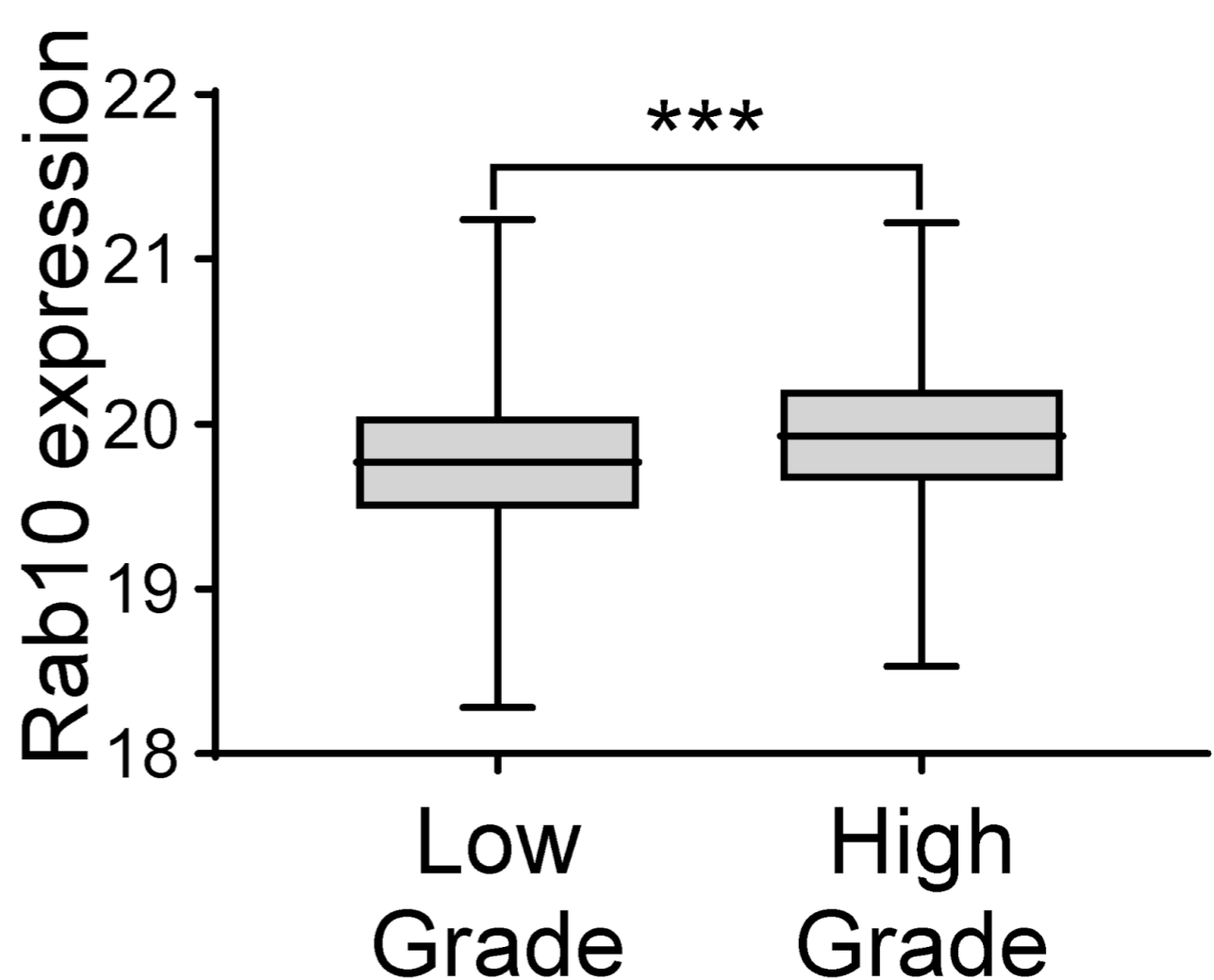

**(E)**

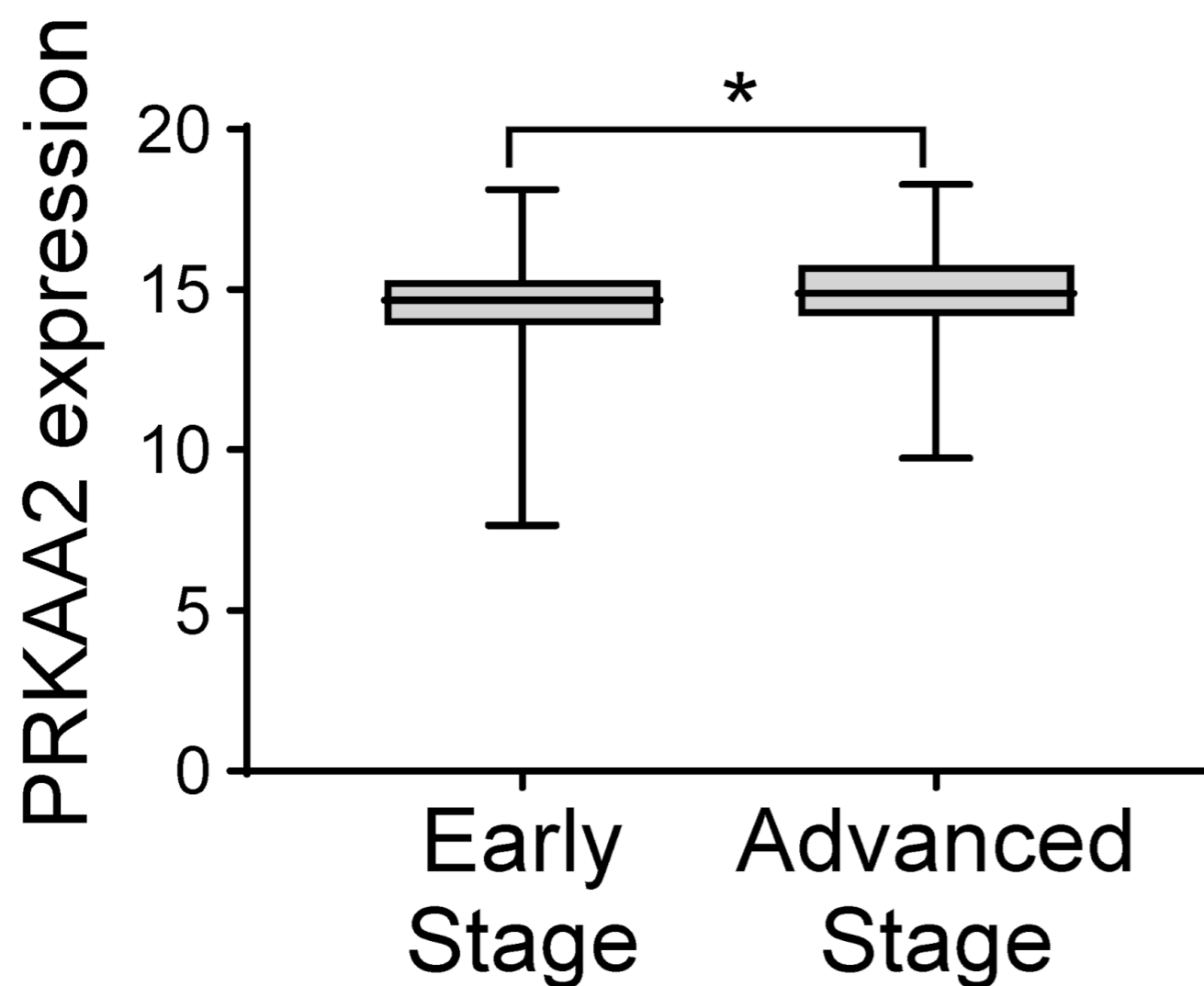

**(F)**

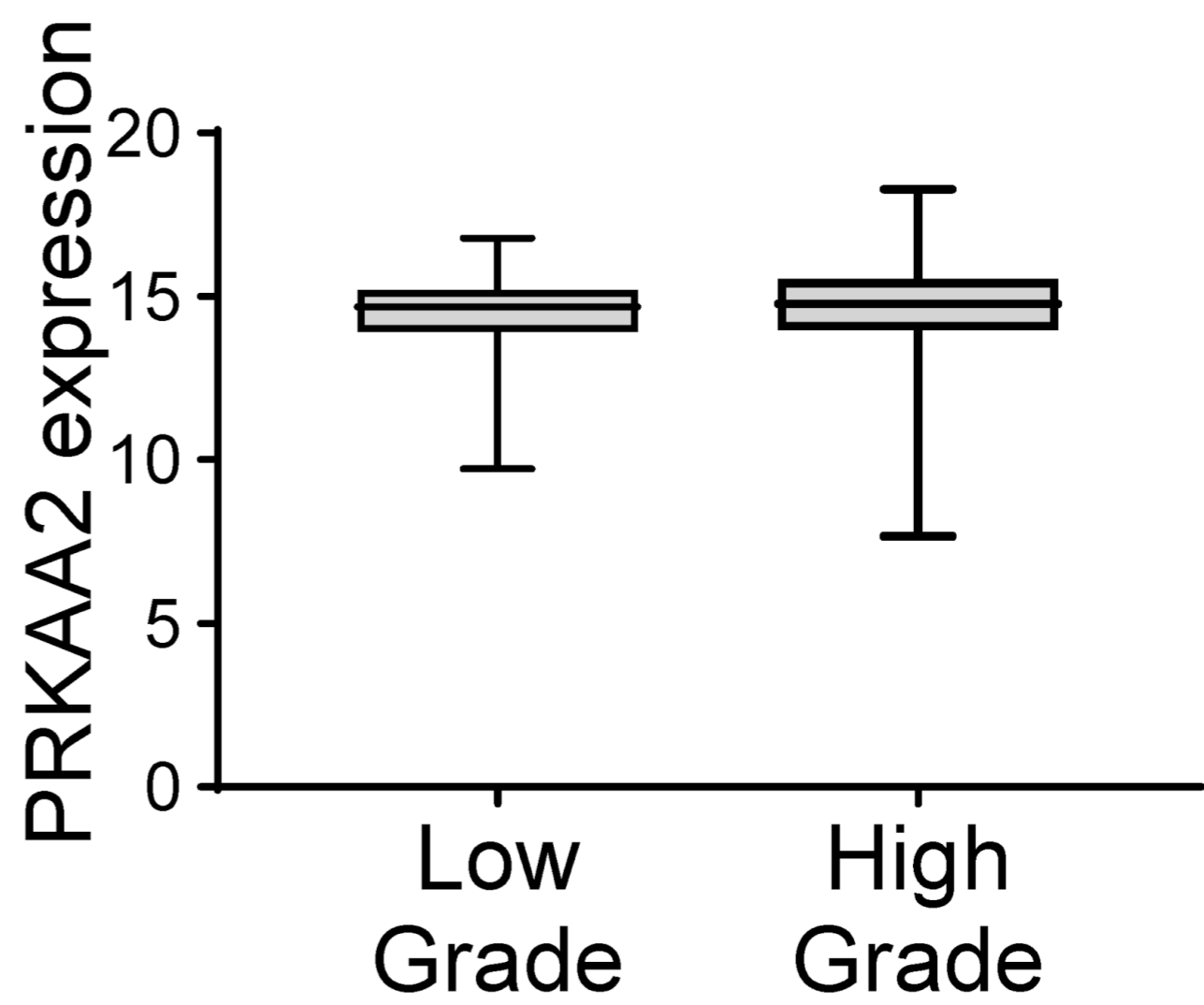

**(G)**

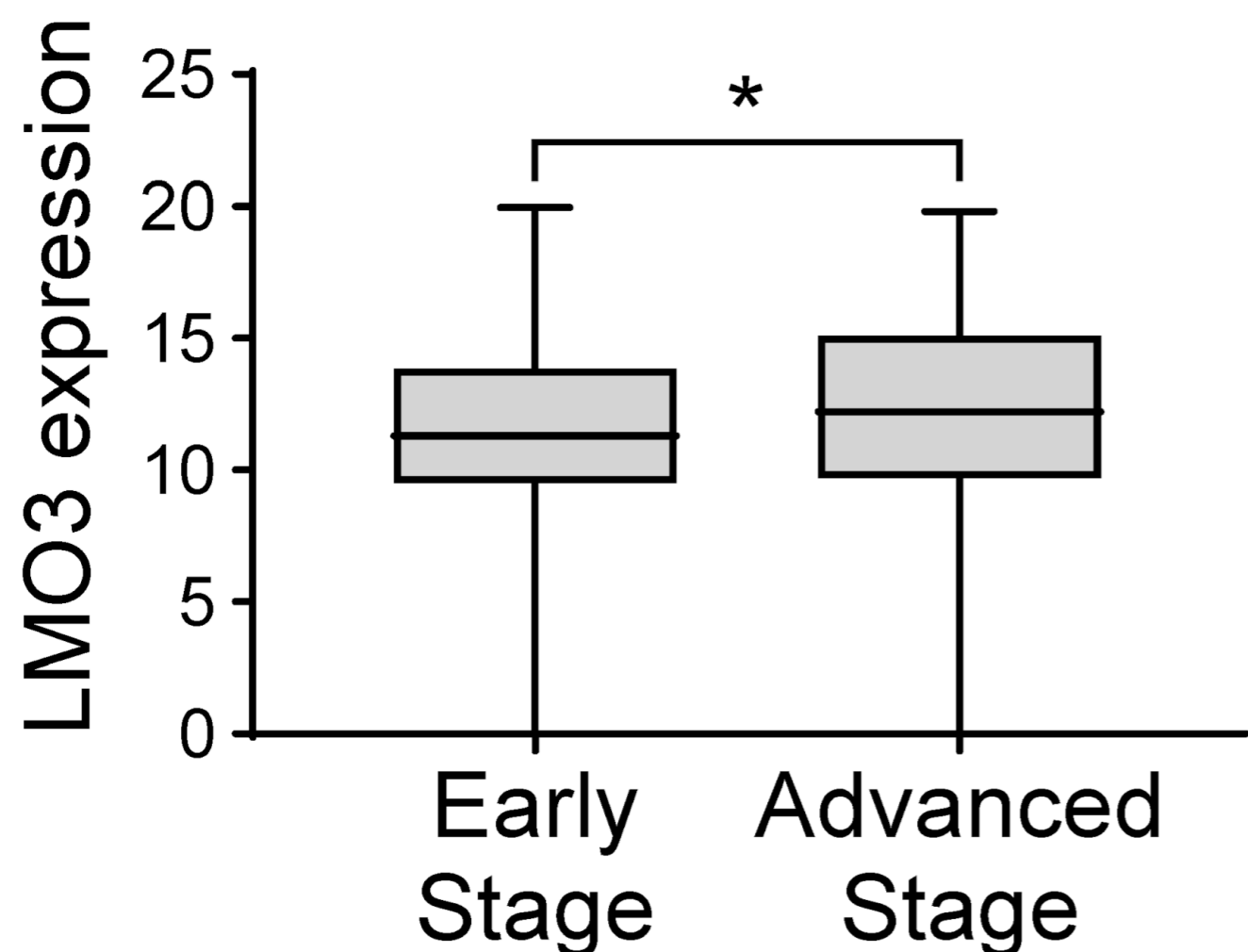

**(H)**

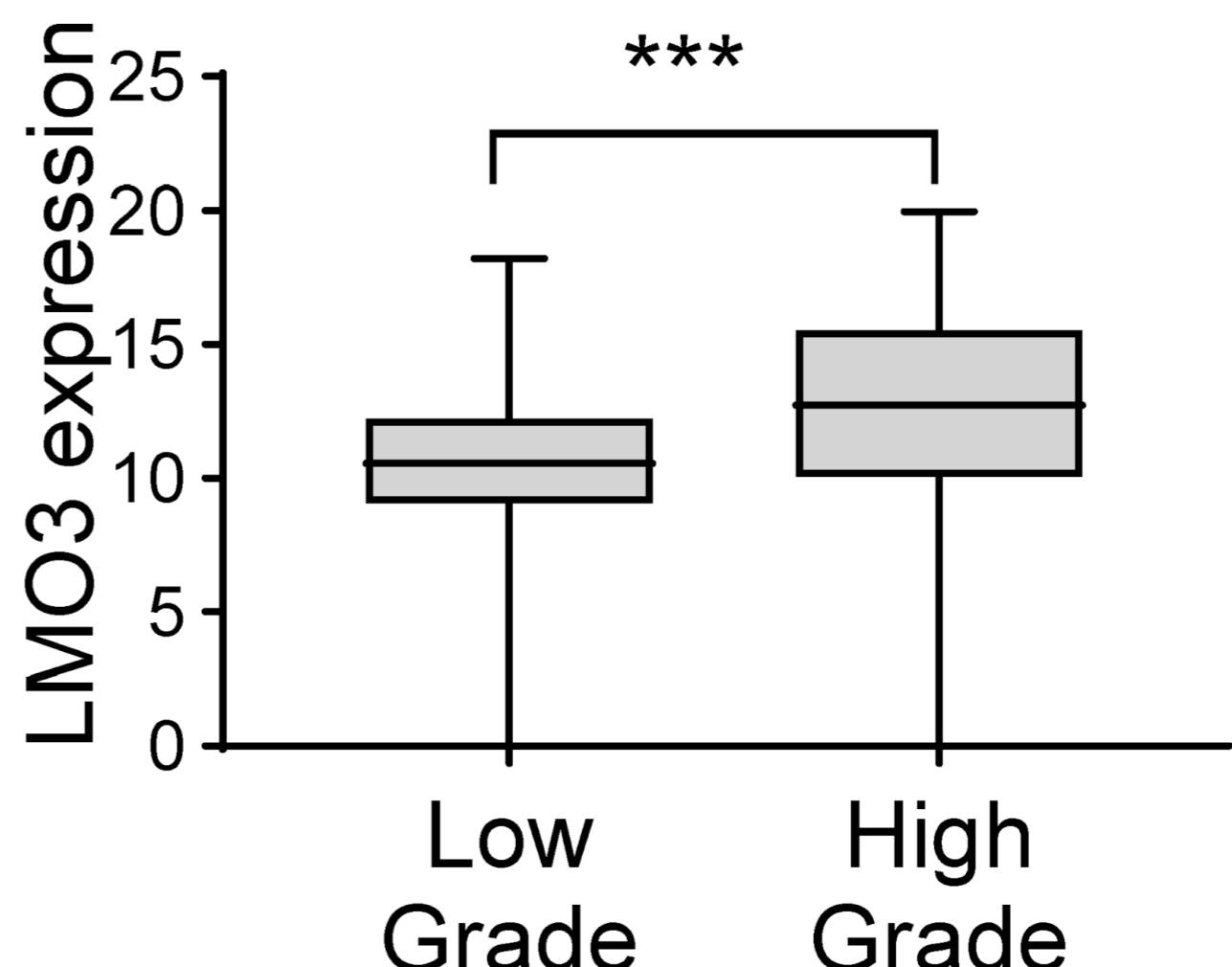

**(I)**

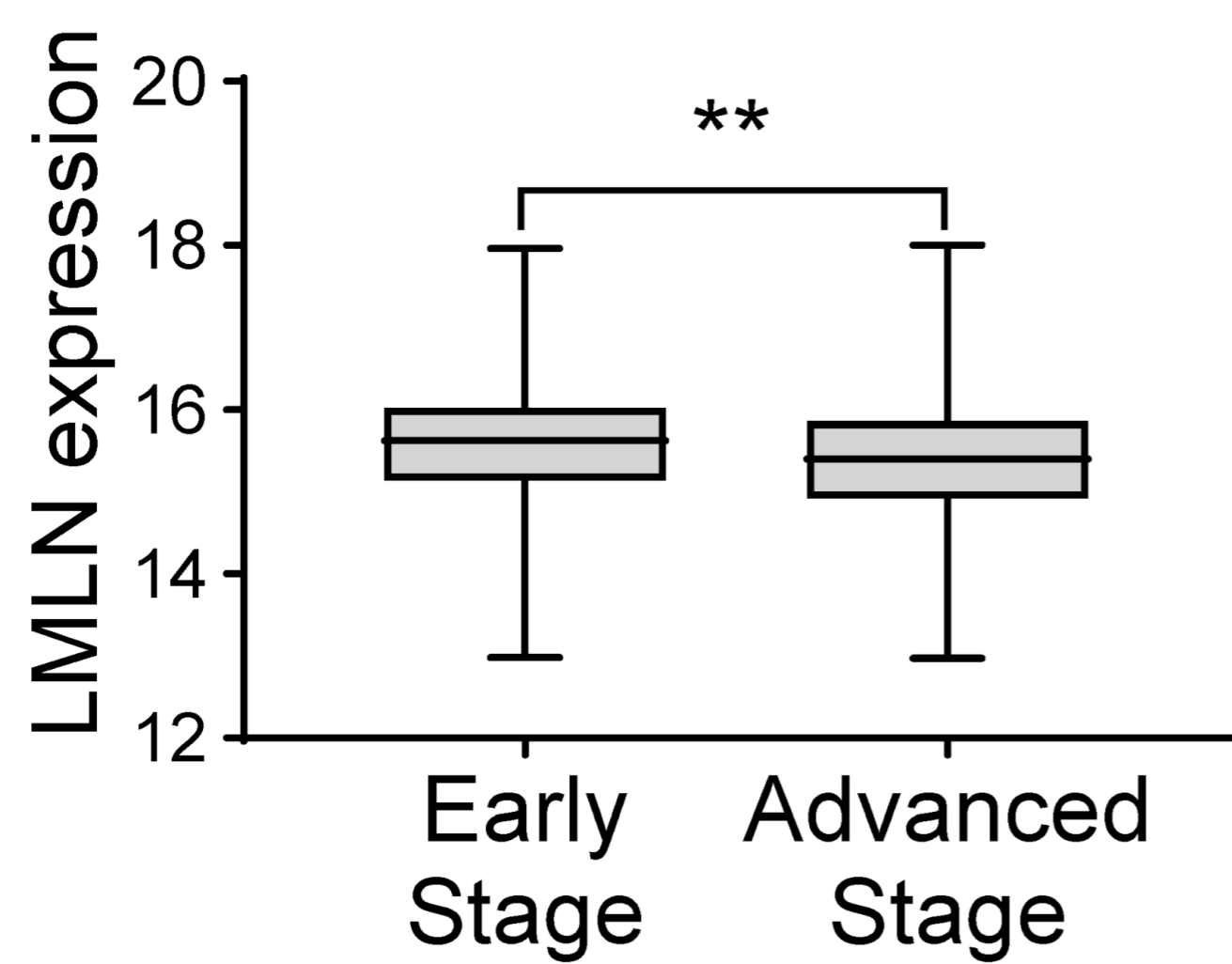

**(J)**

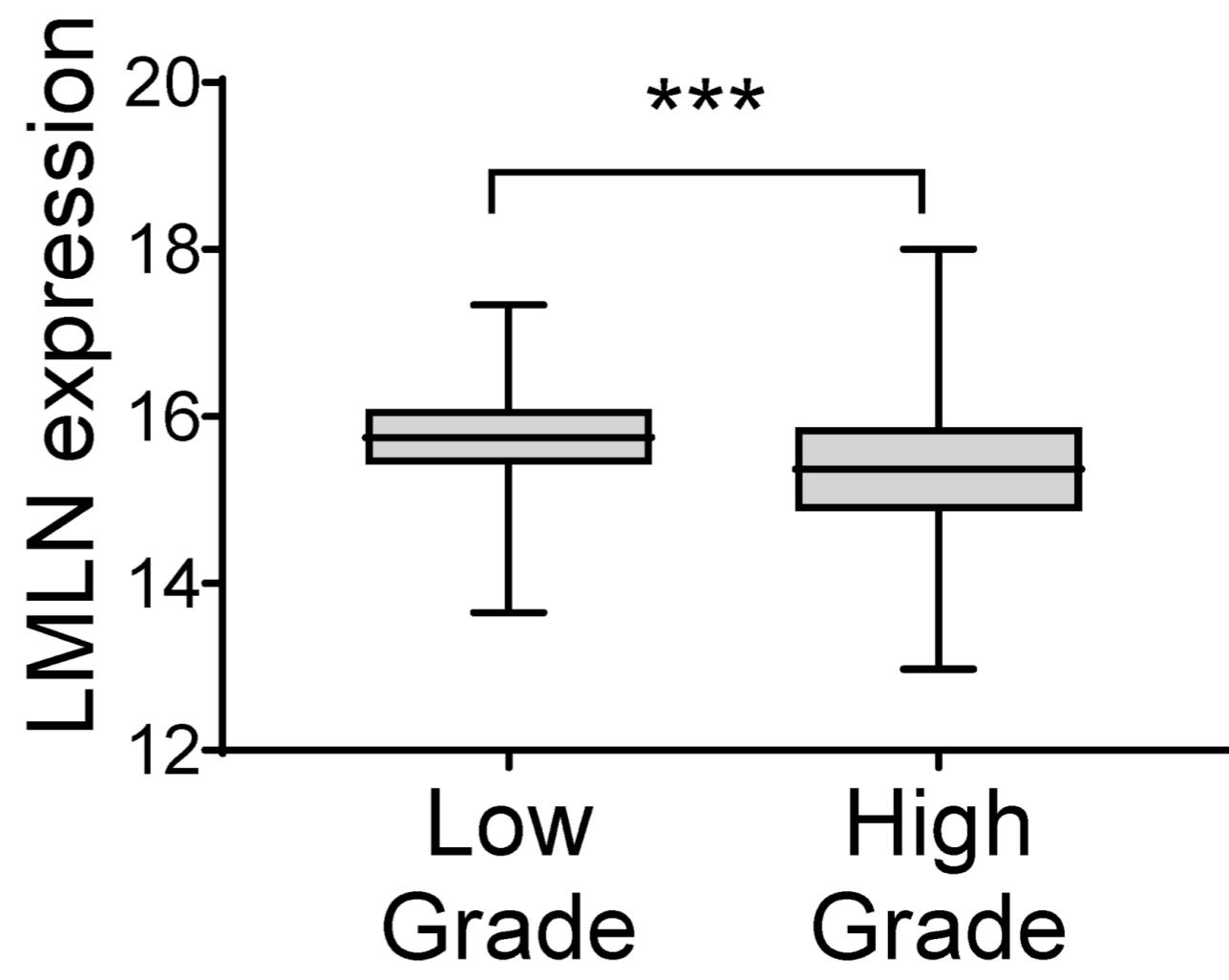

Supplement: Supplementary file 2 — Figure S2: Risk score and prognostic gene expression across tumor stages and grades. Box plots show the distribution of the risk score and expression levels of associated prognostic genes across tumor stage and grade categories. Statistical significance was assessed using Student's t‐test; *p < 0.05, **p < 0.01, ***p < 0.001. (A, C, E, G, I) Comparisons between early‐stage and advanced‐stage tumors. (B, D, F, H, J) Comparisons between low‐grade and high‐grade tumors. [file CNR2-8-e70313-s002.pdf]

**(A)**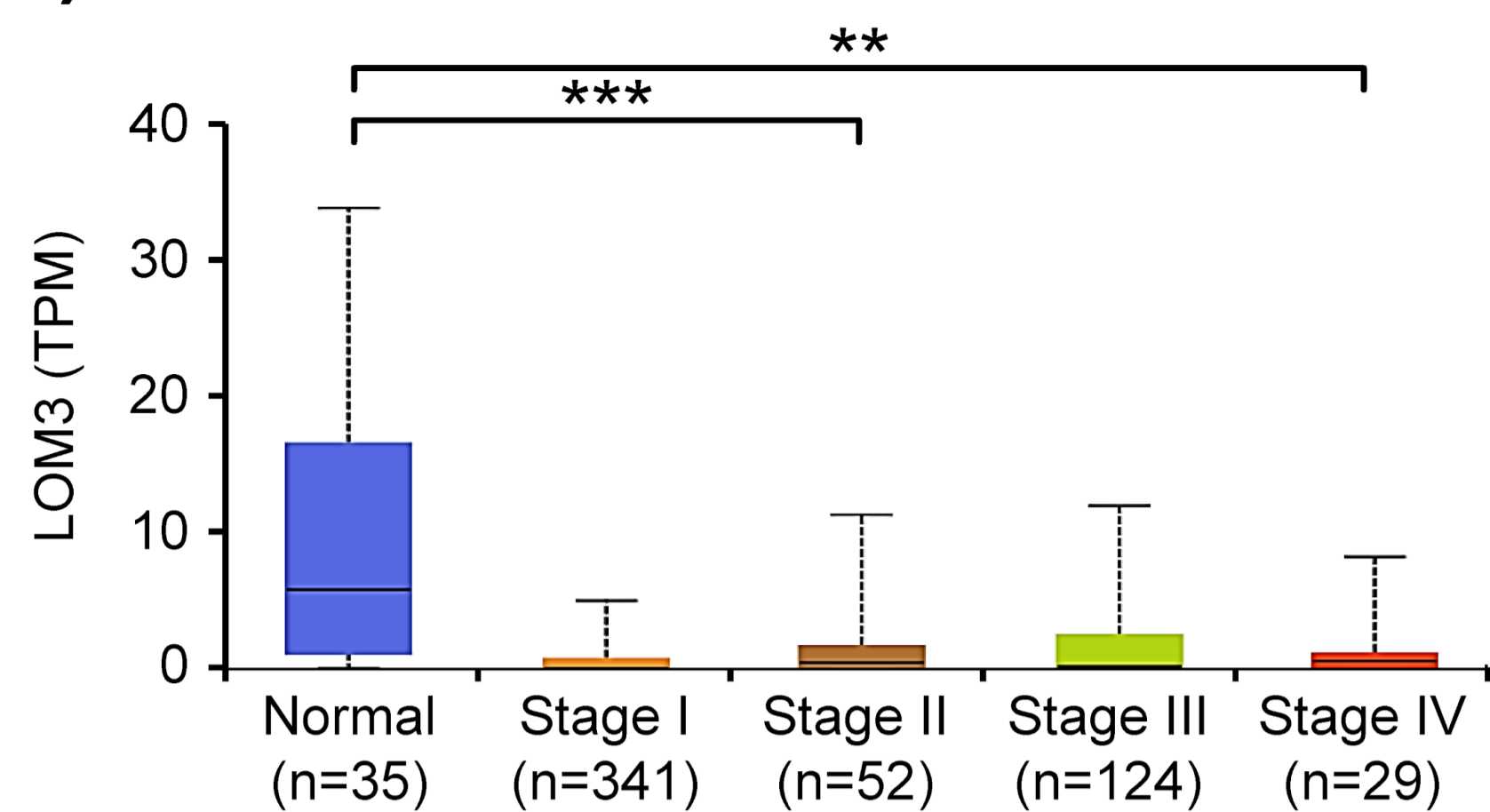**(B)**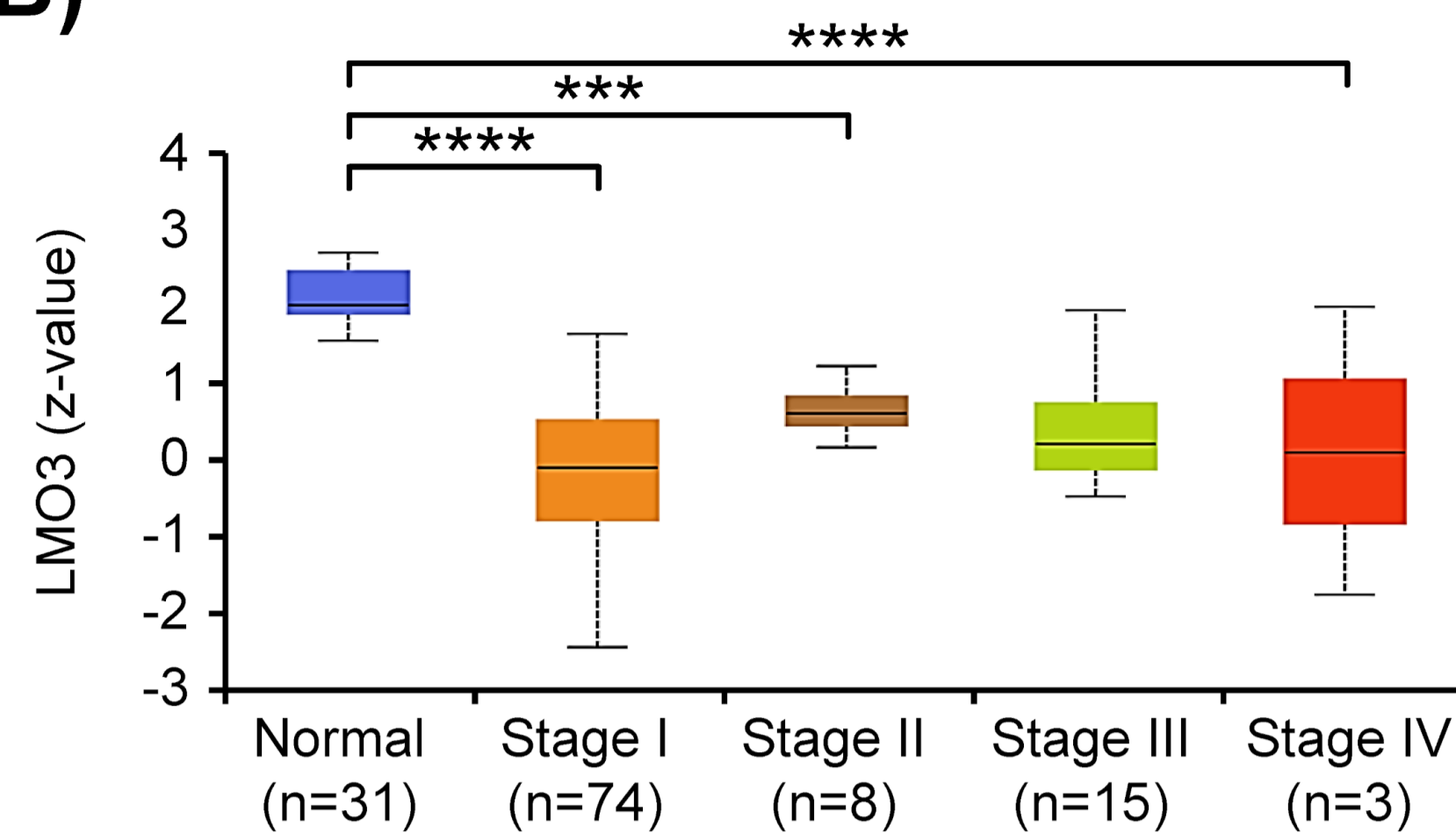**(C)**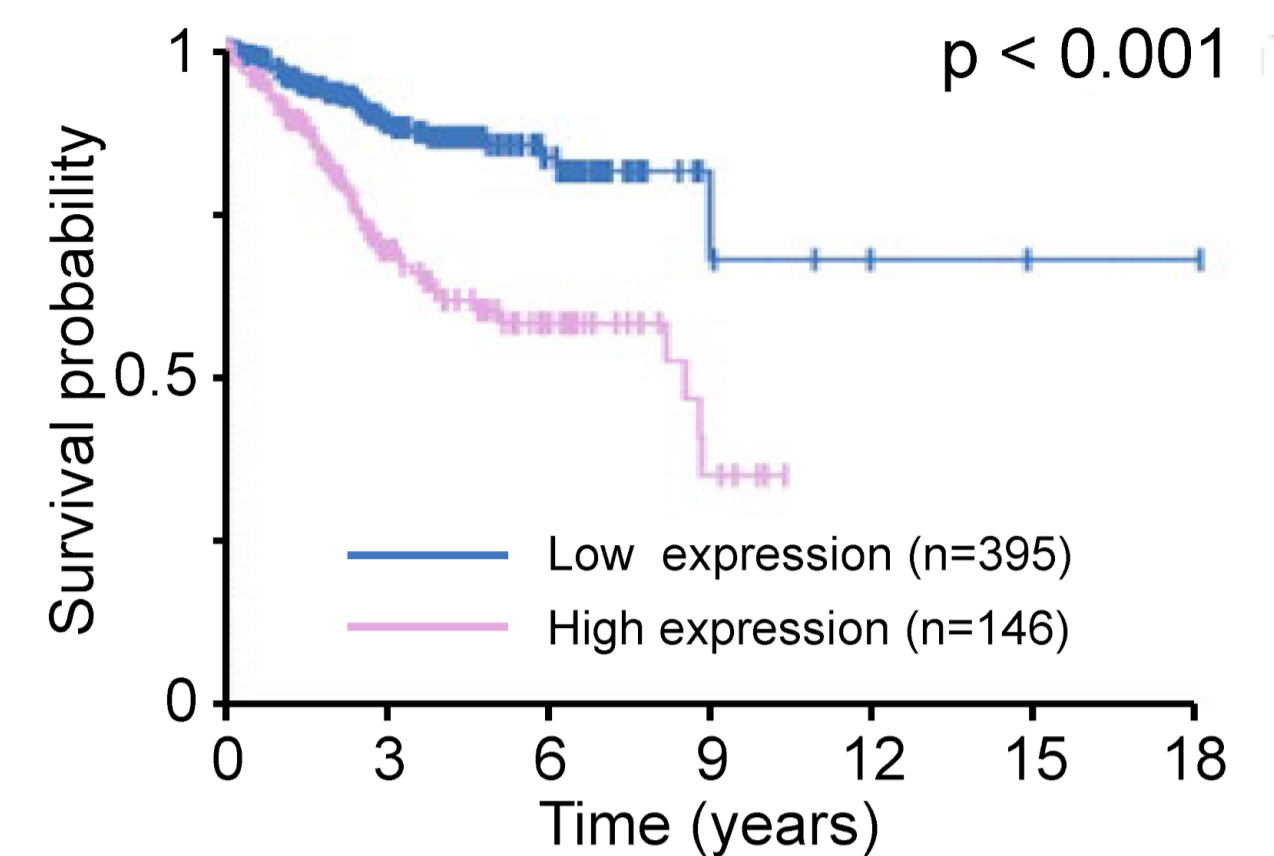**(D)**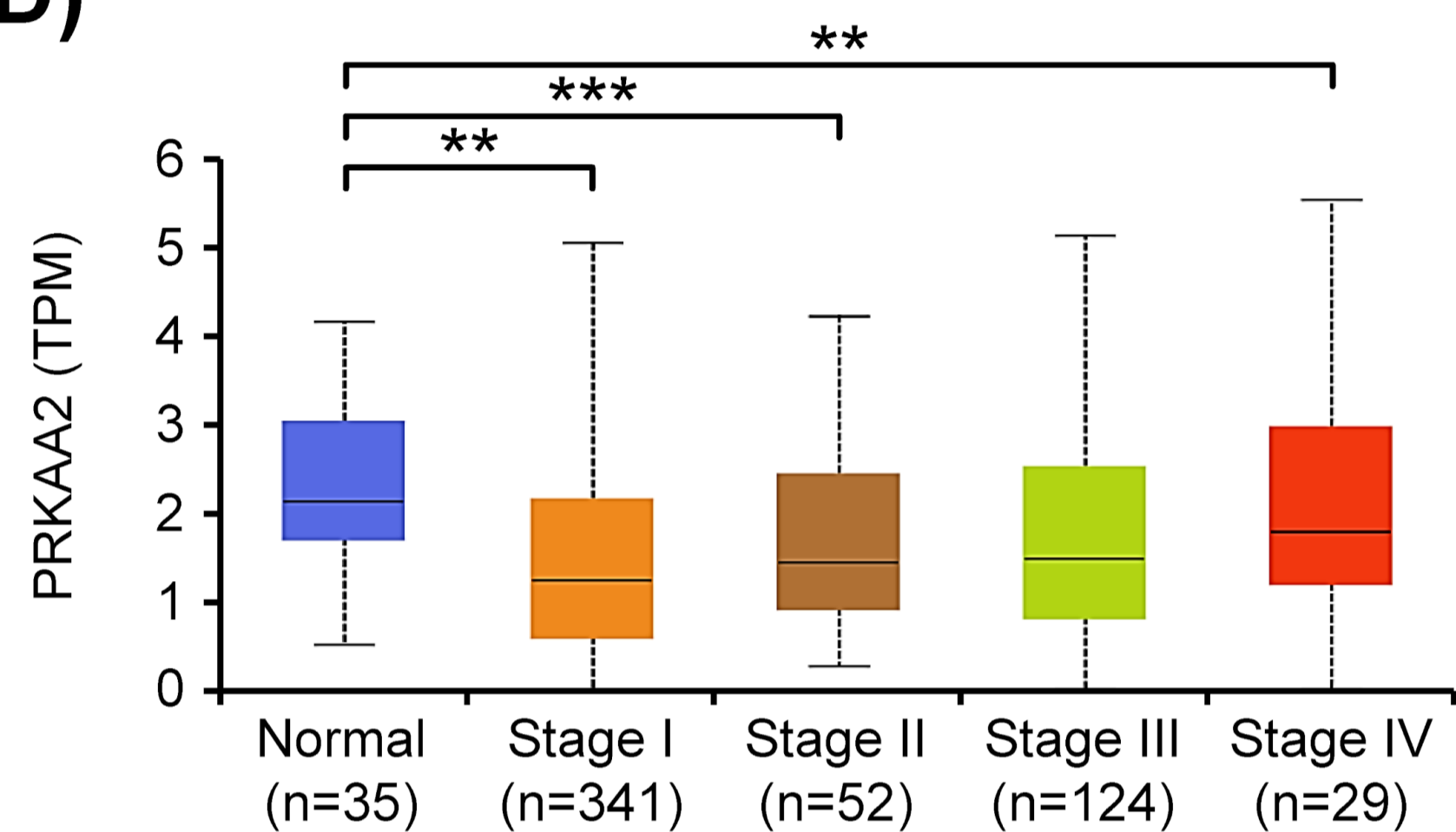**(E)**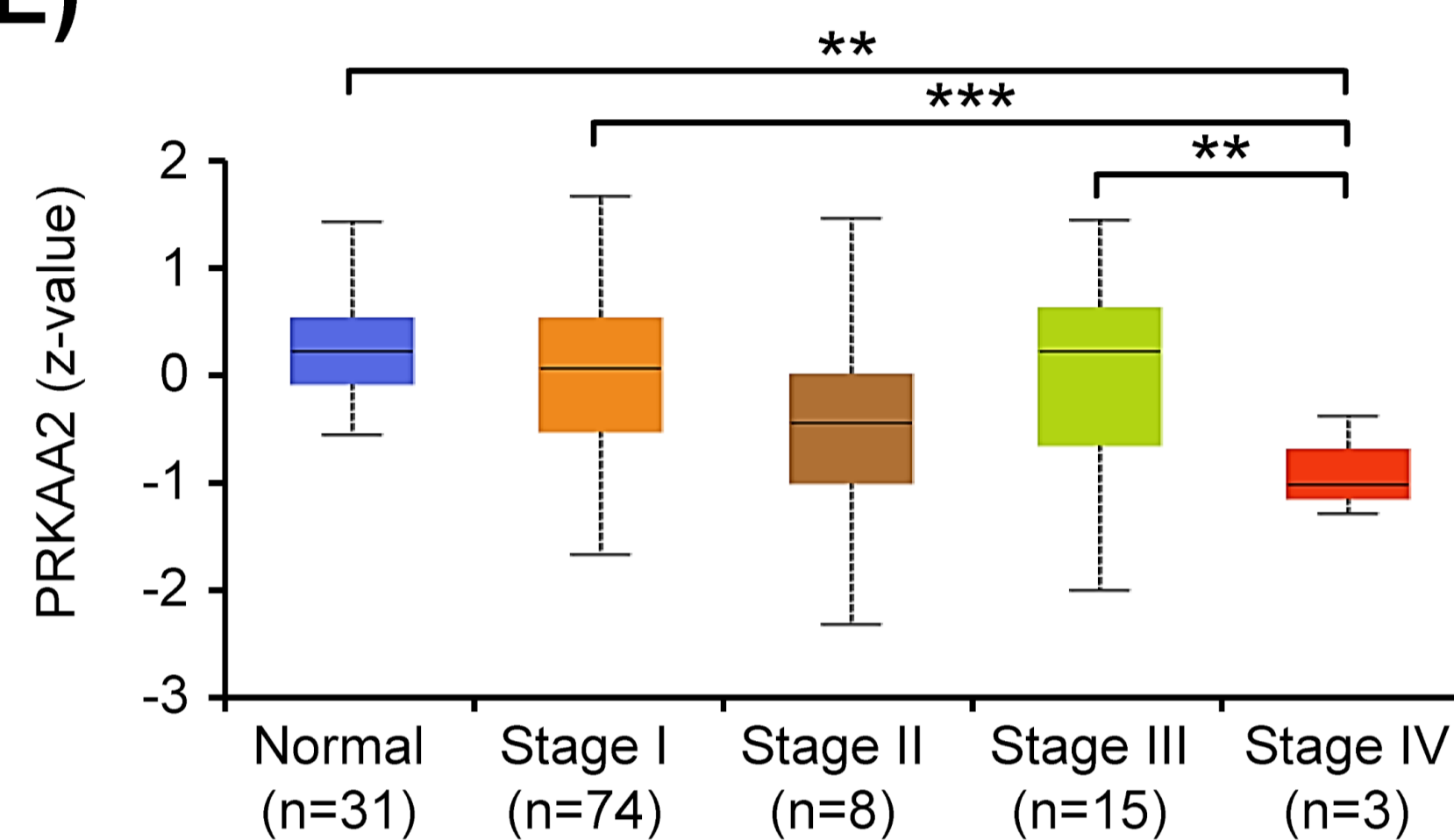**(F)**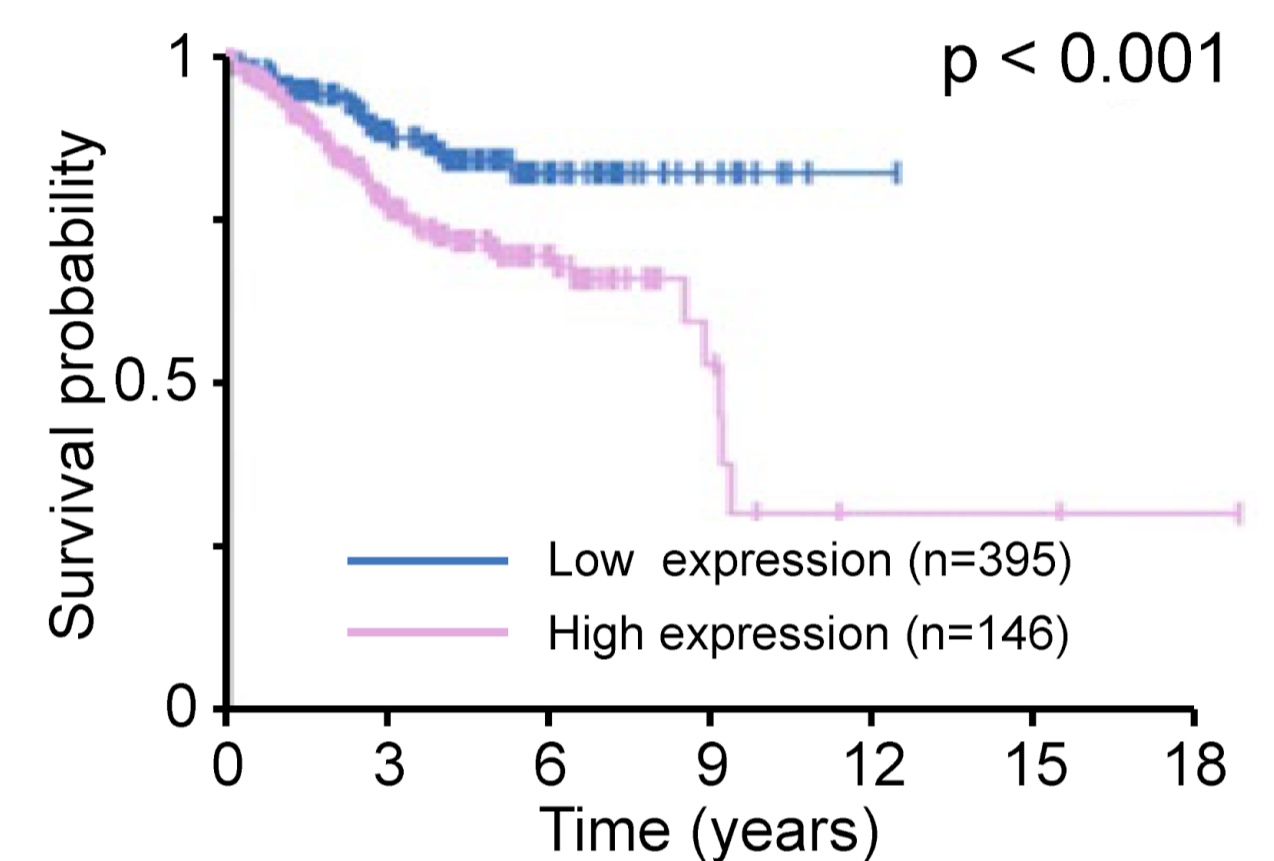**(G)**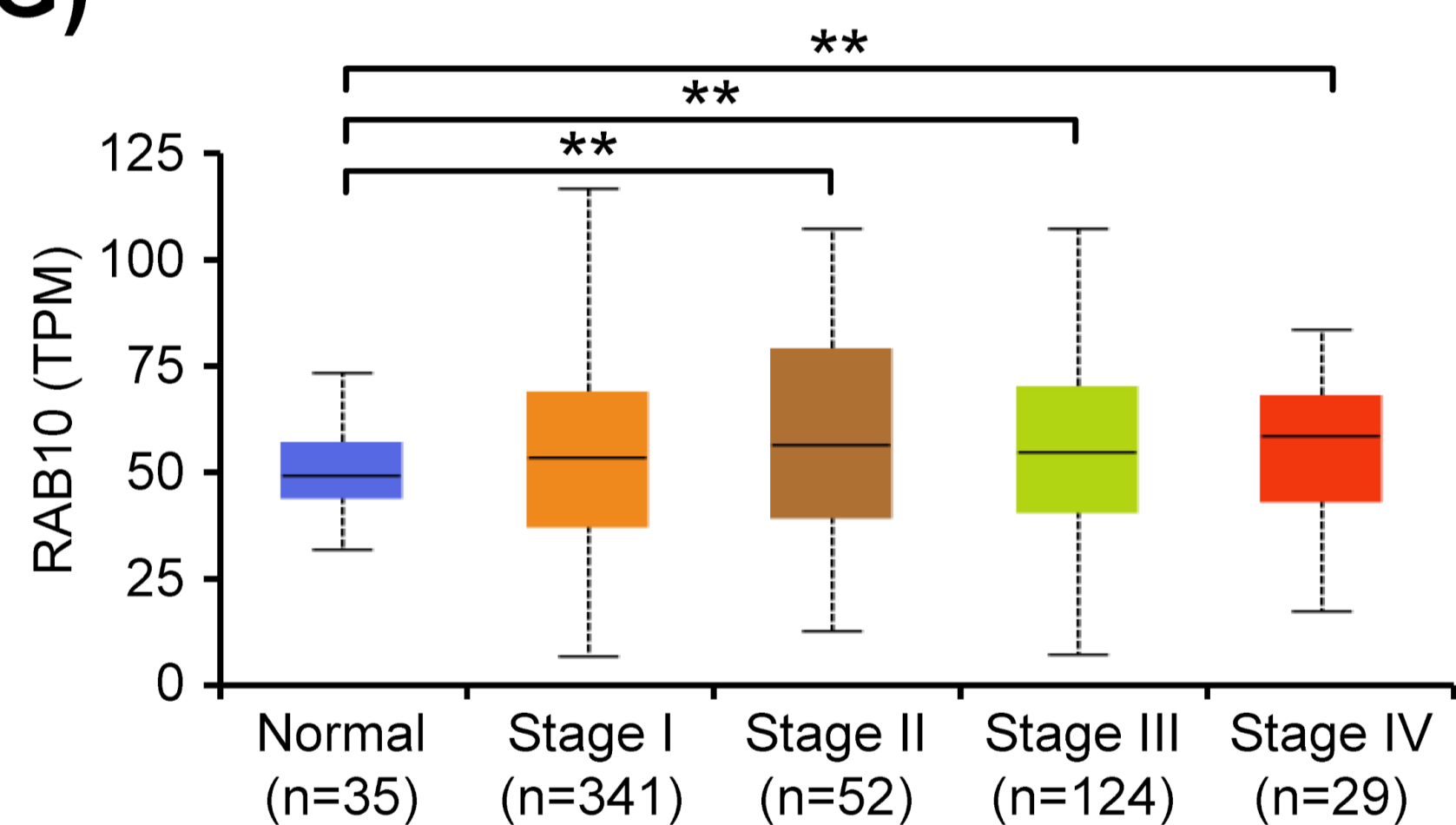**(H)**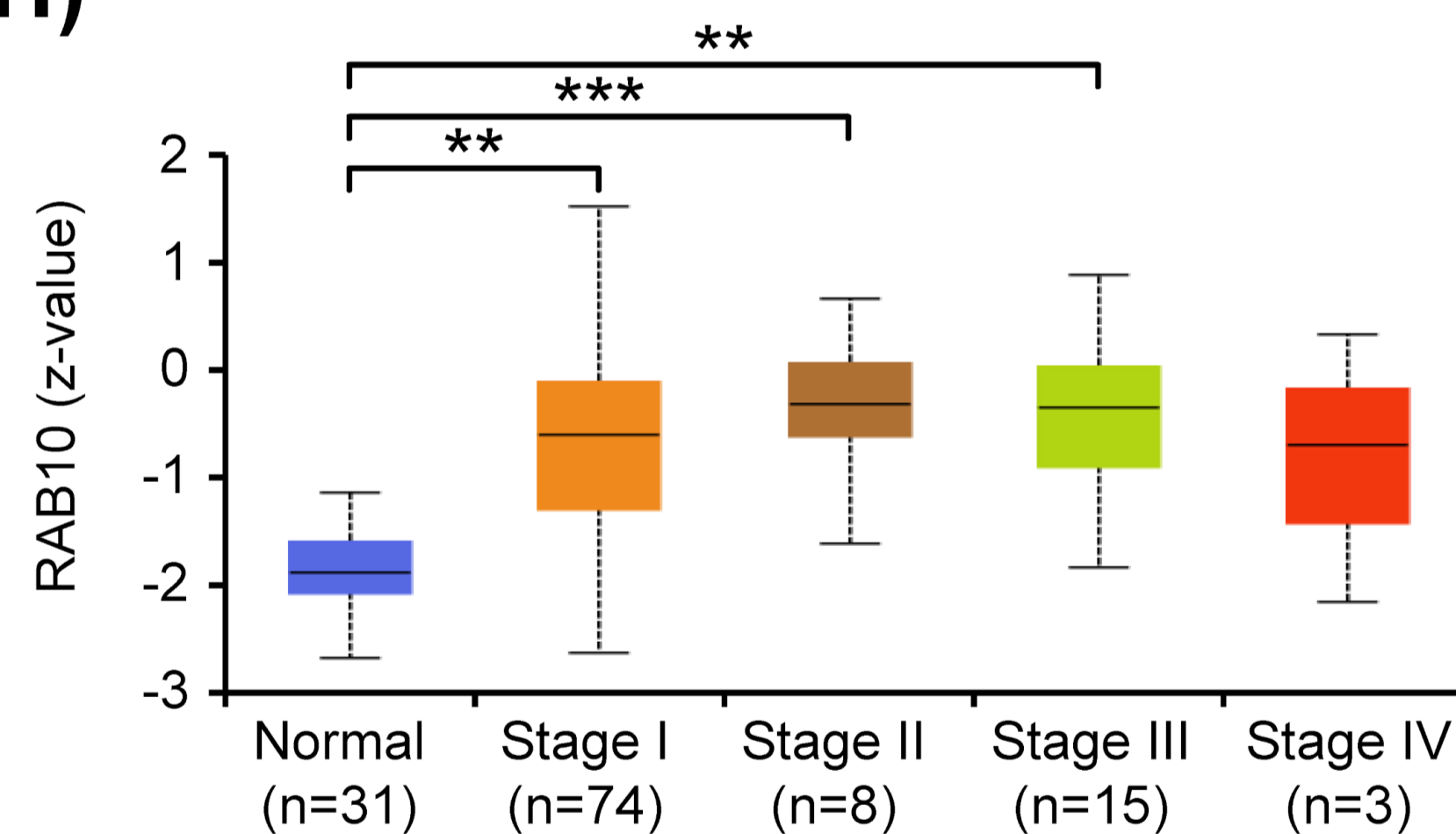**(I)**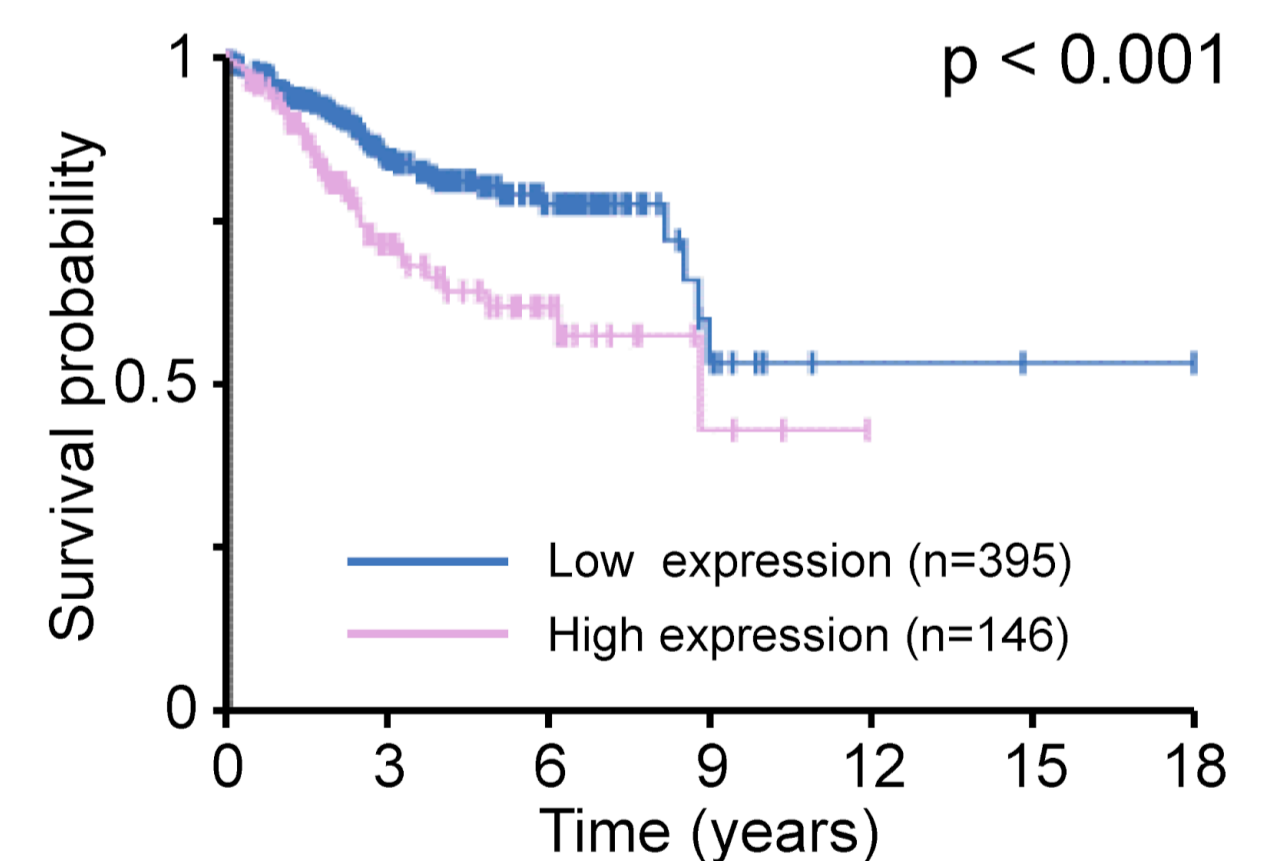**(J)**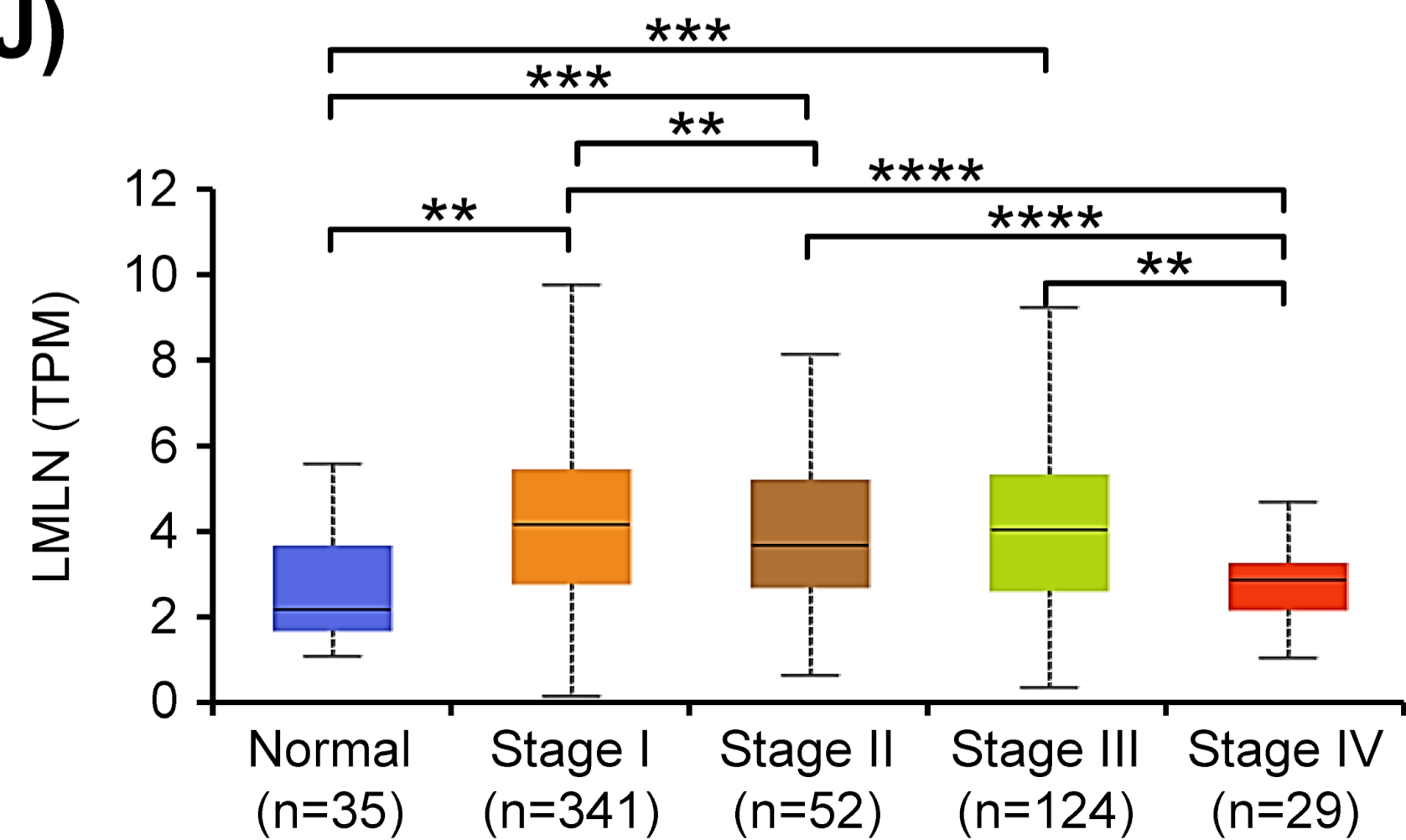**(K)**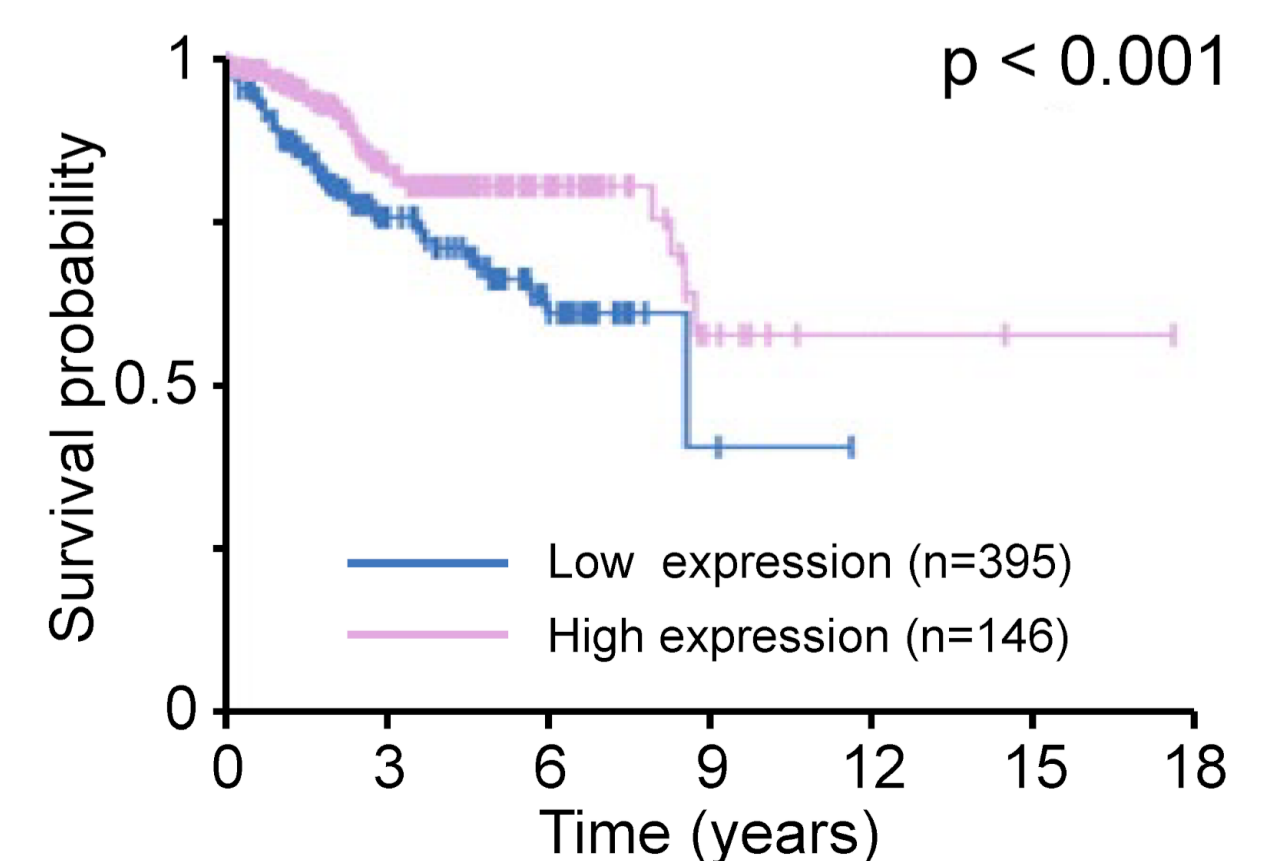

Supplement: Supplementary file 3 — Figure S3: Stagewise expression and survival profiles of risk model genes. (A, D, G, J) mRNA and (B, E, H) protein levels from UALCAN platform. (C, F, I, K) Survival curves from HPA (human protein atlas). Assessed genes were LMO3 (A, B, C), PRKAA2 (D, E, F), Rab10 (G, H, I), and LMLN (J, K). Significance levels indicated as follows: *p < 0.05, **p < 0.01, ***p < 0.001 and ****p < 0.0001. [file CNR2-8-e70313-s006.pdf]

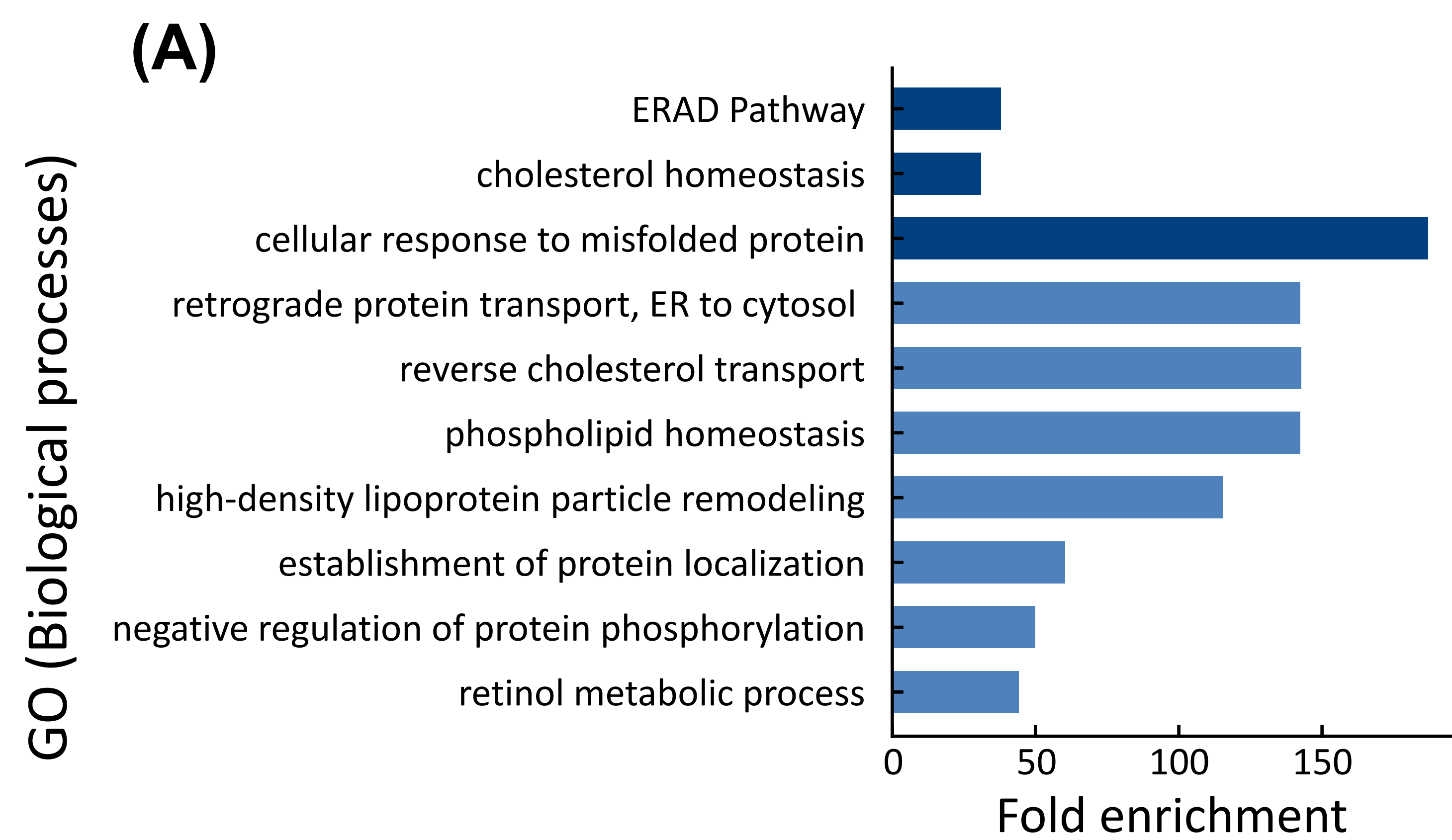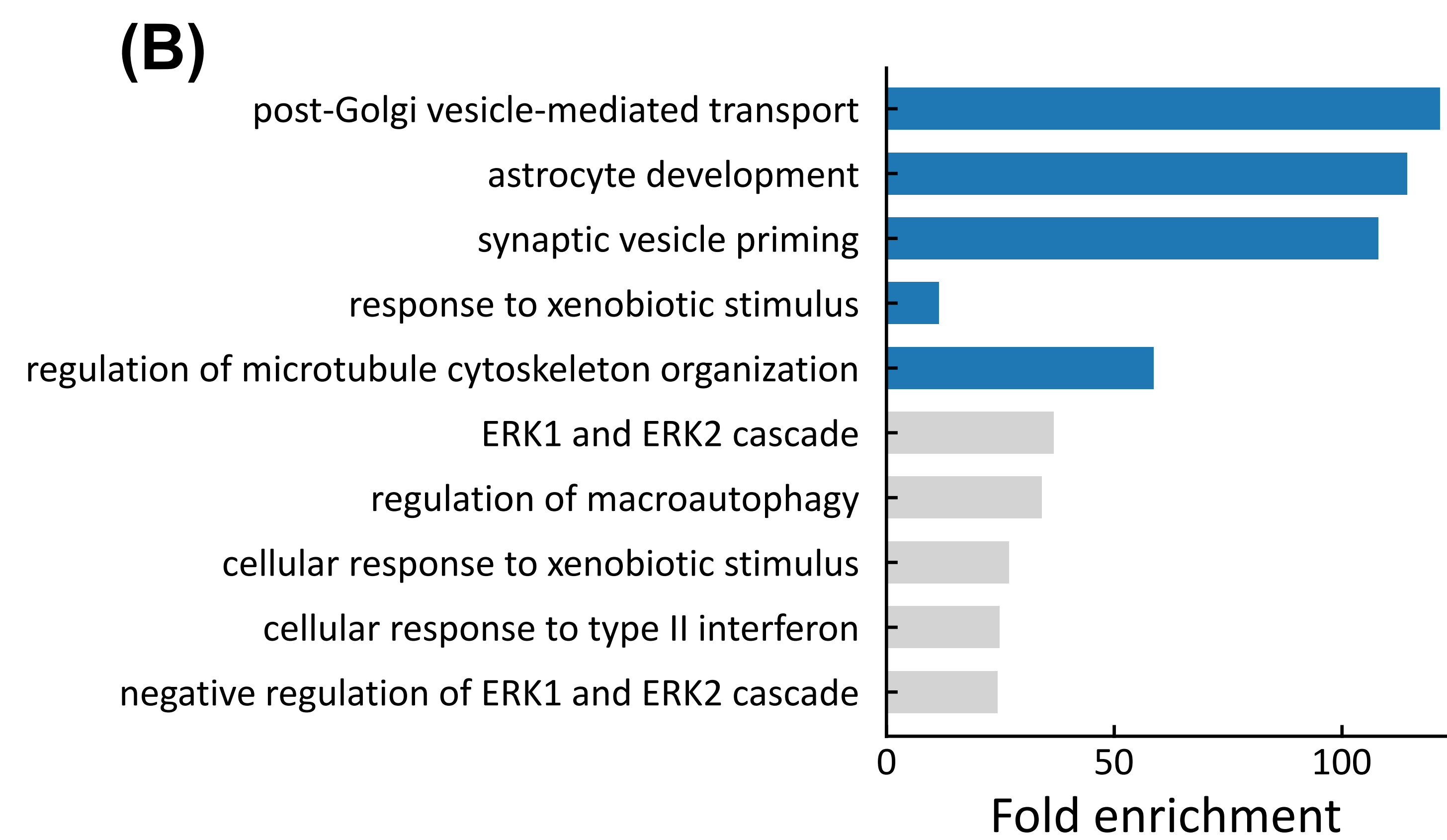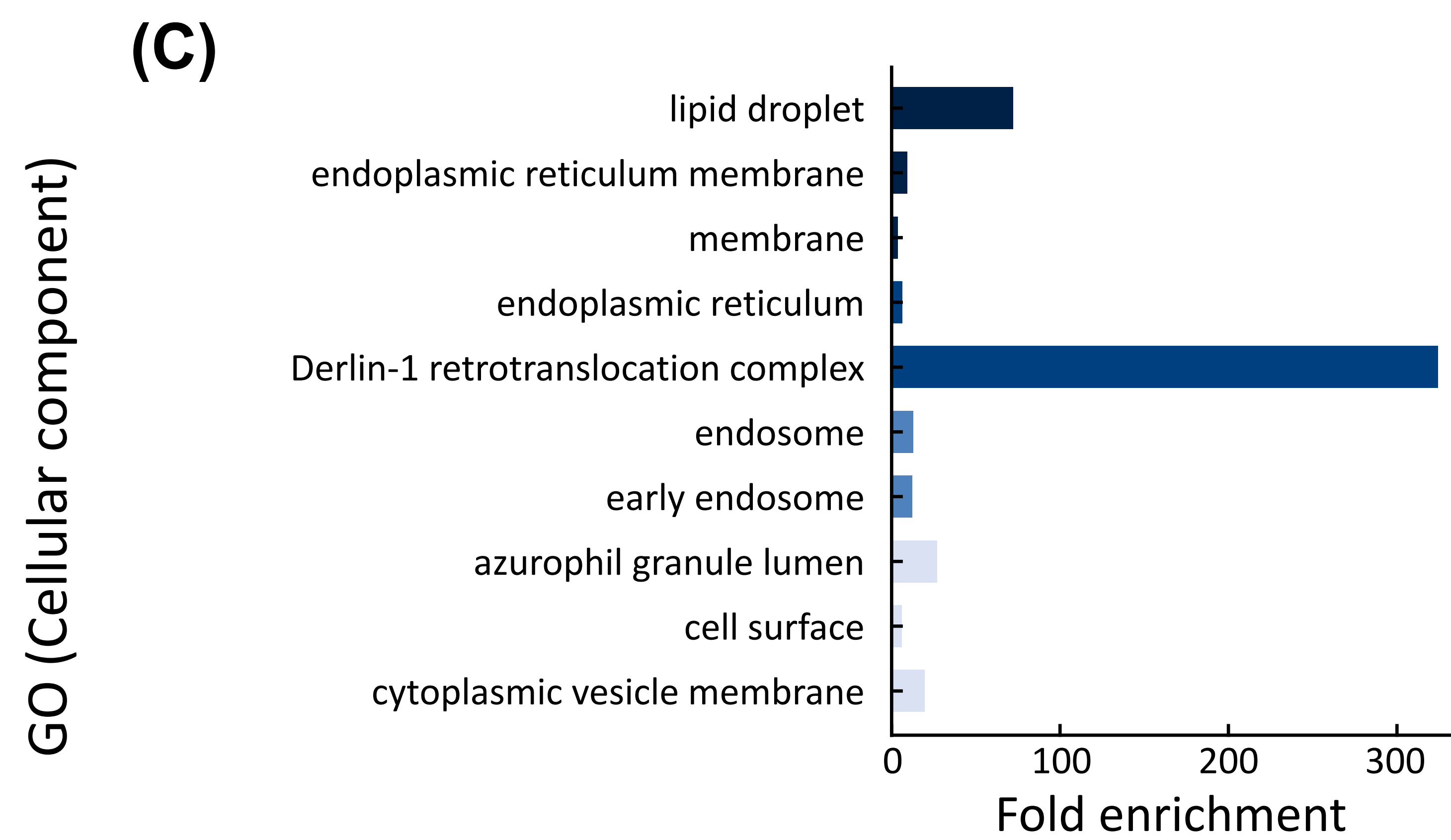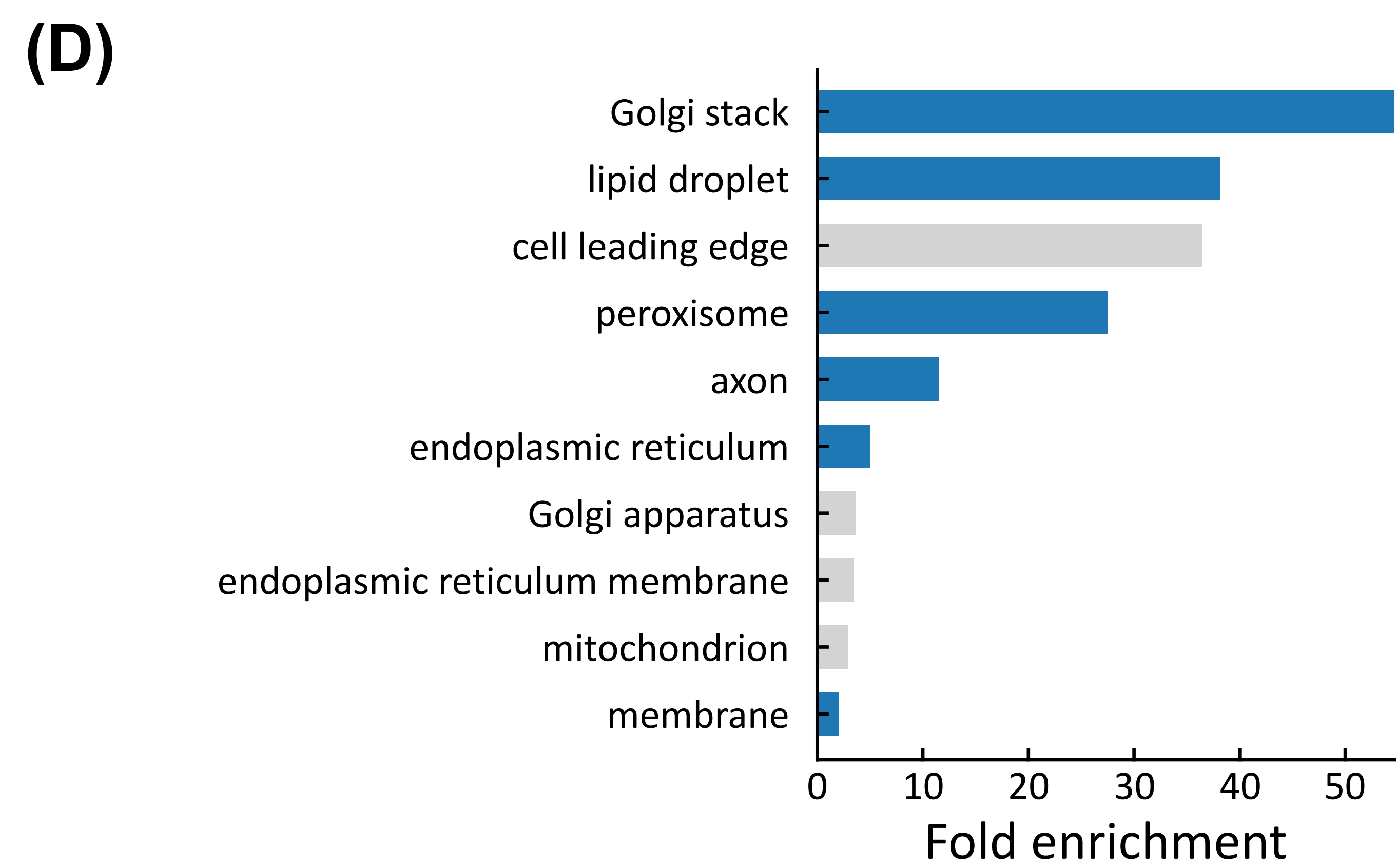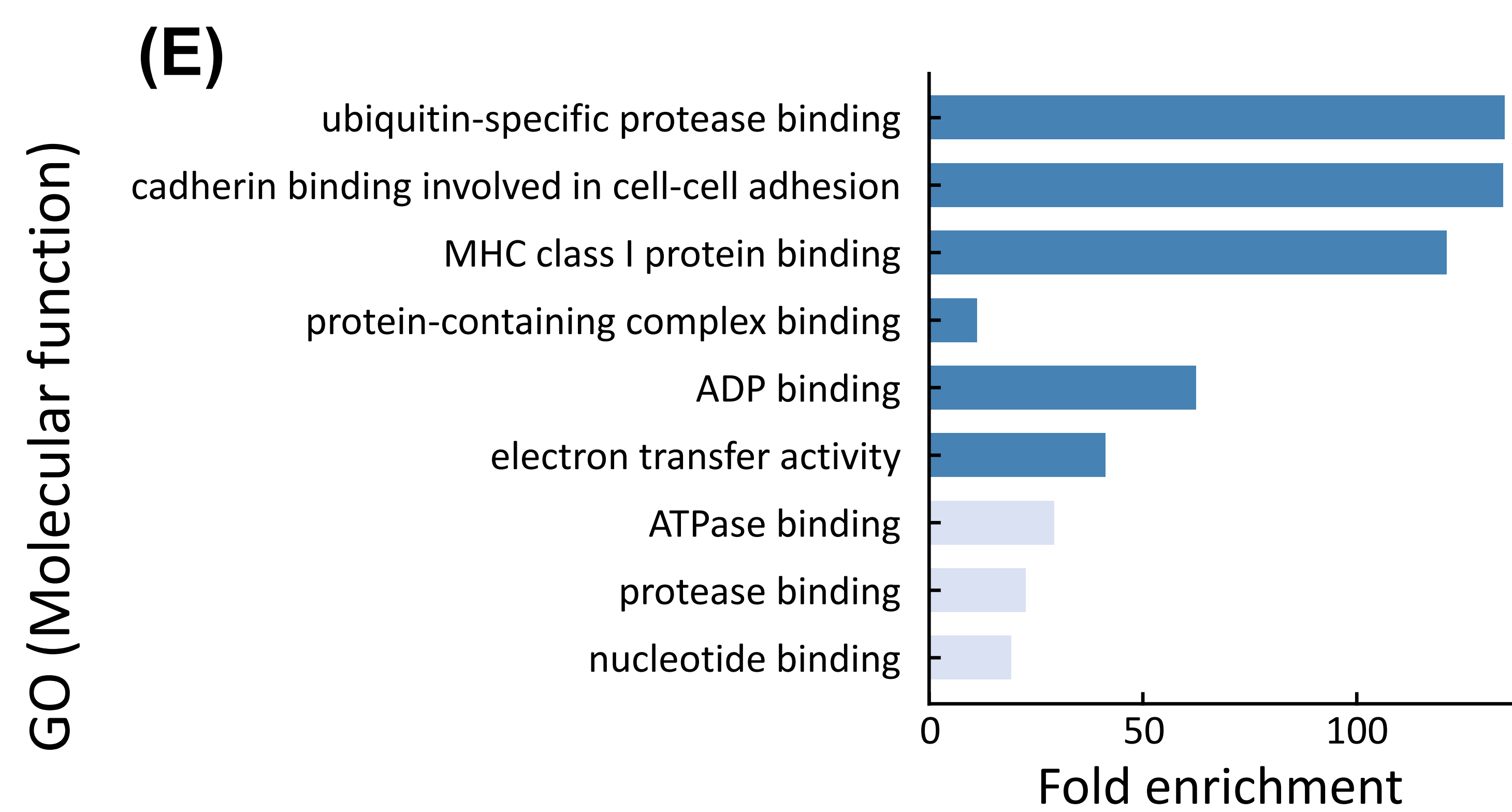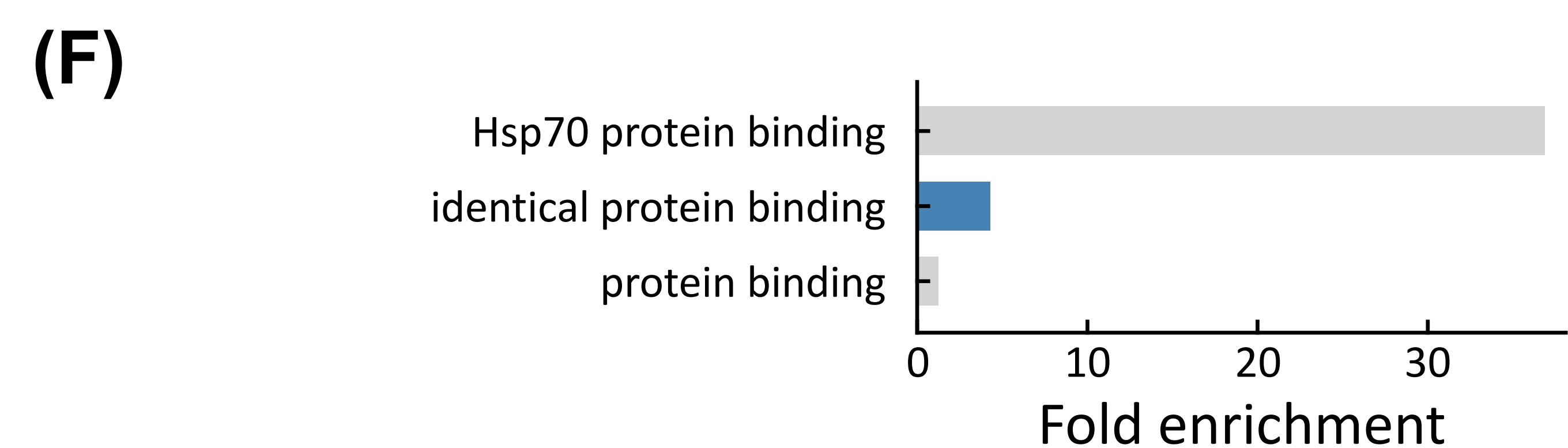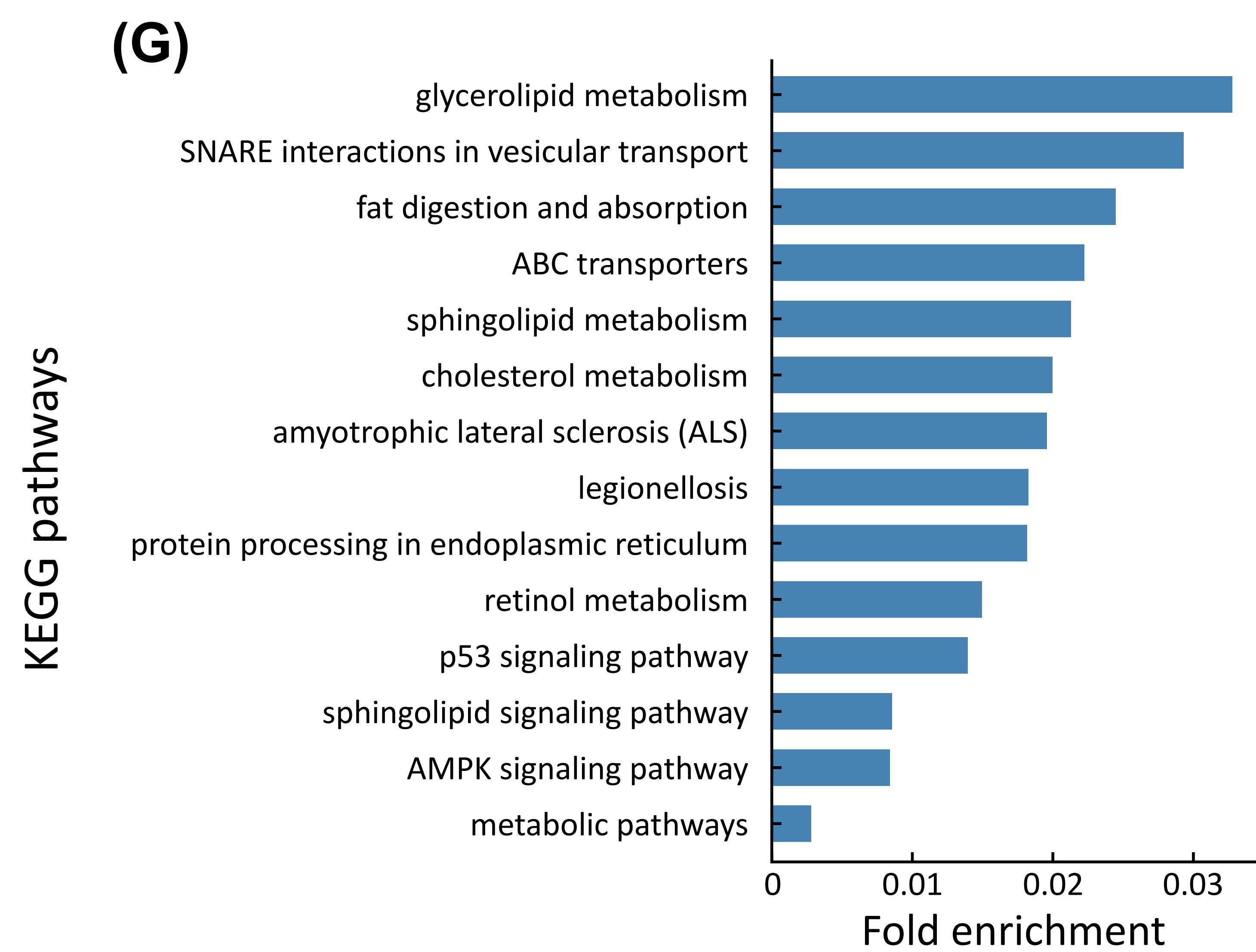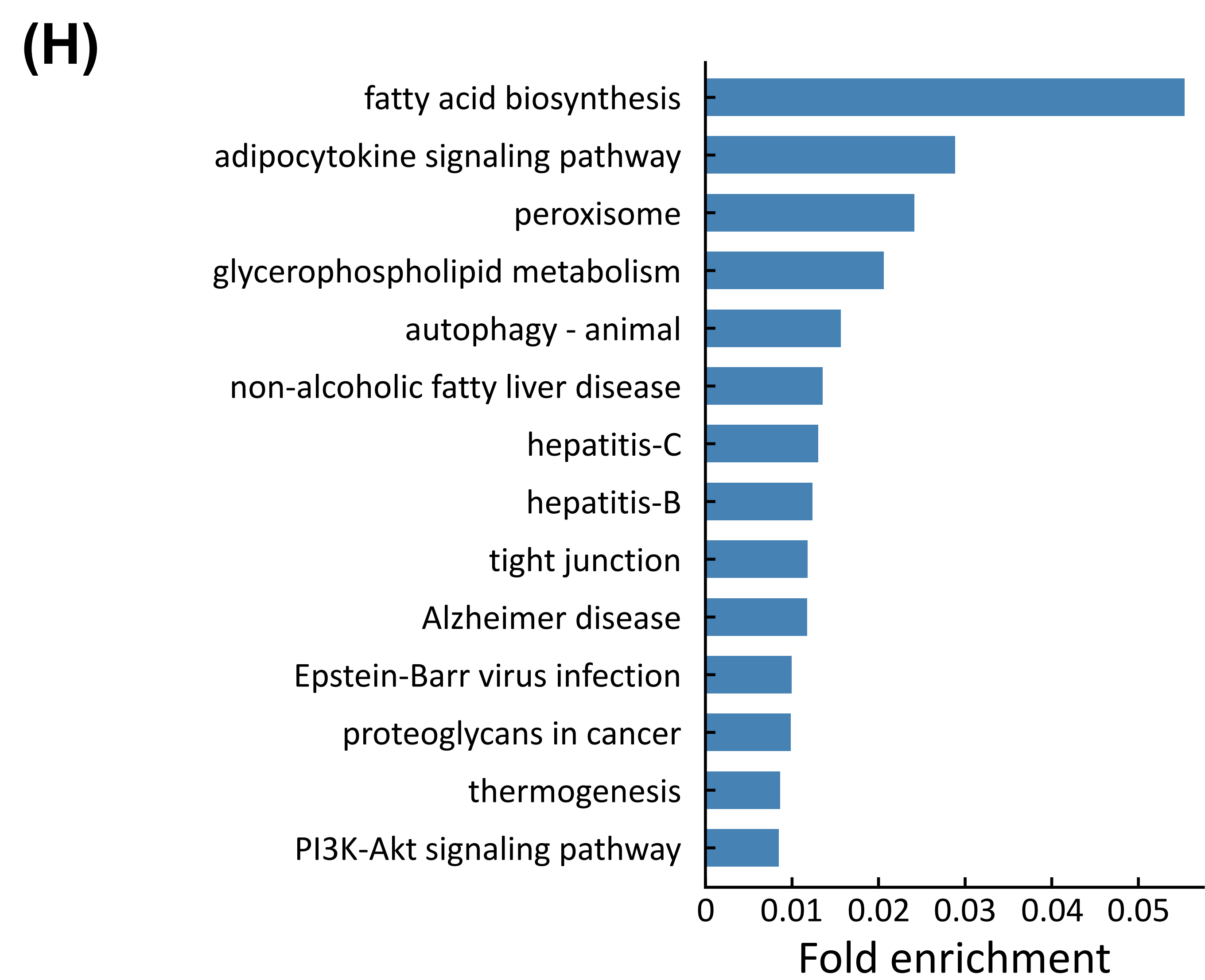

Supplement: Supplementary file 4 — Figure S4: Functional enrichment analysis of survival‐associated DE‐LDAGs in EC. GO term and KEGG pathway enrichment analyses were performed separately for upregulated and downregulated survival‐associated DE‐LDAGs using DAVID and KOBAS. Panels show enriched biological processes (A: upregulated, B: downregulated), cellular components (C: upregulated, D: downregulated), molecular functions (E: upregulated, F: downregulated), and KEGG pathways (G: upregulated, H: downregulated). Bar colors indicate statistical significance: dark blue bars represent FDR < 0.05, lighter blue bars represent p < 0.05 but FDR ≥ 0.05, and gray bars indicate p > 0.05. [file CNR2-8-e70313-s001.pdf]

(A)

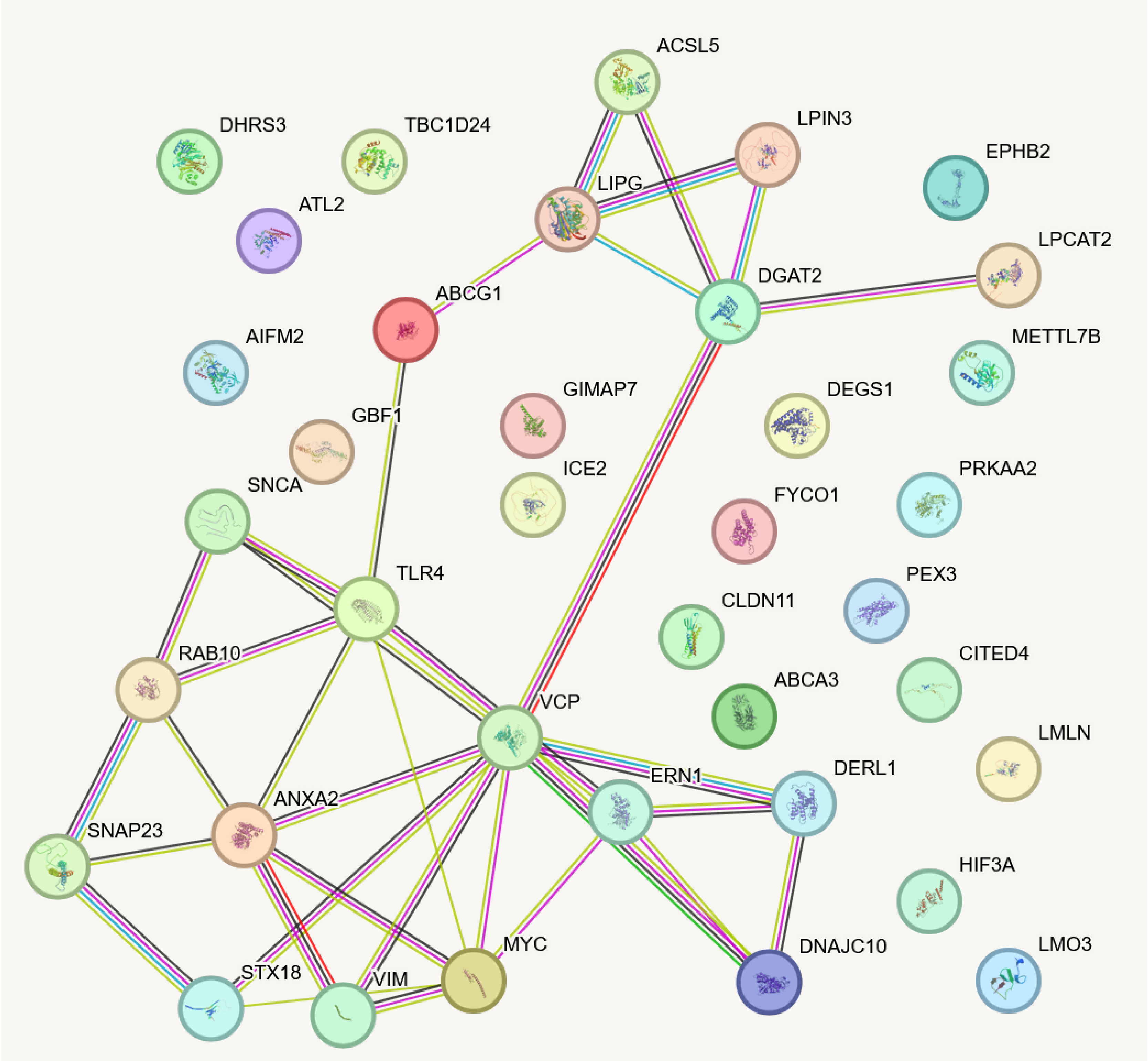

(B)

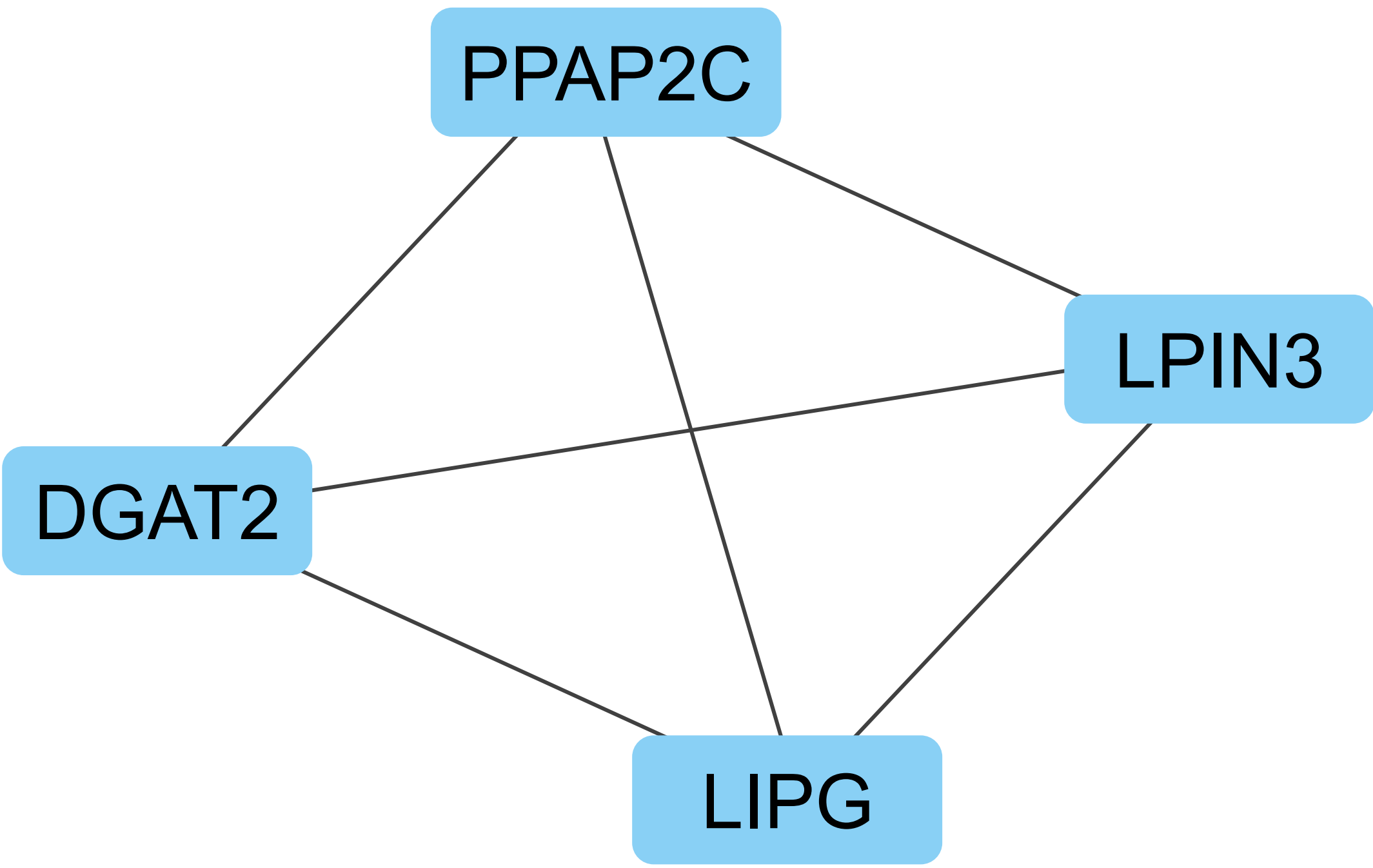

(C)

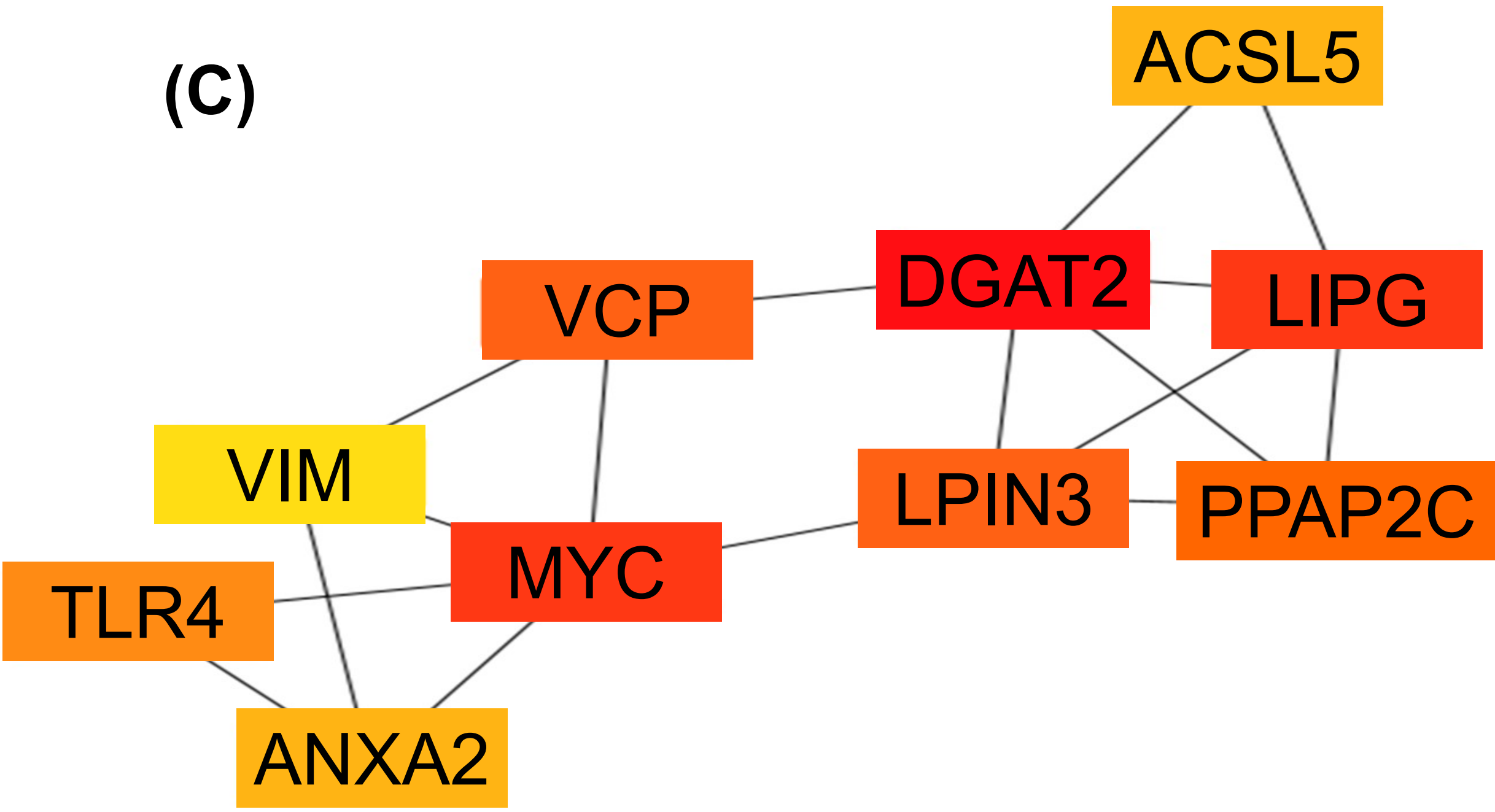

Supplement: Supplementary file 5 — Figure S5: PPI network and module analysis of survival‐associated DE‐LDAGs in EC. (A) PPI network constructed using STRING. (B) Key module identified using the MCODE plug‐in in Cytoscape. (C) Top 10 hub genes identified using the cytoHubba plug‐in. [file CNR2-8-e70313-s004.pdf]

(A)

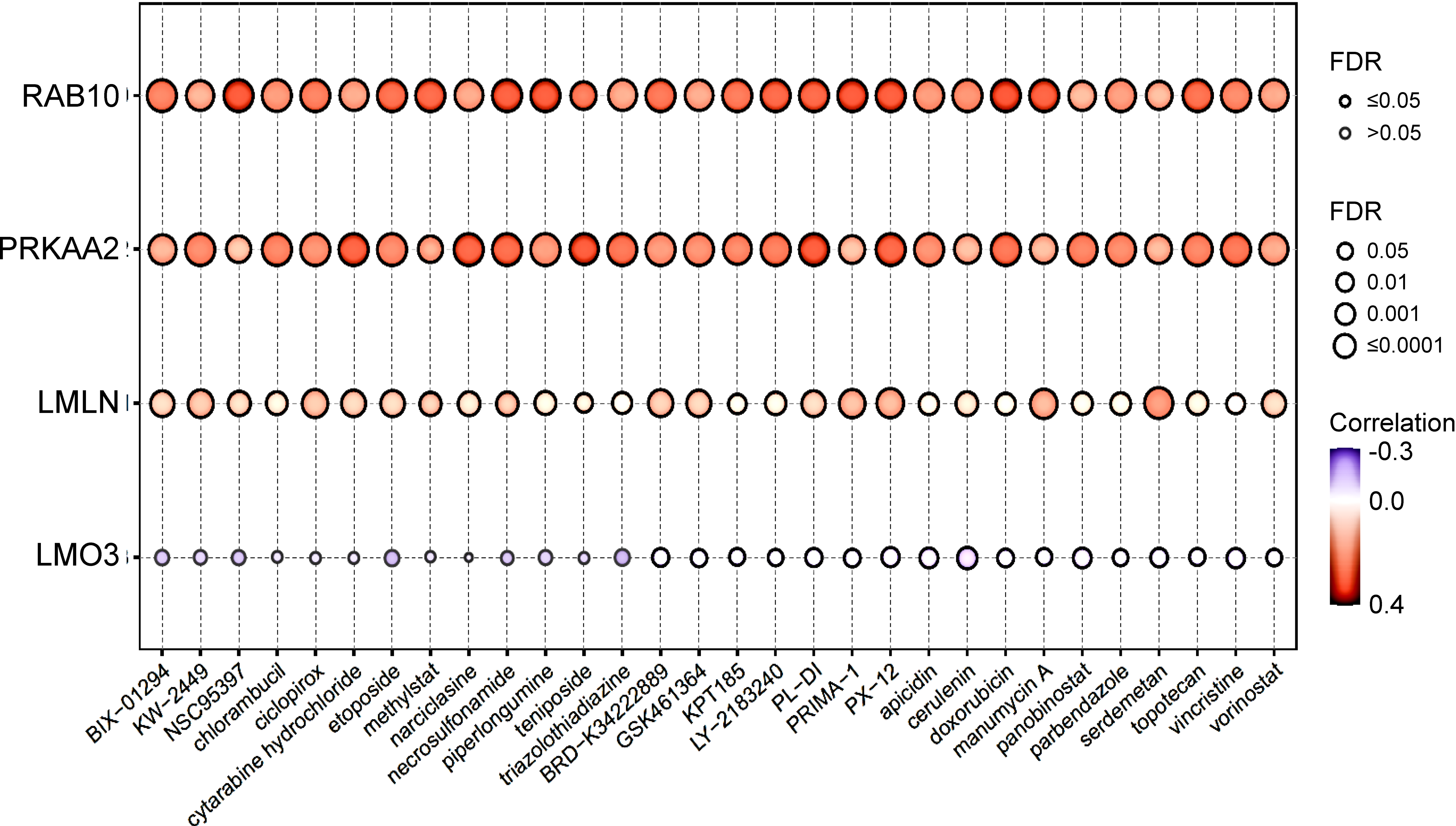

(B)

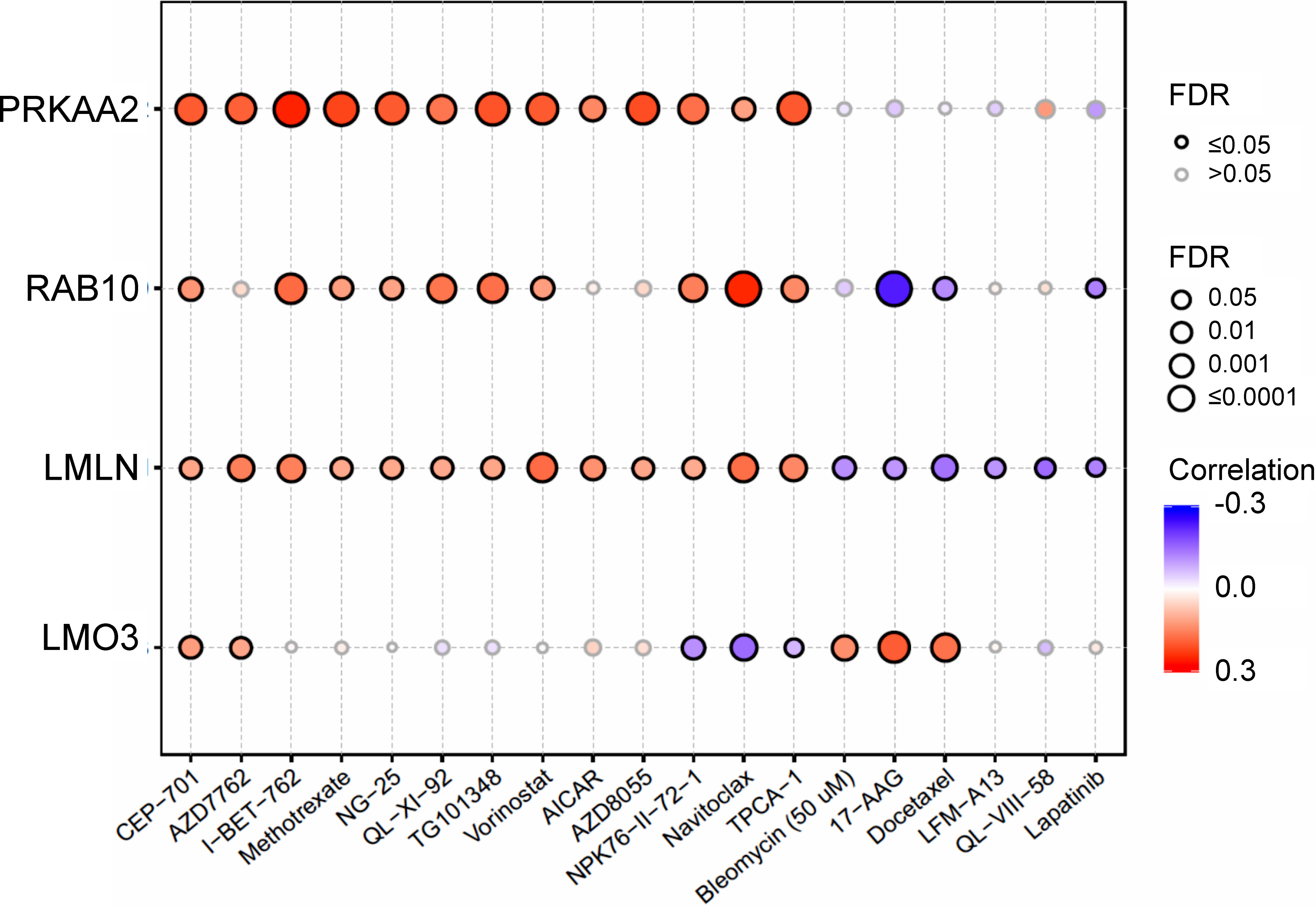

Supplement: Supplementary file 6 — Figure S6: Drug sensitivity analysis of 4‐gene prognostic signature. Correlation analysis between the 4‐gene LDAG signature (PRKAA2, RAB10, LMLN, LMO3) and drug response data from the GSCA platform. Panels show the top 30 drugs most significantly associated with the signature in the CTRP (A) and GDSC (B) datasets. Positive correlations indicate that higher signature expression is associated with reduced drug efficacy, whereas negative correlations suggest increased drug sensitivity. In the GSCA bubble plot, color intensity reflects correlation strength (red: positive; blue: negative), bubble size denotes FDR significance, and black outlines indicate FDR ≤ 0.05. [file CNR2-8-e70313-s005.pdf]

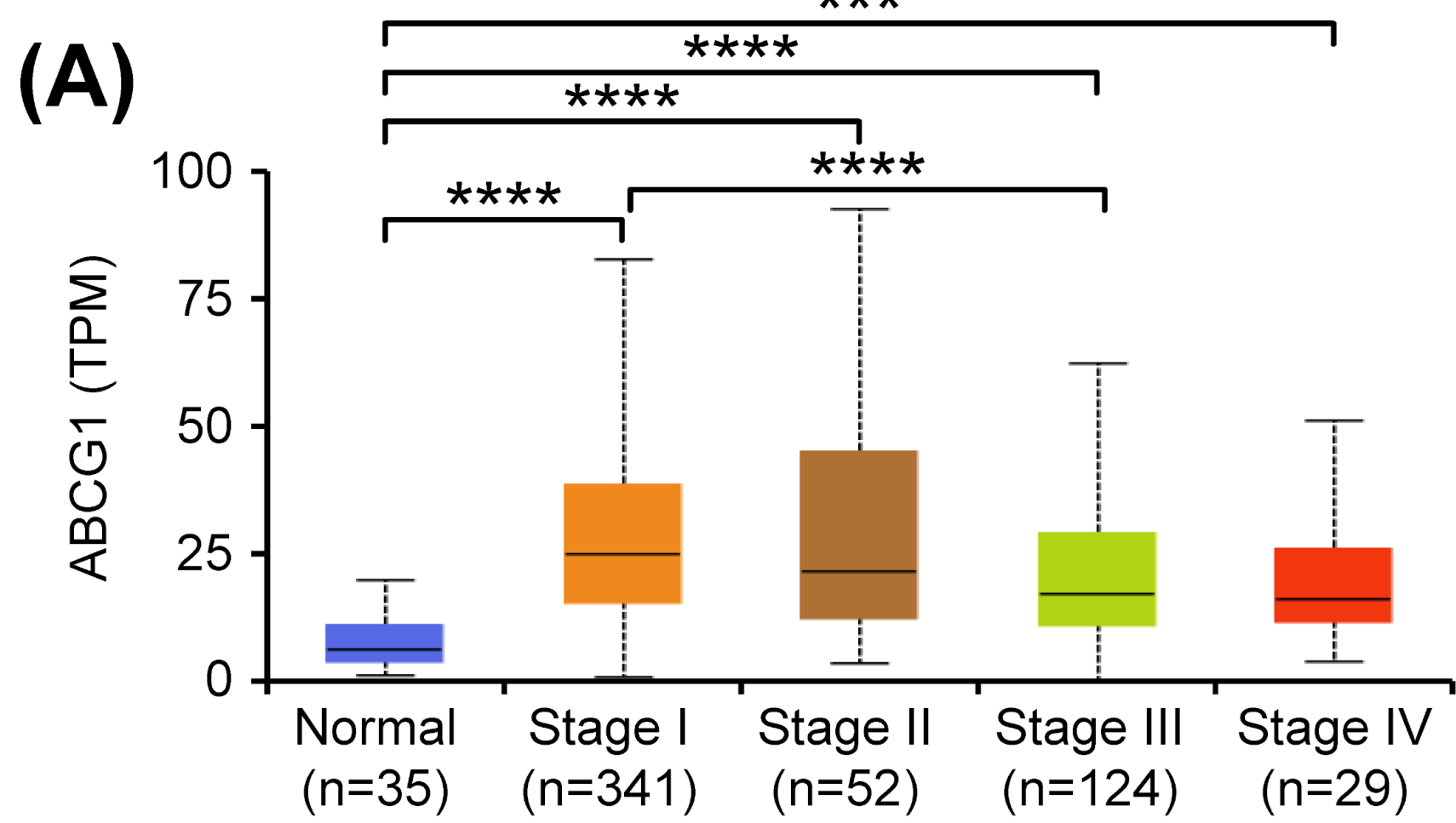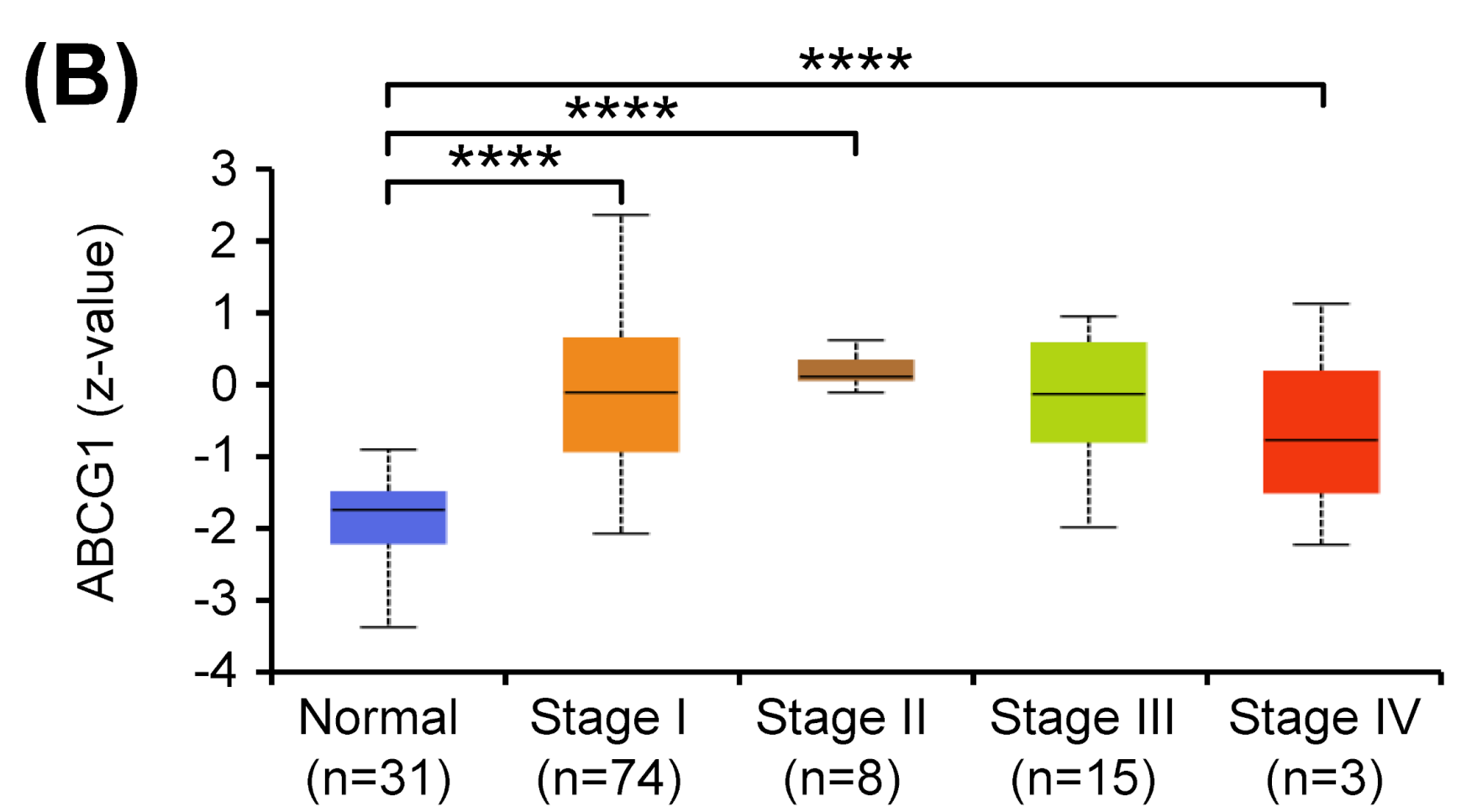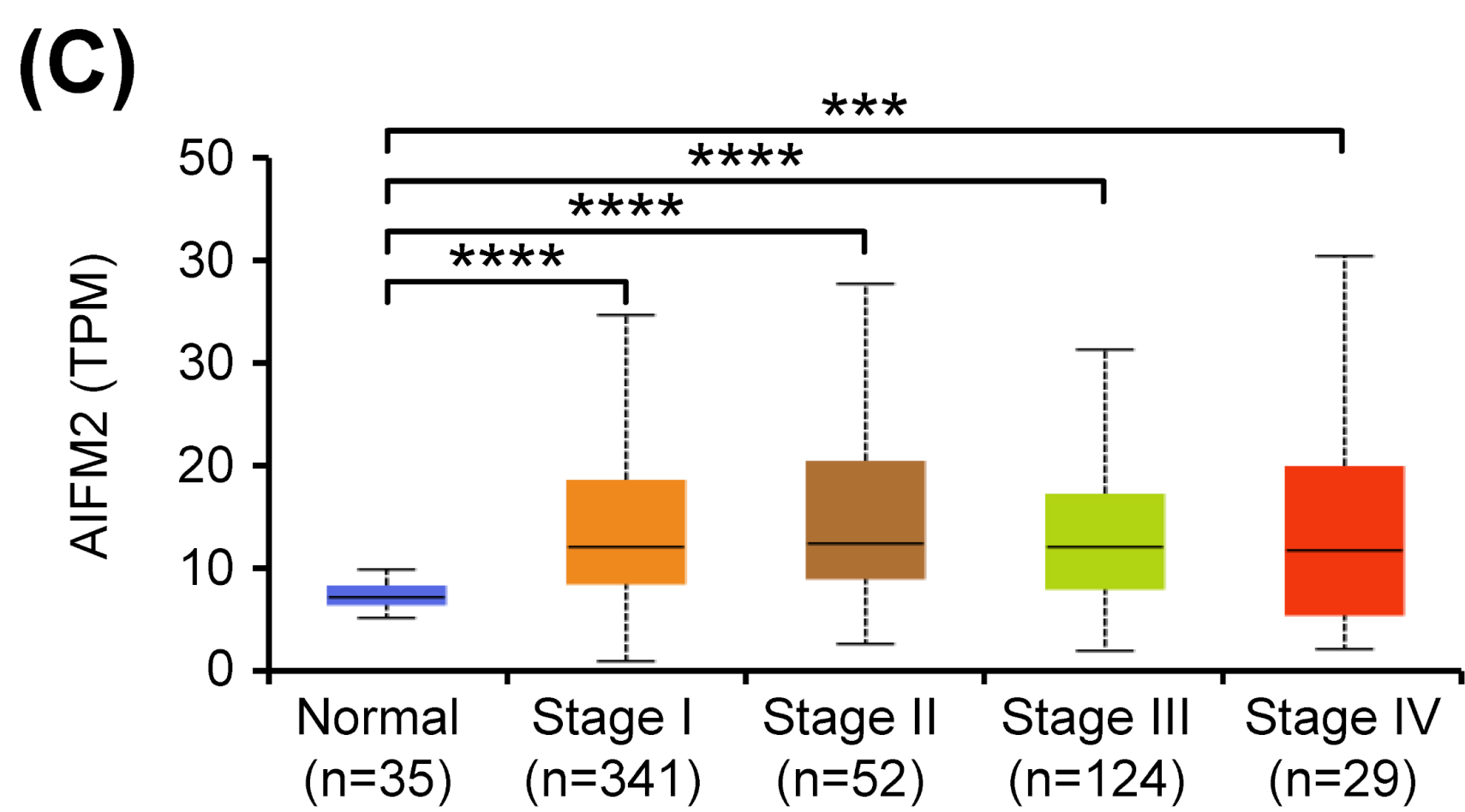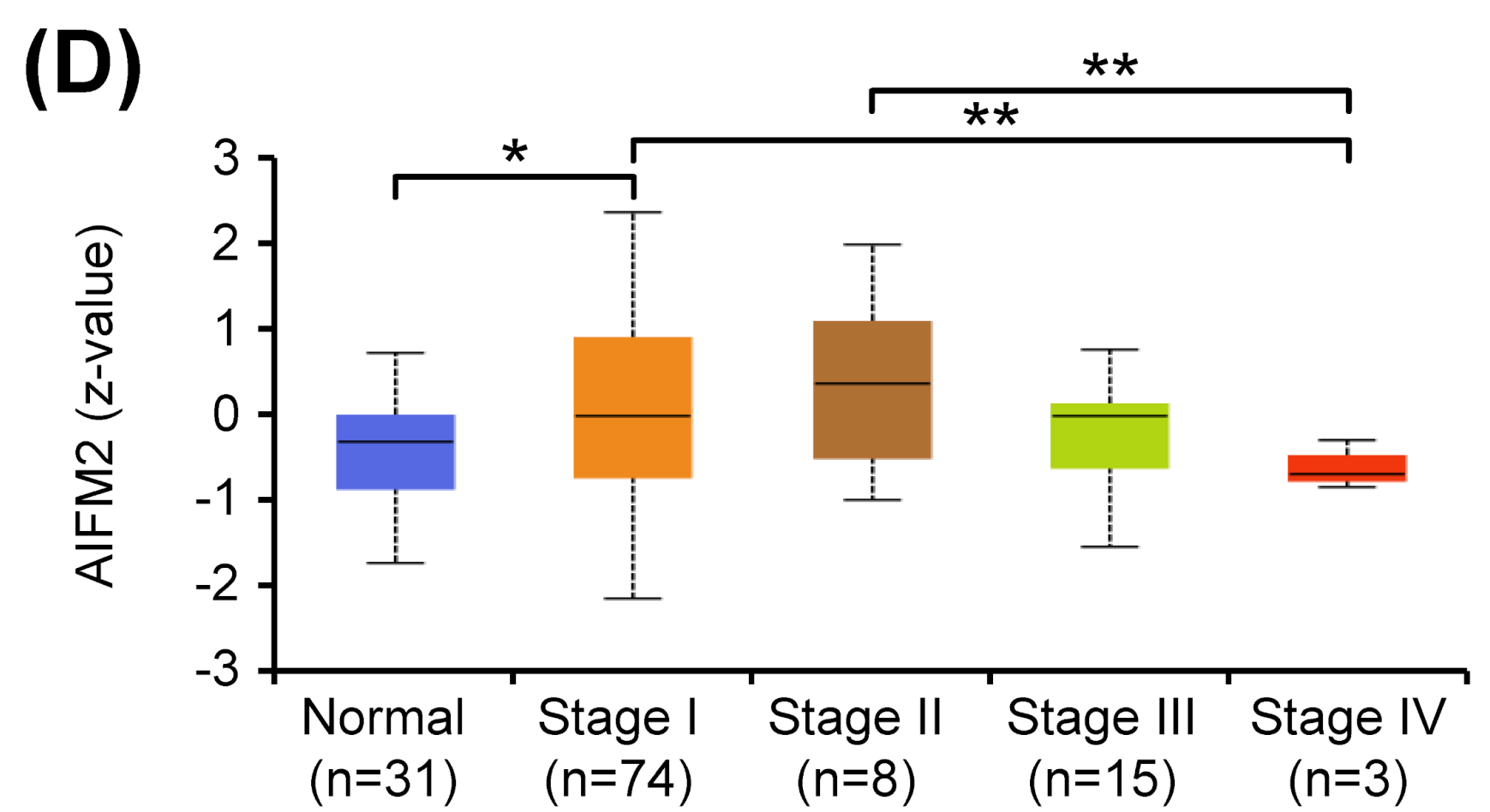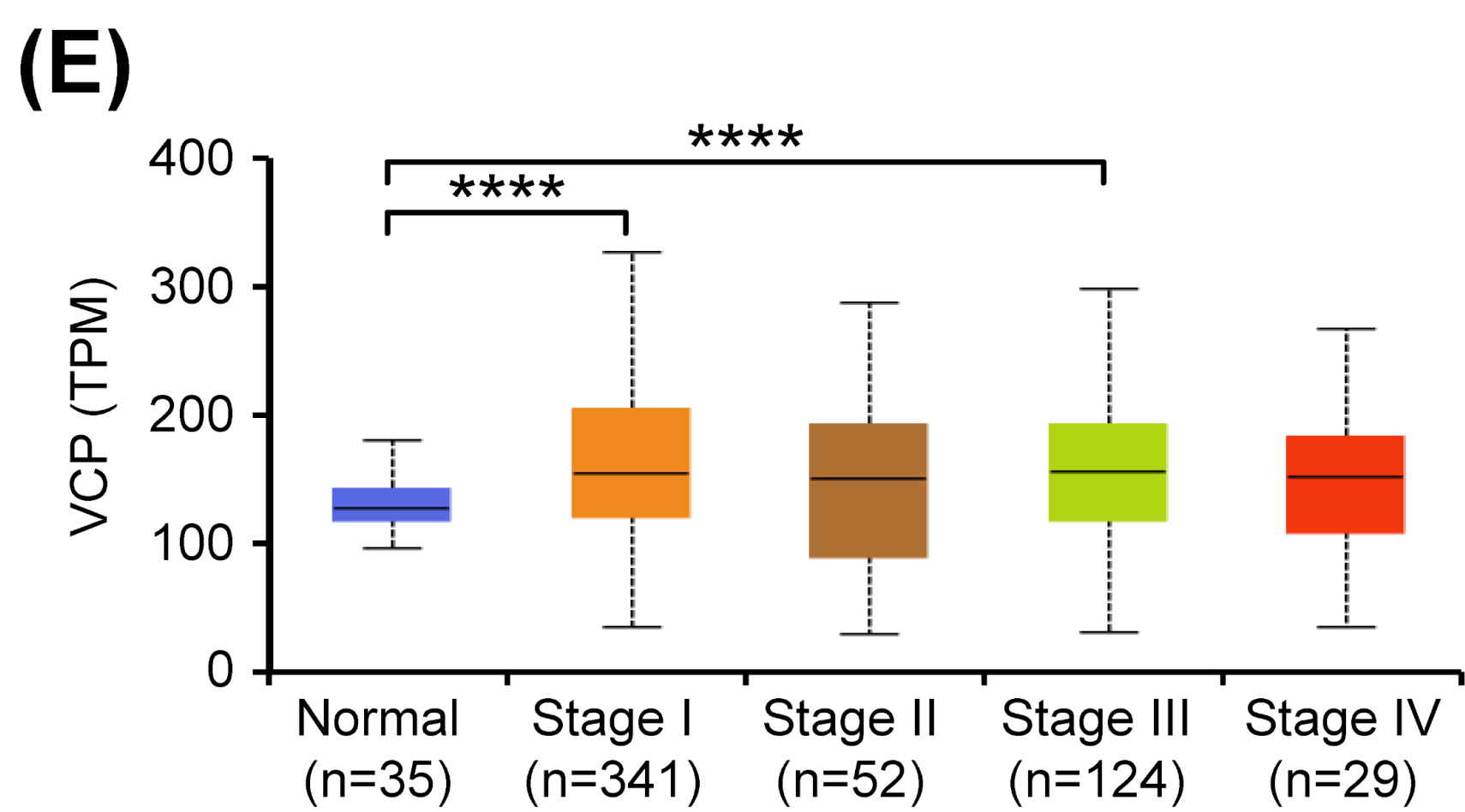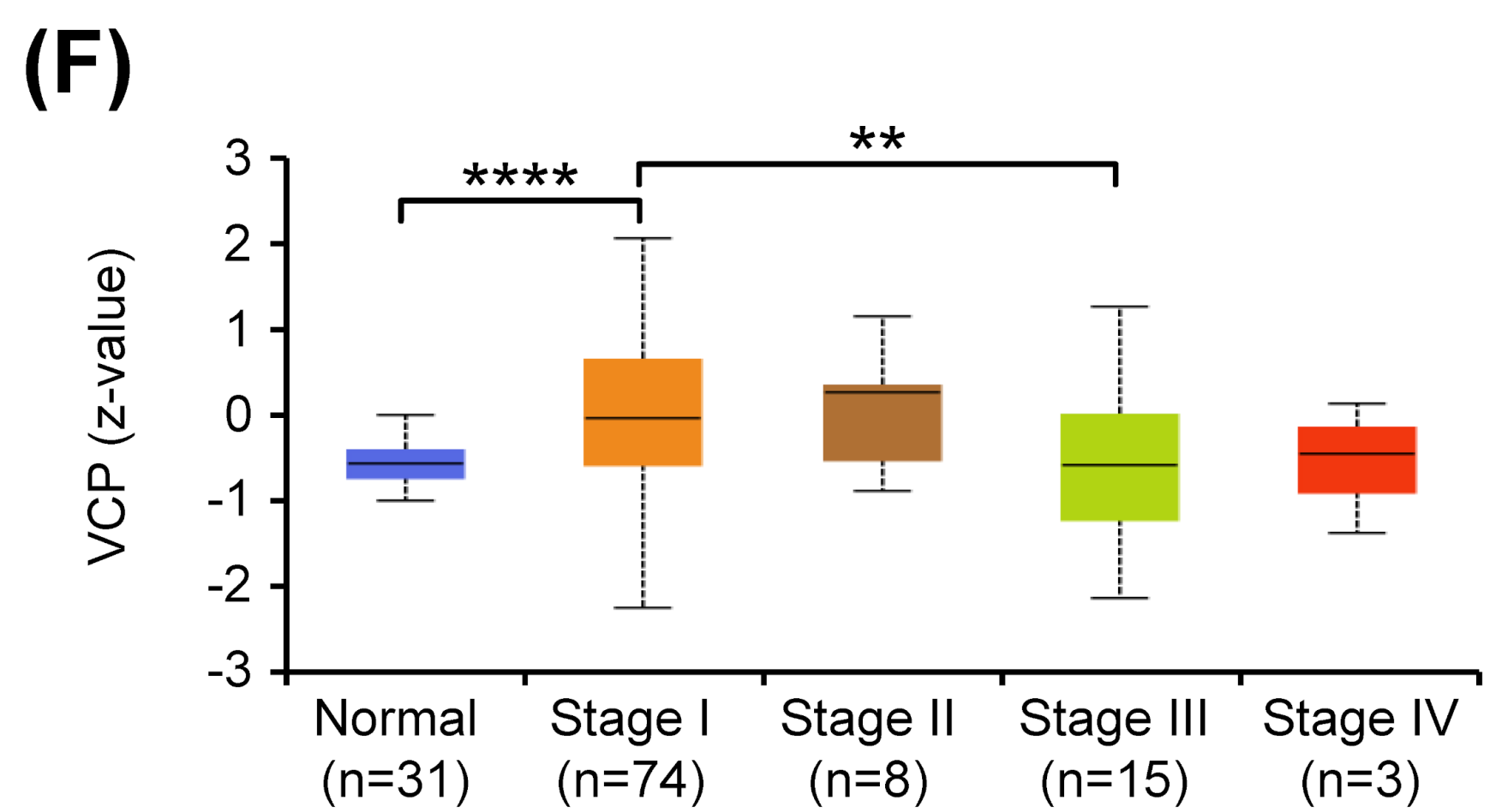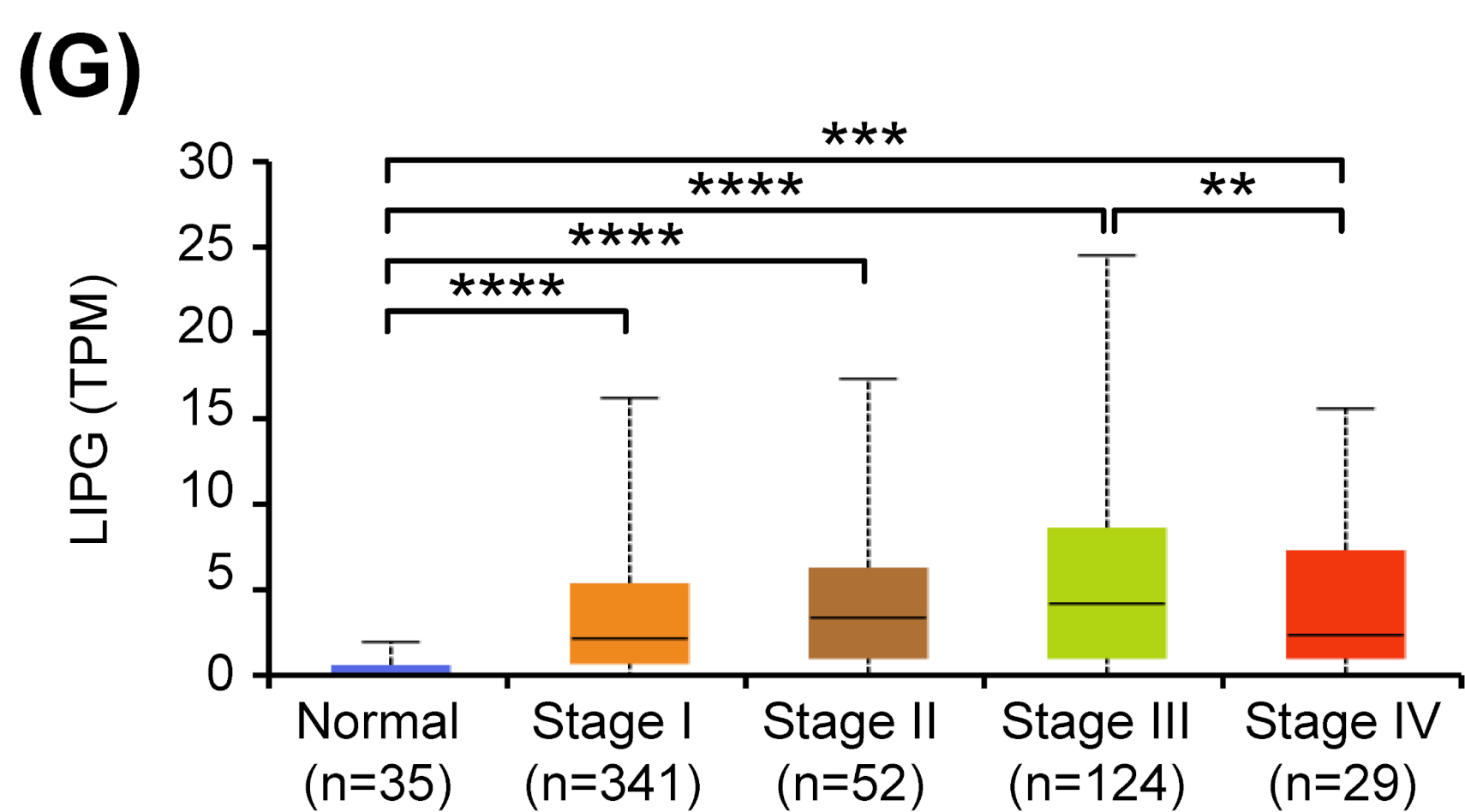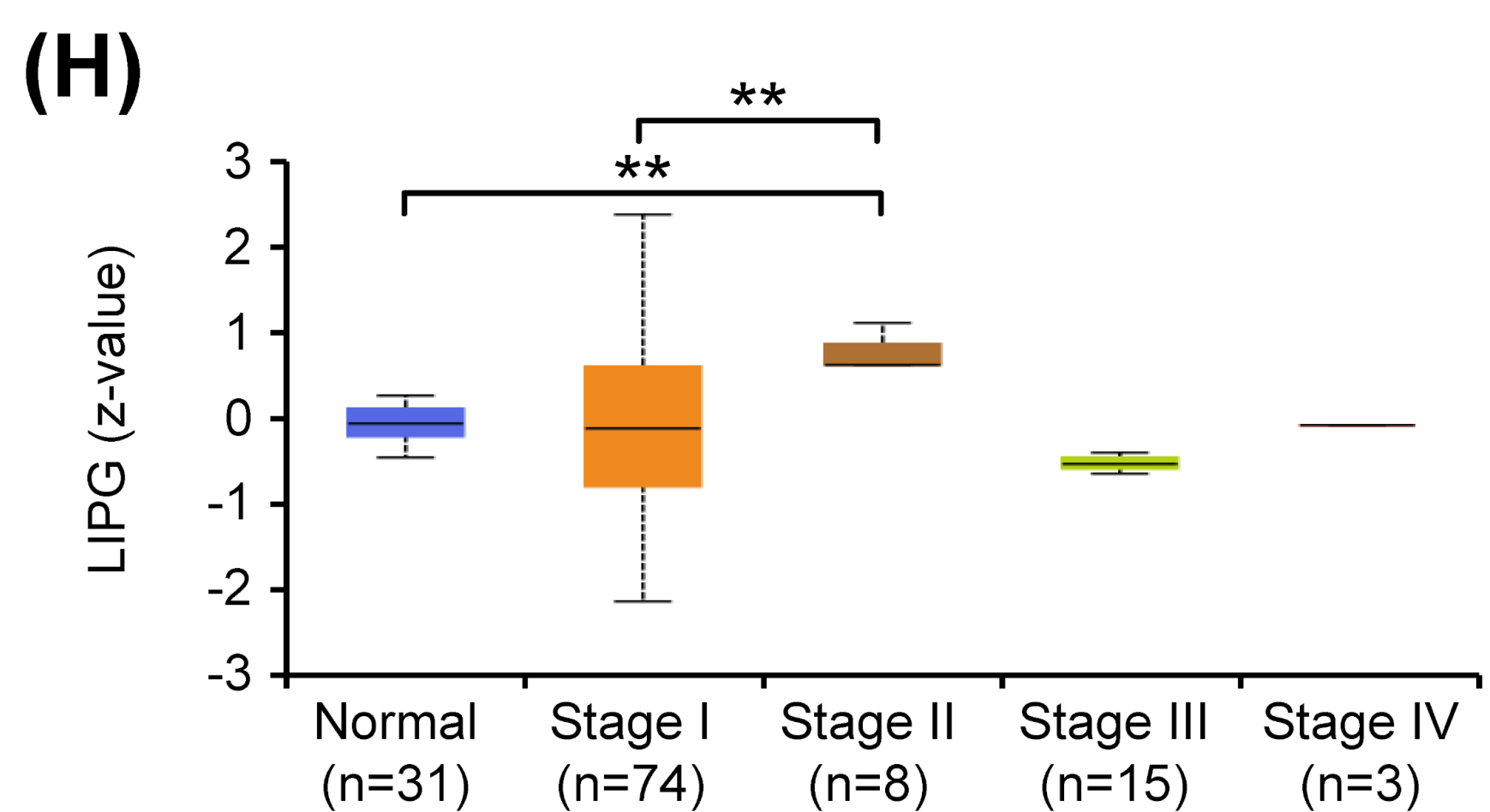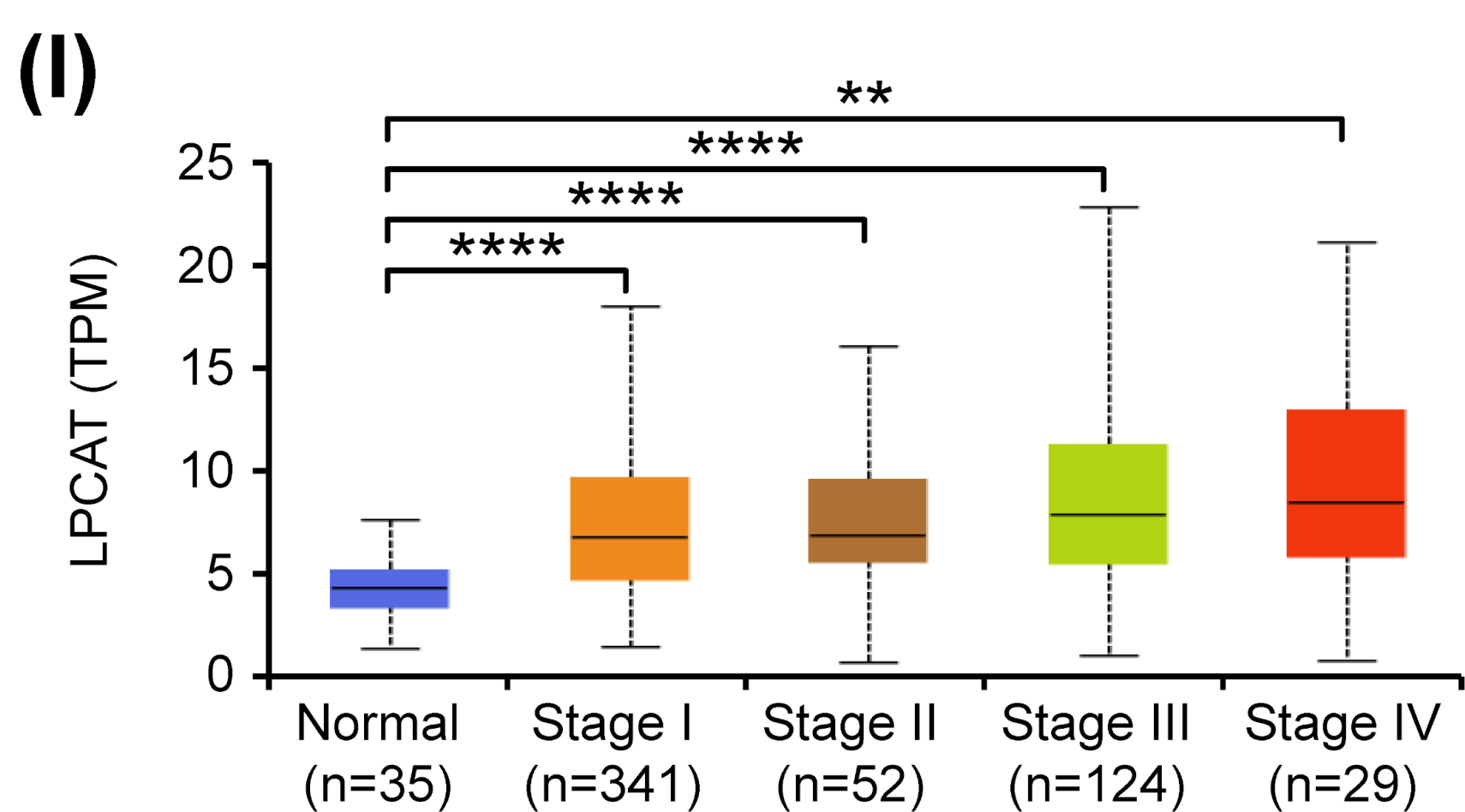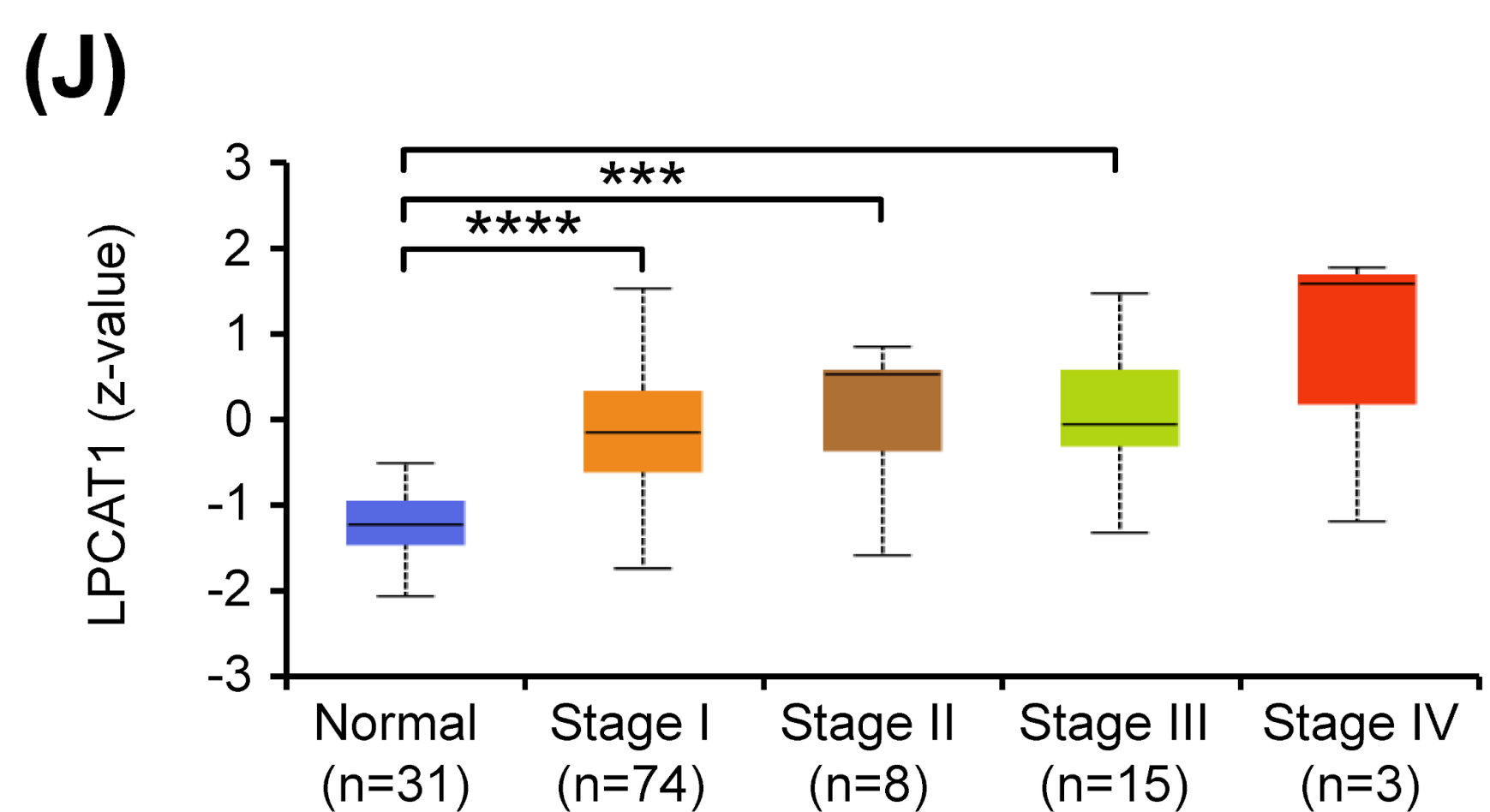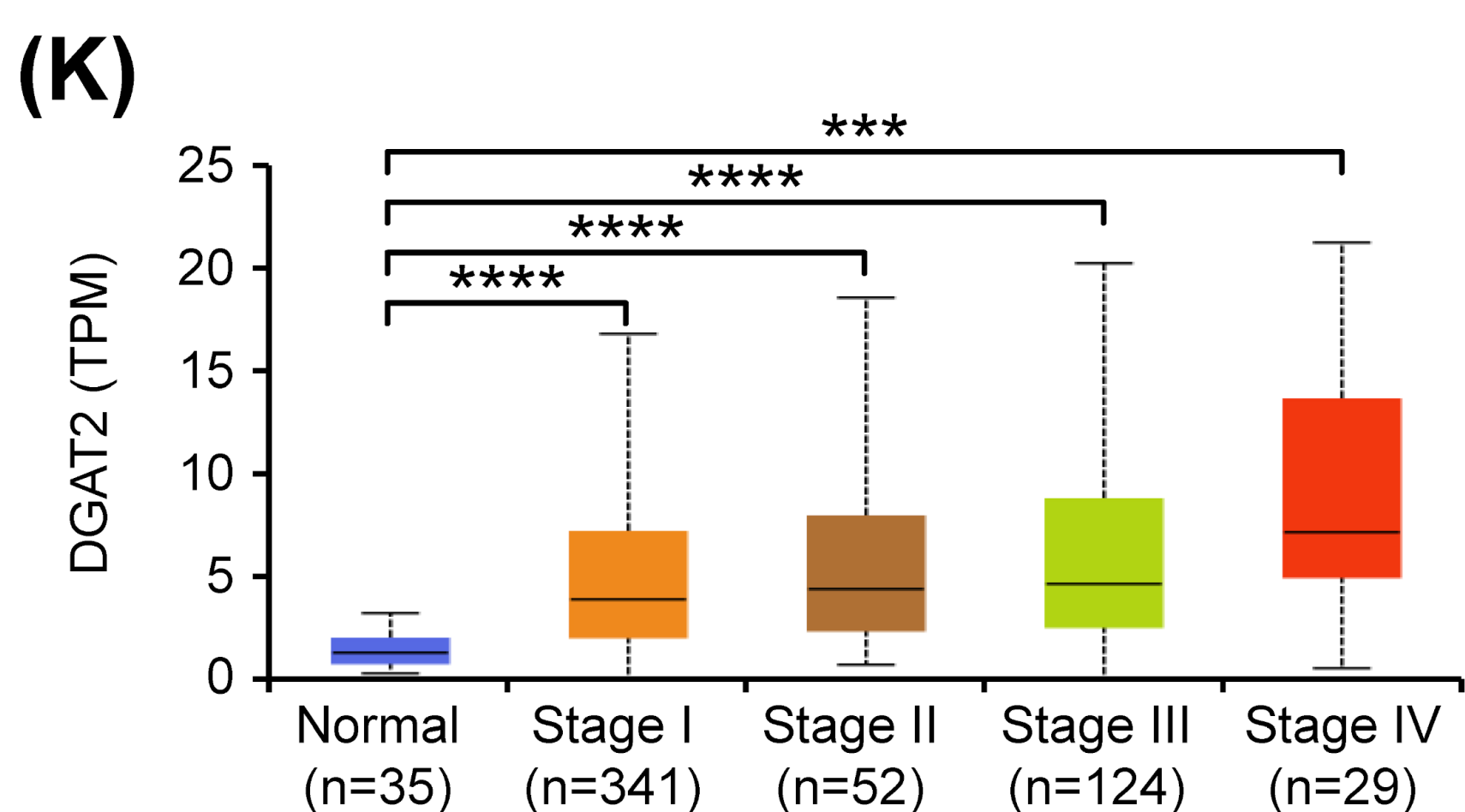

Supplement: Supplementary file 7 — Figure S7: Stagewise expression of diagnostic model genes in endometrial cancer. (A, C, E, G, I, K) mRNA expression and (B, D, F, H, J) protein levels of six diagnostic genes in endometrial cancer, based on UALCAN data. Student “t” test; *p < 0.05, **p < 0.01, ***p < 0.001 and ****p < 0.0001. [file CNR2-8-e70313-s007.pdf]
